# Whole-genome analysis of *Lysinibacillus boronitolerans* MSR1: A dairy-isolated multidrug-resistant and non-pathogenic strain

**DOI:** 10.1371/journal.pone.0333844

**Published:** 2025-12-12

**Authors:** Md. Shahedur Rahman, Md. Tofidul Islam, Mahadi Hasan, Md. Tariquzzaman, Nure Sharaf Nower Samia, Md. Mahfuzur Rahman, Mustafizur Rahman

**Affiliations:** 1 Department of Genetic Engineering and Biotechnology, Jashore University of Science and Technology, Jashore, Bangladesh; 2 Bioinformatics and Microbial Biotechnology Laboratory, Department of Genetic Engineering and Biotechnology, Jashore University of Science and Technology, Jashore, Bangladesh; 3 One Health Laboratory, Infectious Diseases Division (IDD), icddr, b, Dhaka, Bangladesh; 4 Genome Center, Infectious Diseases Division (IDD), icddr, b, Dhaka, Bangladesh; Florida A&M University: Florida Agricultural and Mechanical University, UNITED STATES OF AMERICA

## Abstract

*Lysinibacillus boronitolerans* MSR1, a bacterium isolated from yogurt, was characterized through a detailed genomic and phylogenetic analysis. The strain demonstrated the ability to grow in MRS broth at pH 5–8 and exhibited positive activity in citrate utilization, catalase, oxidase, Methyl Red–Voges Proskauer (MR-VP), and Triple Sugar Iron (TSI) tests while being negative for amylase degradation and sulfide production. Whole-genome sequencing and Average Nucleotide Identity (ANI) analysis revealed a close genetic relationship between MSR1 and previously reported *L. boronitolerans* strains, with ANI values ranging from 95.25% to 98.30%, particularly for *L. boronitolerans strain* NBRC103108. Phylogenetic analyses based on whole-genome and 16S rRNA sequences confirmed the taxonomic placement of MSR1 within the *L. boronitolerans* species. A circular genome comparison highlighted the presence of unique genomic regions in MSR1, notably around the 3500 kbp mark, indicating the acquisition of novel genes that may contribute to its distinct phenotypic traits. Antibiotic susceptibility testing revealed a high level of resistance in MSR1 to glycopeptides and aminoglycosides, while the strain remained susceptible to imipenem, with in silico analysis identifying key antimicrobial resistance (AMR) genes, including *qacJ*, *vanW*, *vanT*, and *FosBx1*, which confer resistance to disinfectants, vancomycin, and fosfomycin through efflux pumps and target modification mechanisms. Five distinct biosynthetic gene cluster (BGC) regions were identified in the MSR1 genome, encoding genes for lanthipeptide-class-iii, RiPP-like, T3PKS, beta-lactone, terpene, and NRPS-like clusters. Pan-genome analysis suggested that *L. boronitolerans* possesses an open pan-genome, with a substantial proportion of accessory and unique genes. Functional annotation of core, accessory, and unique genes revealed that core genes are predominantly associated with metabolic processes, while accessory and unique genes are involved in information processing, storage, and defence mechanisms. These findings enhance our understanding of the genomic diversity, evolutionary dynamics, and potential adaptive strategies of *L. boronitolerans* MSR1, providing new insights into its ecological and functional roles.

## Introduction

Dairy products, especially yogurt, are essential sources of nutrition for children and adolescents as they contain the necessary proteins, fats, amino acids and minerals such as calcium and phosphorus [1]. Yogurt is a fermented dairy product that is rich in nutrients and typically produced using a symbiotic culture of Lactic Acid Bacteria (LAB) [2–4]. These beneficial microorganisms, such as *Lactobacillus* and *Bifidobacterium* species, play a crucial role in fermentation and contribute to yogurts health benefits [5–7]. Although the yogurt is generally a healthy bacterial culture, it may be contaminated by non-starter or environmental microorganisms, which affects the quality of the product and its safety [8].

However, there have been instances where undesirable bacteria, such as *Bacillus paralicheniformis* and *Lysinibacillus boronitolerans*, are found in yogurt as adulterants. These bacteria are not typically present in yogurt and can pose health risks [9]. Recent studies have documented the contamination of dairy products with non-starter bacteria, including *Bacillus* species, which can cause spoilage and pose health risks due to toxin production [10]. *Bacillus licheniformis* can cause antibiotic-resistant sepsis in immunocompetent patients [11]. *Lysinibacillus boronitolerans*, a rod-shaped, spore-forming bacterium, is known for its high tolerance to boron and its ability to grow in diverse environmental conditions, including high pH levels and various temperatures [12,13].

Their isolation has been reported from fish liver, plant tissue, seed, and some fermented products. *L. sphaericus* strain C3-41, an insecticidal bacterium, was the first isolated bacteria *of Lysinibacillus* genus [12]. Several strains of the *Lysinibacillus* genus, isolated from the environment, have been reported as bio-control agents [14]. *L. boronitolerans* is known for microbial-induced calcite precipitation (MICP), which helps in the self-healing of concrete cracks [15]. There are also reports of some strains being utilized in the biosynthesis of nano-particles [16]. Some strains also have the ability to generate bacteriocins, which are effective inhibitors of growth of pathogenic bacteria, including *Bacillus velezensis*, *Bacillus pumilus*, *Xanthomonas axonopodis* and *Pseudomonas syringae* [17]. There are no direct reports of clinical involvement or pathogenicity in humans associated with *L. boronitolerans*. However, *L. sphaericus* has occasionally been linked to human infections, including bacteremia in immunocompromised individuals, severe sepsis and triple-valve [18–21]. Other species of *Lysinibacillus* also exhibit diverse biotechnological potentials, including bio-control agents and functional properties. Among them, *L. sphaericus* exhibits broad-spectrum insecticidal activity, effectively controlling mosquito vectors including *Culex*, *Anopheles*, *Mansonia*, and *Aedes* species, as well as other pests like cockroaches, cutworms, and nematodes [22]. These capabilities highlight its significance in environmentally friendly pest-management strategies and its relevance in applied microbiology.

Recent research has reported the presence of *Lysinibacillus* species in other fermented foods, and this has led to the realization of their possible interactions as part of the food microbiota. For instance, *L. boronitolerans* F1182 was isolated from a traditional Korean fermented soybean product, where it was analyzed for its potential benefits in food and agriculture industries [12]. Similarly, *L. irui* has been identified in fermented locust beans from Africa, highlighting its adaptation to diverse food environments [23]. Despite its presence in some traditional fermented foods, *L. boronitolerans* has also been reported as an adulterant in yogurt, as observed in a recent study from Pakistan [9].

The presence of such bacteria in yogurt highlights a significant research gap. While the genomic sequences of several *Lysinibacillus boronitolerans* strains are available in the NCBI Genome Database (https://www.ncbi.nlm.nih.gov/home/genomes/), the variations among these strains have not been thoroughly explored. The significance of understanding these variations is that microbial strains of identical species may differ significantly in allelic and gene products [24]. This diversity is often regulated by bio-synthetic gene clusters (BGCs: gene groups responsible for the production of secondary metabolites), which are groups of genes responsible for producing secondary metabolites [25,26]. For example, the comparative analyses of *L. pakistanensis* LZH-9 have revealed metabolic versatility, xenobiotic bio-degradation genes, resistance to toxic compounds, and salinity tolerance [27]. Another novel strain, *L. boronitolerans* QD4, has been isolated and characterized for quantum dot biosynthesis, indicating its potential in nanotechnology applications [28].

The studies in this area are urgent because of a number of reasons. To begin with, it is possible to define and describe the genetic differences among bacterial strains and create superior quality control strategies to produce yogurt. Second, the knowledge of how these bacteria obtain new genes, be it by horizontal gene transfer or mutations can give information on how to avoid contamination. Finally, the genome analysis with the help of bioinformatics tools can contribute to the ability to differentiate between similar strains of bacteria, which makes dairy products safe and of high quality. Furthermore, the isolation of *L. boronitolerans* MSR1 of the yogurt provides evidence of its ability to be a contaminant or unrecognized member of the yogurt microbiota and suggests something about the interactions of microbes in food.. Genomic analysis could shed light on unique genomic regions and bio-synthetic gene clusters with potential industrial applications and adaptive mechanisms. These findings will contribute to our understanding of microbial ecology while reinforcing the importance of food safety and public health monitoring.

In this study, our primary objective is to isolate a new bacterial strain from yogurt, which was not commonly present in yogurt microbiota. While some studies have identified *Lysinibacillus boronitolerans* as a potential adulterant, our focus is to conduct a detailed genomic analysis and comparative evaluation with other reported strains using a pan-genome approach. Additionally, analyzing the presence of antimicrobial resistance (AMR) genes will help determine the potential implications for food safety and public health. This comparative analysis will help identify genetic variations and potential bio-synthetic gene clusters (BGCs) that contribute to the unique characteristics and potential pathogenicity of the strains.

## Method and materials

### Sample collection and isolation of bacteria

To isolate bacterial strains from yogurt, freshly prepared yogurt was collected from the market. A diluted yogurt sample was created by suspending 1 mg of yogurt in 9 ml of sterilized distilled water. This initial sample was further diluted to a concentration of 10^−5^ using the serial dilution technique. The 10 ⁻ ⁵ dilution was selected based on preliminary trials, ensuring optimal single colony growth while preventing excessive overcrowding or under-representation [29].

Next, 25 µl of the diluted sample was spread onto De Man–Rogosa–Sharpe (MRS) agar plates (HIMEDIA, Lot-0000572999, GM641-500G, Maharashtra, India) without any added selective agents. MRS agar was used to support the growth of the diverse microbial community naturally present in yogurt. The plates were then incubated overnight at 37°C in a Memmert incubator (Germany). After the incubation period, pure single colonies were obtained by repeated streaking from the colonies that had grown on the MRS agar plates. This streaking process was repeated as necessary to ensure the isolation of pure bacterial colonies [29].

### Physiological, morphological and biochemical characterization of isolate

The morphological surface of the isolate was analyzed using scanning electron microscopy at the Genome Center of Jashore University of Science and Technology (https://just.edu.bd/pages/genome-center) with the Carl Zeiss Sigma 300 SEM (Carl Zeiss Microscopy GmbH, Germany). Bacterial cells from 3 mL of cultured broth were centrifuged at 16,000 rpm, re-suspended in PBS (pH 7.2), and fixed in 2.5% glutaraldehyde for 2 hours. After graded ethanol dehydration (50%−100%) and hexamethyldisilazane (HMDS) treatment (33%−100%), the cells were air-dried overnight, sputter-coated with gold-palladium, and imaged [30]. To gain preliminary insights into the metabolic and enzymatic characteristics of the isolated bacteria, a series of morphological and biochemical tests were performed based on standard protocols. These tests included gram staining, citrate utilization, amylase activity, methyl red-Voges Proskauer, SIM (sulfide, indole, and motility), TSI (triple sugar iron), catalase, and oxidase tests (American Society for Microbiology (ASM) Protocols, https://asm.org/browse-by-content-type/protocols) [31]. All tests used colonies from an overnight culture grown on freshly prepared MRS agar plates. The growth of the isolated bacteria at different pH levels (ranging from 5 to 8) was assessed using MRS broth (HiMedia, Lot-0000563124, GM369-500G, Maharashtra, India). Initially, the pH of the broth was adjusted to 5, 6, 7, and 8. Subsequently, 50 µl of the overnight-grown culture was inoculated into each broth, and the cultures were incubated at 37°C in a shaker incubator set to 120 rpm [32]. The use of MRS broth was based on the initial isolation of the strain from MRS agar, ensuring consistency in growth conditions. Bacterial growth was monitored every 2 hours by measuring the optical density at 600 nm using a spectrophotometer (GENESYS 30, Thermo Scientific, USA) [33,34]. GraphPad Prism (v. 8.0.2) (https://www.graphpad.com/) was employed to generate the growth curve. To ensure reproducibility, the procedure was performed three times.

### Antibiotic susceptibility of isolated strain

To assess antibiotic susceptibility, the disc diffusion method was employed following the general procedures outlined by the Clinical and Laboratory Standards Institute (CLSI) guidelines [35,36]. An overnight grown broth culture was adjusted to match the 0.5 McFarland Standard and spread onto freshly prepared Muller Hinton agar (MHA) plates (HIMEDIA, Lot-0000397849, M173-600G). Antibiotic discs containing amoxy/clav (augmentin) (30 µg), amoxicillin (10 µg), azithromycin (30 µg), ampicillin (25 µg), cefepime (30 µg), cefixime (5 µg), cefotaxime (30 µg), ceftriaxone (30 µg), cefuroxime (30 µg), cephalexin (30 µg), ciprofloxacin (5 µg), colistin (10 µg), co-trimoxazole (25 µg), doxycycline (30 µg), erythromycin (15 µg), imipenem (10 µg), levofloxacin (5 µg), tetracycline (30 µg), nalidixic acid (30 µg), gentamicin (10 µg), vancomycin (30 µg), methicillin (5 µg), neomycin (30 µg), norfloxacin (10 µg), and streptomycin (10 µg) were placed on the agar surface. The plates were incubated overnight at 37°C, and the inhibition zones were measured using a millimeter scale. To ensure reproducibility, the procedure was performed three times, and the zone of inhibition was reported as the mean ± standard deviation (SD). *Staphylococcus aureus* was used as the reference strain. This method allowed for a comprehensive evaluation of the bacteria’s resistance to various antibiotics.

### 16S rRNA gene amplification

The 16s rRNA (16S ribosomal RNA) gene sequencing was conducted at the Virology Laboratory, icddr’b, Mohakhali, Dhaka, Bangladesh. Bacterial DNA was extracted using the QIAamp® DNA Mini Kit (Qiagen, Hilden, Germany) following the manufacturer’s protocol. The extracted DNA was quantified and assessed for purity using a Qubit 1 × double-stranded DNA (dsDNA) high-sensitivity assay kit (Thermo Fisher Scientific, USA) with a Qubit 4 fluorometer (Thermo Fisher Scientific, USA). The 16S rRNA gene was amplified using universal primers 27F (5′-AGAGTTTGATCCTGGCTCAG-3′) and 1492R (5′-GGTTACCTTGTTACGACTT-3′), which target conserved regions of the bacterial 16S rRNA gene of the 30S subunit. The primers were selected as they are universal primers for 16S rRNA gene amplification, widely used for bacterial identification [37].

PCR amplification was performed using the GoTaq® G2 Hot Start Taq polymerase kit (Promega Corp.,WI, USA) in a final reaction volume of 20 µL. The PCR master mix contains 4 µL of 10 × buffer,1 µL of MgCl_2_ (10mM), 0.1 µL of Taq polymerase, 0.4 µL of dNTPs, 1 µL of each primer (10µM), 10.5 μl of nuclease-free water, and 2 µL of extracted bacterial DNA as template. The PCR cycling conditions were as follows: an initial denaturation at 95°C for 15 minutes, followed by 35 cycles of denaturation at 94°C for 30 seconds, annealing at 55°C for 45 seconds, and extension at 72°C for 1 minute 30 seconds, with a final extension at 72°C for 7 minutes. The amplified products were verified by agarose gel electrophoresis using a 1.5% agarose gel stained with SYBR Safe (Thermo Fisher Scientific), and visualized under UV light. Both positive and negative controls were included during the gel electrophoresis to confirm the specificity and accuracy of the 16s gene PCR amplification (S5 Fig).

### Whole genome sequencing

The entire genome sequencing was conducted at the Genomic Centre, icddr’b, Mohakhali, Dhaka, Bangladesh. For DNA extraction from a single bacterial isolate, the Qiagen DNeasy Blood & Tissue Mini Kit (250) (Qiagen, Cat. No. 69504) was utilized. The extracted DNA’s quality was evaluated for suitability for subsequent whole genome sequencing (WGS) using both Nanodrop and Qubit measurements. To proceed with DNA purification, the bacterial pellet went through centrifugation at 14000 rpm for 5 minutes. The DNA purification protocol followed a modified version of the CDC PULSENET Total DNA Extraction protocol (https://www.aphl.org/programs/global_health/Documents/PNL33_DNA_Extraction_and_Quality.pdf), employing the Qiagen DNeasy Blood & Tissue Kit for efficient purification of bacterial DNA. The quality of the purified DNA was subjected to rigorous evaluation. A Nanodrop spectrophotometer (NanoDrop Spectrophotometer ND-1000, Thermo Fisher Scientific, Waltham, MA, USA) was used to assess DNA purity. To maintain purity, the A260/280 ratio was kept between 1.8 and 2.0, and the A260/230 ratio was maintained between 2.0 and 2.2. DNA quantification was performed using a Qubit 4.0 Fluorometer (Qubit^TM^ 4 Fluorometer, Invitrogen by Thermo Fisher Scientific, Singapore), yielding an adequate concentration (≥10 ng/µL). Qubit working solution was prepared by mixing Qubit Reagent and Qubit Buffer (Qubit dsDNA HS Assay Kit, Invitrogen by Thermo Fisher Scientific, USA) in a 1:200 ratio, followed by assessment for both the samples and standards using appropriate assay tubes. To ensure accurate DNA quantification and quality, both Nanodrop and Qubit fluorometric assays were used. Nanodrop provided initial quality indicators through A260/280 and A260/230 absorbance ratios, while Qubit specifically quantified double-stranded DNA for precise downstream applications [38]. In cases of measurement discrepancies, Qubit values were prioritized for library preparation and sequencing.

For library preparation, the Illumina DNA Prep Reagent Kit and an automated liquid handler (epMotion 5075) were utilized. Initially 350 ng of genomic DNA at a concentration of 25 ng/µL was used for library preparation. Throughout the process, genomic DNA was fragmented and tagged with adapter sequences. The tagging was performed using DNA Tagmentation Beads using Illumina DNA Prep Tagmentation (M) Beads (Ref 20015880, Illumina, San Diego, USA) following the manufacturer’s standard protocol. For library quality control (QC), DNA concentration was accurately quantified using a Qubit fluorometer, ensuring precise measurement of DNA levels. Additionally, the integrity and fragment size distribution of the DNA library were assessed using a Bioanalyzer (PerkinElmer LabChip GX Touch 24, PerkinElmer, Waltham, MA, USA), which confirmed the presence of high molecular weight genomic DNA, suitable for sequencing. The prepared DNA libraries were subjected to sequencing using the Illumina MiSeq platform. The platform utilizes fluorescently labeled nucleotides (deoxy nucleoside triphosphates) to determine the genetic sequence of DNA fragments. Sequencing was carried out with paired-end 2x150 bp reads, yielding comprehensive genetic information. For sequencing, the prepared libraries were loaded at a concentration of 12 pM. Regarding consumables, we employed the MiSeq Reagent Kit v2 (500-cycle) (MiSeq v2 Reagent Kit, Ref 15033626, Illumina, San Diego, CA 92122, USA) for sequencing, which includes the necessary reagents for a 500-cycle run on the MiSeq System. Sequencing was performed according to the manufacturer’s standard protocol (https://support.illumina.com/downloads/illumina-dna-prep-reference-guide-1000000025416.html), and the raw reads were demultiplexed and converted into FASTQ format using Illumina’s bcl2fastq software v2.20.0 (https://support.illumina.com/sequencing/sequencing_software/bcl2fastq-conversion-software.html). The quality of sequencing was evaluated by optimizing raw data and cluster reads, focusing on read generation and the percentage of reads passing filter criteria.

In summary, the study encompassed three interlinked phases: extraction of a bacterial isolate, library preparation, and subsequent sequencing. Each phase involved distinct protocols and methodologies, contributing to a comprehensive evaluation of the bacterial DNA’s genetic characteristics and quality. Raw paired-end reads from the MiSeq platform were used for further downstream analysis.

### Genome assembly

The quality of raw sequencing reads was assessed using FASTQC v0.9.1 [39]. Low-quality bases were trimmed using Sickle v1.3, with a trimming length set to 20 [40]. Genome assembly was performed using SPAdes v4.0.0 with default parameters, and the assembled genome was further refined using Pilon v1.24 for error correction and polishing [41,42].

### Species identification and genome annotation

The isolated bacterial strain was identified using Whole Genome Sequencing (WGS) data through PubMLST (https://pubmlst.org/multilocus-sequence-typing) and TYPE (STRAIN) GENOME SERVER (https://tygs.dsmz.de/), with all parameters set to default [43,44]. The Prokka annotation tool was used to annotate the bacterial genome. Following annotation, the Proksee web server (https://proksee.ca/) was employed to generate a circular map highlighting the genomic features. This approach ensured a thorough and precise identification and visualization of the bacterial genome [45,46]. The assembled fasta genome was uploaded to the server and customized using the server tools.

### Comparison of whole genome

For genome comparisons among *L. boronitolerans* strains, the BLAST Ring Image Generator (BRIG-0.95-dist) was used with default settings to visualize similarities and differences [47]. The genomic FASTA file was used as input for BRIG (version 0.95) and the analysis was performed following the procedures described in the BRIG user manual (https://beatsonlab.com/softwares/brig/). Overall nucleotide similarities between the isolated strain and other reported strains from the same species were evaluated using Average Nucleotide Identity (ANI) comparison, performed with the OrthoAni Tool (OAT) v.0.90 (https://www.ezbiocloud.net/tools/orthoani) with a default setting [48]. All previously reported genomes of different *L. boronitolerans* strains were retrieved from the NCBI genome database (https://www.ncbi.nlm.nih.gov/datasets/genome/?taxon=309788) for this analysis, where *L. boronitolerans* NBRC_103108 was the reference genome for this species. Two separate entries for *L. boronitolerans* NBRC 10311008 existed in the NCBI database, with one designated as the reference genome; therefore, both were included in the ANI analysis. However, due to 100% similarity between the entries, one was excluded from subsequent analyses. This methodology provided a comprehensive comparison to understand the genetic relationships among the strains. The ANI figure was generated using TBtools-II v2.309, where the ANI matrix was used as an input file [49].

### Identification of SNP

To identify single nucleotide polymorphisms (SNPs) in the genome of the isolated strain, Snippy v4.6.0 (https://github.com/tseemann/snippy) was used with default parameters. Snippy also detects multiple nucleotide polymorphisms (MNPs), deletions (DELs), and insertions (INSs). The genome of *L. boronitolerans* NBRC_103108 (GCF_002200915.1) served as the reference sequence for this analysis. This method ensured a detailed understanding of the genetic variations present in the isolated strain. Snippy works by aligning an assembled genome to a reference sequence, identifying high-confidence variant sites using FreeBayes v1.3.7, and filtering out low-quality variants [50].

### Identification of bio-synthetic gene clusters (BGCs)

To identify bio-synthetic gene clusters (BGCs) in the isolated strain, the antiSMASH web server (https://antismash.secondarymetabolites.org/#!/start) was used with default parameters. AntiSMASH employs a probabilistic algorithm based on hidden Markov models (HMMs) to detect BGC regions in genomes [51]. This analysis provided insights into the secondary metabolite potential of the isolated bacterial strain.

### Horizontally transferred genes (HGT) identification

To detect the presence of horizontally transferred genes in the isolated strain, HGTector2 v2.0b3 was used with default parameters. This tool utilizes a BlastP-based algorithm to identify horizontal gene transfer (HGT) events [52]. The input for HGTector2 was the protein file (.faa) generated from the Prokka annotation. This method enabled the identification of genes acquired through horizontal transfer, providing a deeper understanding of the genetic makeup of the strain.

### Identification of antimicrobial resistance (AMR) gene

AMR gene analysis was crucial for evaluating the strain’s multidrug resistance profile, which has implications for antimicrobial resistance spread in food-related environments [53]. To determine the antibiotic resistance profile of the isolated bacteria, the Comprehensive Antibiotic Resistance Database (CARD) web server (https://card.mcmaster.ca/analyze/rgi) was used. CARD employs homology and SNP models to predict antimicrobial resistance (AMR) and provides results categorized as perfect, strict, or loose hits [54]. The complete genome sequence in fasta format was submitted to the server with high coverage, excluding nudges. All three prediction criteria were selected to ensure a comprehensive assessment of the antibiotic-resistance genes present in the strain. CARD is a well-curated database for AMR gene identification. In the CARD database, “perfect” hits indicate an exact match with known resistance genes, “strict” hits denote high sequence similarity to functionally validated resistance genes, and “loose” hits represent putative resistance genes with lower similarity or partial matches [54]. Proksee web server (https://proksee.ca/) was employed to generate a circular map highlighting the AMR genes [45,46]. The assembled fasta genome was uploaded to the server and customized using the server tools.

### Pangenome analysis

The pan-genome analysis allows for a comprehensive comparison among the selected strains of *Lysinibacillus boronitolerans*, identifying core genes, accessory genes and strain-specific variations [55]. Based on ANI results, *L. boronitolerans* MSR1 and seven previously reported genomes were selected for pangenome analysis. Roary v3.13.0 and BPGA v1.3 tools were used to construct the pangenome with default parameters [56,57]. The input for Roary was the.gff file, generated from the Prokka annotation and the BPGA used the.faa files from Prokka annotation. Additionally, USEARCH v11.0.667 was employed to estimate the core and pan-genome with a default cutoff value of 50%. BPGA generated the COG and KEGG distributions among the *L. boronitolerans* strains as part of its default workflow. In addition, it constructed the core and pan-genome phylogenetic trees to illustrate the evolutionary relationships within the species. This comprehensive analysis enabled a detailed understanding of the genetic diversity within the species [58]. The pan-genome plot was prepared using GraphPad Prism (v. 8.0.2) (https://www.graphpad.com/).

### Plasmid typing and pathogenicity and virulence properties

For plasmid typing of *L. boronitolerans* MSR1, Plasmid MLST (https://pubmlst.org/multilocus-sequence-typing) and Plasmid Finder 2.1 (https://cge.food.dtu.dk/services/PlasmidFinder/) were utilized with default parameters [43,59]. Additionally, Pathogen Finder 1.1 (https://cge.food.dtu.dk/services/PathogenFinder/) and Virulence Finder 2.0 (https://cge.food.dtu.dk/services/VirulenceFinder/) were employed to predict pathogenicity and identify virulence genes, respectively, with server defaults [60–63]. In Virulence Finder 2.0, analysis encompassed all species available on the server, and read type was specified as Assembled Genomes/Contigs, as the genome assembly of the query genome was at the contig level [43]. These tools facilitated a comprehensive assessment of plasmid content and virulence potential in the bacterial strain. The default parameter was set to ensure an unbiased and comprehensive analysis.

## Results

### Characteristics of the isolated strain

**Fig 1:**
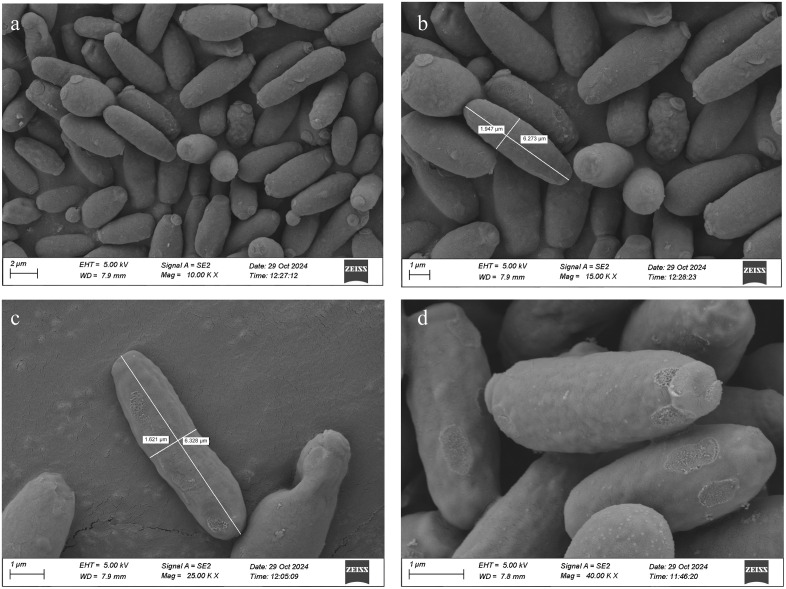
Scanning electron microscopy (SEM) images of *L. boronitolerans* MSR1 showing surface morphology at different magnifications. (a) 10,000x, (b) 15,000x, (c) 25,000x, and (d) 40,000x.

The bacterium isolated from the yogurt sample was designated as 7NBS and displayed typical characteristics of a gram-positive, rod-shaped organism under the microscope. Its colony on agar was circular and measured between 1 mm and 3.5 mm in diameter with white colour, smooth, entire margin and umbonate elevation. The Scanning Electron Microscopy (SEM) showed that the average length of the bacterial cells was about 6.25 um and a width of 1.75 um (Fig 1). Biochemically, the isolate showed positive results in citrate utilization, catalase activity, oxidase activity and methyl-red – Voges-Proskauer tests. It showed negative results for amylase degradation and sulfide production in the SIM test while testing positive for indole production and motility. Additionally, the TSI test indicated positive results (S1 Fig). These combined morphological and biochemical characteristics provide valuable insights into the identity and metabolic capabilities of the isolated bacterium. Table 1 presents the detailed morphological and biochemical characteristics of the bacterial isolate.

**Table 1:** Morphological and biochemical characteristics of bacterial isolates.

*Name of the test*			*Result*
*Morphological characteristics*	Gram staining		Positive
	Shape		Rod
	Colony shape		Circular
	Colony size		1mm – 3.5 mm
	Colony color		White
	Colony margin		Entire
	Colony elevation		Umbonate
	Colony surface		Smooth
	Cell length		6.25 µm
	Cell width		1.75 µm
*Biochemical characteristics*	Citrate utilization		Positive
	Amylase degradation		Negative
	Catalase activity		Positive
	Methyl-Red test		Positive
	Voges-Proskauer test		Positive
	Oxidase activity		Positive
	SIM test	Sulfide	Negative
		Indole	Positive
		Motility	Positive
	TSI test		Positive

### Growth response to pH variations

The growth dynamics of *L. boronitolerans* MSR1 at various pH levels (5, 6, 7, and 8) over 40 hours reveal distinct patterns. In the initial growth phase of the first 0–6 hours, there was a delay because the bacteria were adjusting to the new environment. During this time, their number was too low to be detected by the spectrophotometer. The growth commences almost immediately at pH 8 where it can be detected after the first 2 hours.. In contrast, growth at pH 7 starts around 4 hours, while at pH 6, it becomes noticeable between 4–6 hours. The growth at pH 5 was delayed, which became visible after 6 hours. During the exponential growth phase (4–12 hours), pH 8 exhibits the highest growth rate with a steep increase in absorbance from 4 to 10 hours. Although pH 7 follows a similar pattern, its growth rate is slightly lower. Both pH 6 and 5 show slower growth rates, with pH 6 displaying moderate growth and pH 5 the slowest.

The growth at pH 8 reaches the stationary stage after approximately 10 hours and remains at a high absorbance (approximately 1.7) for the next 14 hours. The stationary phase starts at a pH of 7 after 12 hours with an absorbance of approximately 1.5. Both pH 6 and 5 stabilize at approximately 12 hours, with the other absorbances being less (approximately 1.2 and 0.8 respectively). In the decline phase (post 24 hours), all pH levels show a reduction in growth, likely due to nutrient depletion, with the decline being more gradual at higher pH levels (7 and 8) compared to the more acidic conditions (5 and 6).

**Fig 2:**
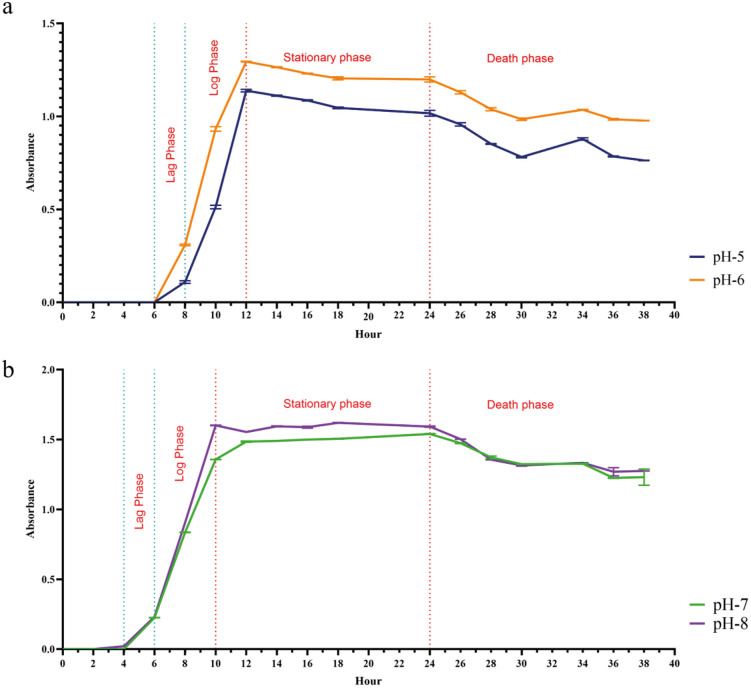
Growth of *L. boronitolerans* MSR1 in different pH (5-8). (a) Growth patterns observed at pH 5 and 6; (b) Growth patterns observed at pH 7 and 8, highlighting variations in developmental stages across conditions.

These findings suggest that *L. boronitolerans* MSR1 thrives best in basic conditions, particularly at pH 8, where it demonstrates the highest growth rate and longest stationary phase. Although yogurt’s pH is much lower than the ideal condition of MSR1’s growth, it can survive the acidic pH condition. Growth is significantly slower and less robust at lower pH levels, especially at pH 5, indicating that maintaining a basic pH is optimal for the growth and metabolic activity of this bacterium (Fig 2) (S1 File). After 24 hours, the optical density (OD) decreases, likely due to the bacterial transition into the death phase, nutrient depletion, or the accumulation of metabolic byproducts.

### Genomic insights and taxonomic classification

**Fig 3:**
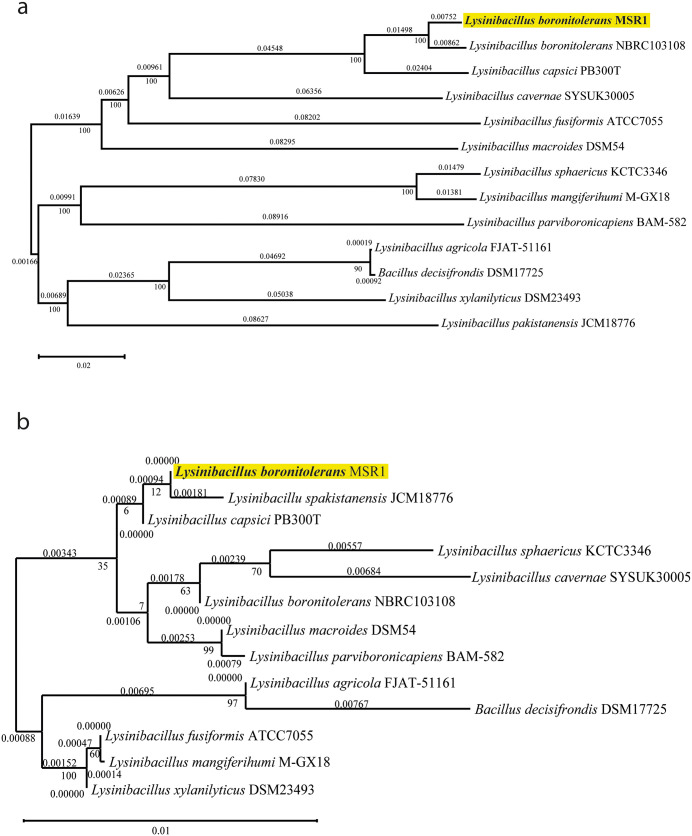
Phylogenetic tree analysis for species identification. (a) Whole genome-based phylogeny; (b) 16s sequence-based phylogeny.

Fig 3 shows two phylogenetic trees, one based on whole-genome sequences (a) and the other based on 16S rRNA sequences (b), used to infer the evolutionary relationships of *L. boronitolerans* MSR1 with other strains and species within the *Lysinibacillus* genus. Both trees highlight the placement of *L. boronitolerans* MSR1 and its close relationship to other strains of *L. boronitolerans*.

*L. boronitolerans* MSR1 is closely related to *L. boronitolerans* NBRC103108 based on a minimal genetic distance in the whole-genome phylogeny (Fig 3a).. These strains form a distinct clade, separating them from other species like *Lysinibacillus capsici* PB300T and *Lysinibacillus fusiformis*. The genome-wide phylogeny further demonstrates that *L. boronitolerans* MSR1 has unique genomic characteristics that differentiate it from other members of the *Lysinibacillus* genus, as seen in their distant grouping.

A similar grouping in the 16S rRNA sequence-based phylogeny (Fig 3b) includes *L. boronitolerans* MSR1 grouping closely with *L. boronitolerans* NBRC103108. However, the phylogenetic tree generated from 16S sequences shows slightly different branching patterns with certain species, like *L. fusiformis* and *L. mangiferihumi*, appearing closer to the *L. boronitolerans* clade than in the whole-genome phylogeny. Despite the minor differences, the overall relationships between strains remain consistent, confirming the validity of species-level identification through both approaches.

Other species, like *Lysinibacillus capsici* PB300T and *Lysinibacillus cavernae* SYSUK30005 are more distantly related to MSR1, as seen by the longer branch lengths and lower prediction confidence values (0.05 and 0.03, respectively). Additionally, species like *Lysinibacillus fusiformis* and *Lysinibacillus sphaericus* form a separate clade, indicating a greater evolutionary divergence from MSR1.

Table 2 presents a summary of the genomic properties of *L. boronitolerans* MSR1 from genome annotation, including its genome size of 4,645,776 base pairs (bp), a GC content of 37.5%, and the number of contigs, coding sequences (CDS), and rRNA and tRNA genes. According to the table, the strain contains 307 contigs, 4560 CDSs, 18 rRNAs, and 111 tRNAs, along with a single tmRNA. Additionally, the genome includes 3 repeat regions and 7 Cas clusters, indicating a potential role for these elements in horizontal gene transfer and immune defense.

**Table 2:** Genomic properties of isolated strain.

*Name of Species*	*Lysinibacillus boronitolerans MSR1*
*Genome Size*	4645776
*GC Content (%)*	37.5
*Contigs*	307
*CDs*	4560
*rRNA*	18
*tRNA*	111
*tmRNA*	1
*Repeat regions*	3
*Cas Cluster*	7

**Fig 4:**
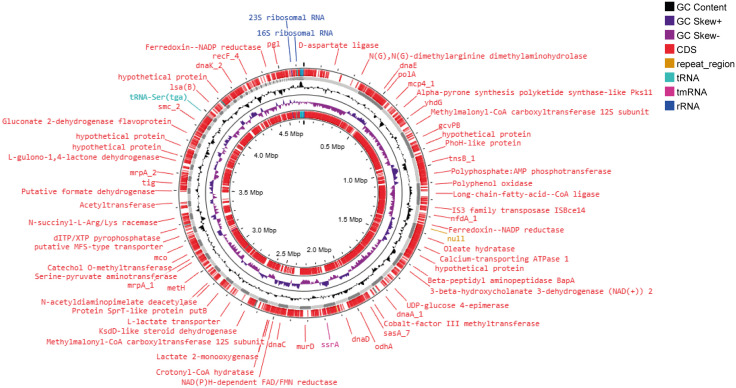
A circular genome map of *L. boronitolerans* MSR1. This map displays various genomic features in a layered circular format. The concentric circles represent different aspects of the genome, starting from the outermost ring, which indicates annotated genes (CDS) and functional elements, while the inner rings show the GC content, GC skew, and repeat regions. Each color denotes a specific genomic feature: GC content is shown in purple, while CDs, tRNA, and rRNA are marked in distinct colors. The annotations around the outer ring point to specific genes and proteins encoded by the genome, including important enzymes like acetyltransferase, hypothetical proteins, and reductases, highlighting functional diversity.

The genome map (Fig 4) highlights the location of important genes, such as ribosomal RNA (rRNA) genes, transfer RNA (tRNA) genes, and regions coding for hypothetical proteins or other key enzymes. The positions of repeat regions and Cas clusters involved in bacterial immunity are also identified in the map. These features are important in understanding the functional capacity and adaptive mechanisms of *L. boronitolerans* MSR1.

### Comparative genomic insights

The genetic relatedness between previously reported strains of *Lysinibacillus boronitolerans* and *L. boronitolerans* MSR1 was assessed using Average Nucleotide Identity (ANI) comparison. ANI values of 95% or higher are considered a threshold to determine whether two bacterial strains belong to the same species. Nine previously reported genomes of *L. boronitolerans* were retrieved from the NCBI database (https://www.ncbi.nlm.nih.gov/datasets/genome/?taxon=309788) for this genomic comparison (S1 File).

**Fig 5:**
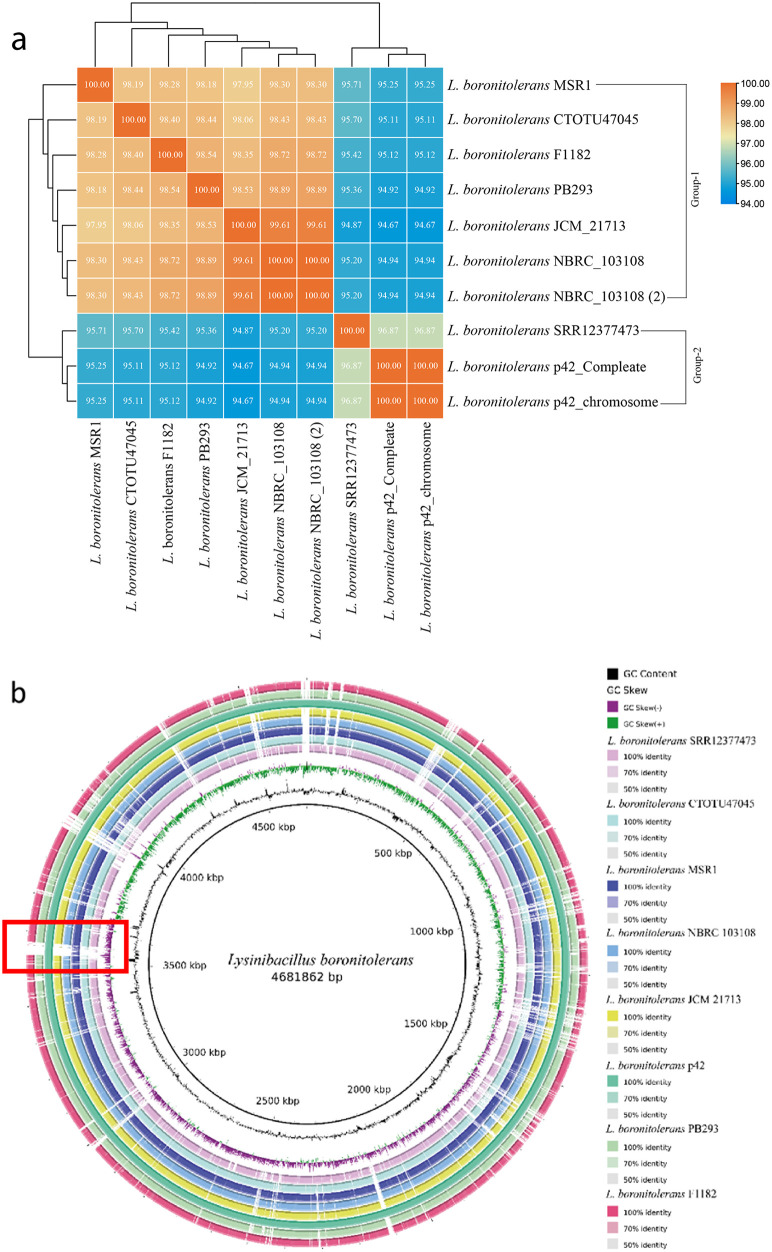
Genome comparison. (a) ANI Matrix. Nucleotide identity comparison between the strains of *Lysinibacillus boronitolerans*. The ligand on the top left indicates a similarity between blue (low) and orange (high). (b) Circular genome comparison of *Lysinibacillus boronitolerans* strains. The red-marked region is the unique region of *L. boronitolerans* MSR1.

The ANI values between *L. boronitolerans* MSR1 and other strains ranged from 95.25% to 98.30%, indicating a high degree of genetic relatedness and confirming that *L. boronitolerans* MSR1 belongs to the same species. Among these, *L. boronitolerans* NBRC_103108 exhibited the highest similarity to MSR1, with an ANI value of 98.30% (Fig 5a). Hierarchical clustering of ANI values grouped the strains into two distinct clusters. *L. boronitolerans* p42 and *L. boronitolerans* SRR12377473 formed the second group, showing relatively lower similarity (94.67% − 95.71%) compared to strains in the first group. *L. boronitolerans* MSR1, residing in group 1, shared a maximum ANI of 95.71% with *L. boronitolerans* SRR12377473 and 95.25% with *L. boronitolerans* p42 (S10 File).

A circular genome comparison was performed to visualize sequence conservation and variability across different genomic regions. This analysis highlighted genomic alignment, visualized in a circular format, where gapped regions and gene absence were represented by white gaps. In *L. boronitolerans* MSR1, a distinct small region (red box, Fig. 5) containing unique sequences was identified at the beginning of the genome, which was absent in all other strains except p42. Additionally, small unique regions were identified near the 400 kbp, 800 kbp, and 1700 kbp genomic positions. A large region containing unique sequences was detected after the 3500 kbp mark in *L. boronitolerans* MSR1. While this region was also found in the p42 strain, it exhibited minor differences, as indicated by small gaps in the alignment (Fig 5b). These results suggest that *L. boronitolerans* MSR1 harbors a significant number of unique genes, potentially contributing to its strain-specific characteristics.

### Genomic variability and SNP distribution

A comprehensive genomic comparison between *L. boronitolerans* MSR1 and *L. boronitolerans* NBRC_103108 revealed a significant degree of genetic variation. In total, 41,498 genomic variants were identified in *L. boronitolerans* MSR1, with 34,736 of these being classified as Single Nucleotide Polymorphisms (SNPs) dispersed throughout the entire genome (Table 3). In addition to SNPs, other forms of genetic alterations were observed, including Multiple Nucleotide Polymorphisms (MNPs), as well as various deletions and insertions. These mutations contribute to the overall genetic diversity observed between the two strains. A detailed list of all detected variants, including their genomic positions, predicted effects, and potential impact on gene function, is provided in S2 File.

**Table 3:** Variants in *L. boronitolerans* MSR1.

*Type of Variant*	*No. of Variant*
*Complex*	6008
*SNPs*	34736
*MNPs*	1
*Deletions*	386
*Insertions*	367
*Total*	41498

### Biosynthetic gene clusters (BGCs)

**Fig 6:**
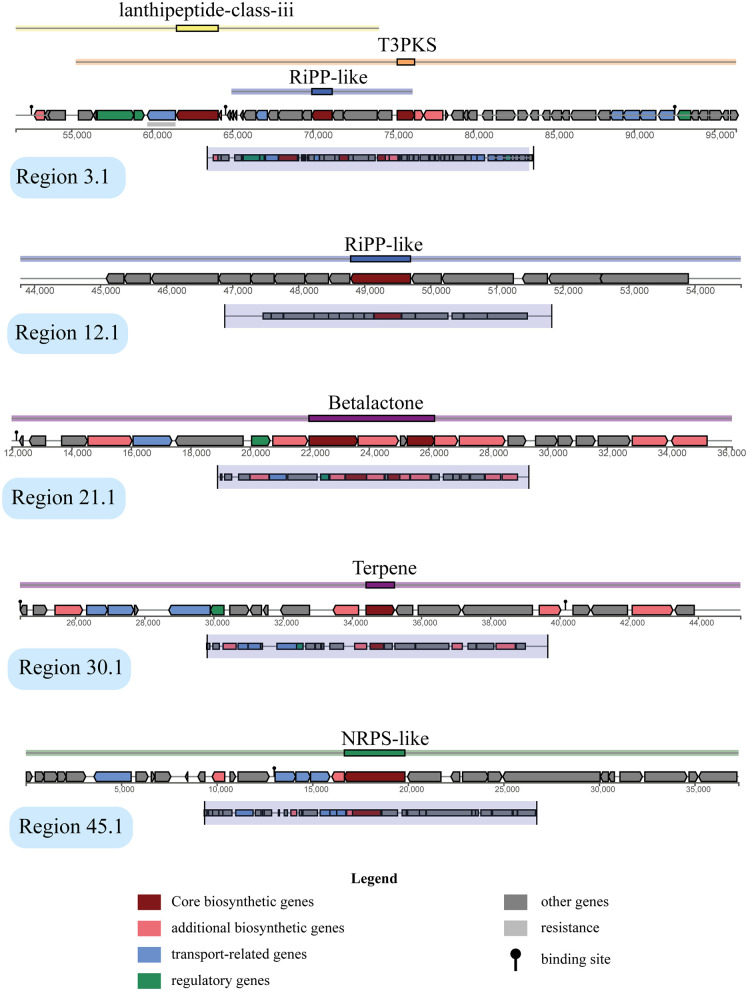
Position of major BGCs. Each region presents a separate position for BGCs in the genome.

Table 4 presents an overview of the major biosynthetic gene clusters (BGCs) identified in the genome of *L. boronitolerans* MSR1, highlighting the type, genomic location, and similarity of these clusters. A total of five distinct BGC regions were predicted, involved in the production of secondary metabolites. The BGCs identified in the MSR1 genome are diverse, including lanthipeptide-class-iii, RiPP-like (ribosomally synthesized and post-translationally modified peptides), T3PKS (Type III Polyketide Synthase), betalactone, and terpene, along with an NRPS-like (non-ribosomal peptide synthetase) gene cluster (Fig 6).

**Table 4:** Major BGC and their position in the genome of 7NBS.

*Serial No.*		Location		Length		*Type*	*Similarity*
*Node No*	Region	Start	End	NT	AA		
*Node 3*	3.1	61,530	64,127	2598	865	Lanthipeptide-class-iii: micKC	30%
		69,955	71,220	1266	421	RiPP-like: YcaO	
		75,266	76,348	1083	360	T3PKS: Chal_sti_synt_C	
*Node 12*	12.1	48,792	49,697	906	301	RiPP-like: Linocin_M18	
*Node 21*	21.1	21,980	23,614	1635	544	Betalactone: AMP-binding	46%
		25,284	26,201	918	305	Betalactone: HMGL-like	
*Node 30*	30.1	34,404	35,225	822	273	Terpene: phytoene_synt	
*Node 45*	45.1	16,666	19,836	3171	1056	NRPS-like: NAD_binding_4	4%

The first BGC (Node 3) includes genes for a class-iii lanthipeptide (micKC), with a relatively low similarity of 30% (Table 4). Additionally, this region harbors RiPP-like genes (YcaO) and T3PKS genes (Chal_sti_synt_C), demonstrating the complexity of this region in producing different types of secondary metabolites. This cluster is responsible for encoding various bioactive compounds, potentially including antimicrobial peptides or polyketides with pharmacological properties (Fig 6).

Node 12 is a simpler cluster, encoding a RiPP-like linocin M18, a bacteriocin-like peptide known for its antimicrobial activity. Similarly, Node 21 contains a beta-lactone biosynthesis cluster, with an AMP-binding domain showing 46% similarity, the highest among all clusters (Table 4). This high similarity suggests that this region may play a crucial role in producing compounds such as β-lactone antibiotics (Fig 6).

In contrast, Node 30 represents a terpene BGC with a phytoene synthase gene, while Node 45 encodes an NRPS-like cluster (NAD_binding_4) but with only 4% similarity, indicating a potentially unique or poorly characterized pathway (Table 4). These variations in BGC similarity and type suggest that *L. boronitolerans* MSR1 has the potential to produce a wide range of bioactive compounds, some of which may be novel or exhibit strain-specific biochemical properties (Fig 6).

### Horizontally transferred genes (HGT)

**Fig 7:**
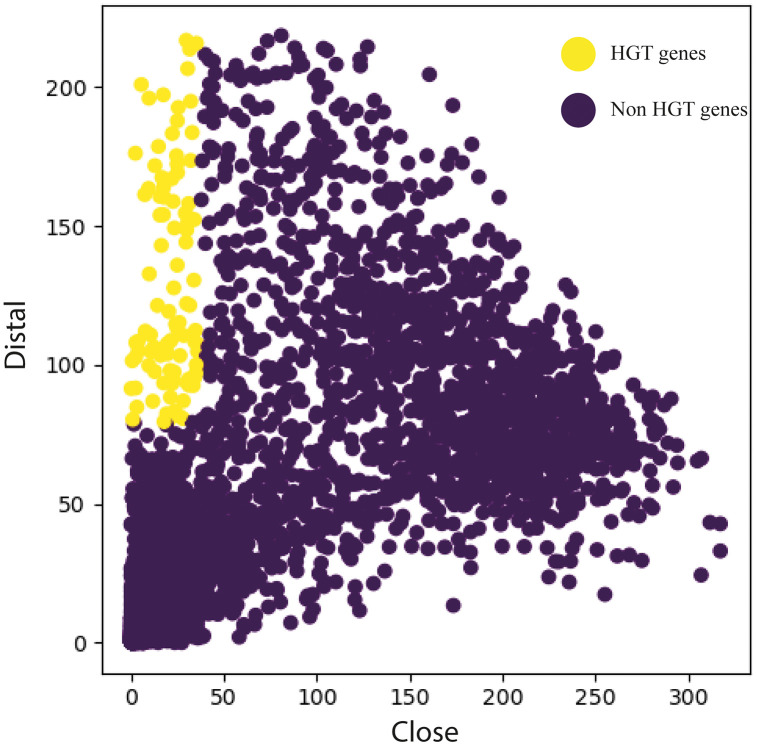
Horizontally transferred genes. **Plot indicating the distributions of “close” and “distal” scores revealed by similarity searches**. The scatter plot was created with HGTector. Potentially transferred genes (*n* = 102) are indicated in yellow.

Table 5 details the number of horizontally transferred genes (HGTs) identified in the genome of *L. boronitolerans* MSR1, categorized by their phylogenetic origin. A total of 102 HGT events were detected (Fig 7), with the vast majority of these genes (90) originating from the phylum *Bacillota*, predominantly from the *Bacilli* class and the *Bacillales* order (S3 File). These HGT genes were transferred from several genera, including *Viridibacillus*, *Ureibacillus*, *Sporosarcina*, *Rummeliibacillus*, and *Metasolibacillus*. Among these, *Planococcaceae* family contributed the largest number of HGTs, with 42 genes. Notably, several genes have unidentified origins within *Bacillota*, with 27 genes labeled as “Not known,” suggesting either novel or poorly characterized sources of gene transfer.

**Table 5:** Number of horizontally transferred genes (HGT) detection.

Phylum	Class	Order	Family	Genus	Species	No. of gene
Bacillota (90)	Bacilli (81)	Bacillales (79)	Planococcaceae (42)	*Viridibacillus (4)*	–	4
				*Ureibacillus (3)*	*Ureibacillus xyleni* (2)	2
					Not known (1)	1
				*Sporosarcina (1)*	–	1
				*Rummeliibacillus (1)*	*Rummeliibacillus pycnus*	1
				*Metasolibacillus (6)*	–	6
				Not known (27)	–	27
			Paenibacillaceae (9)	*Paenibacillus (2)*	–	2
				*Brevibacillus (1)*	*Brevibacillus daliensis*	1
				Not known (6)	*–*	6
			Not known (28)	–	*–*	28
		Not known (2)	–	–	*–*	2
	Tissierellia (1)	Tissierellales	Tissierellaceae	*Tissierella*	*Tissierella pigra*	1
	Not known (8)	–	–	–	*–*	8
Pseudomonadota (1)	–	–	–	–	–	1
Not known (11)	–	–	–	–	–	11

Other notable transfers included genes from the family *Paenibacillaceae*, contributing 9 genes from genera such as *Paenibacillus* and *Brevibacillus*, while 28 genes were of completely unknown taxonomic origin. Interestingly, one gene was traced from the *Tissierellia* class, specifically from *Tissierella pigra*, which is known for its role in anaerobic environments. Additionally, one gene was traced from the phylum *Pseudomonadota*, and 11 genes were marked as originating from completely unknown sources (Table 5). Fig. 7 was automatically generated by the tool as part of its standard visualization feature.

### Antibiotic resistance profile

The antibiotic susceptibility test for *L. boronitolerans* MSR1 revealed a broad resistance to 24 of the 25 tested antibiotics. Imipenem was the only antibiotic that inhibited the growth of *L. boronitolerans* MSR1, showing a clear zone of inhibition with a diameter of 23.34 ± 1.528 mm (Table 6). *Staphylococcus aureus* was used as control strain, and diameter of its inhibition zones was mostly acceptable in a wide variety of antibiotics (Table 6) (S6 Fig). This suggests that imipenem has potent bactericidal effects on this strain. The resistance pattern of *L. boronitolerans* MSR1 against multiple antibiotics, including commonly used agents like amoxicillin, ciprofloxacin, vancomycin, and tetracycline, indicates a robust resistance profile, raising concerns about treatment options if the strain were to cause infections (S2 Fig).

**Table 6:** Antibiotic susceptibility of *L. boronitolerans* MSR1.

Name of Antibiotic	*S. aureus*	*L. boronitolerans* MSR1
*Amoxy/Clav. (Augmentin) (AG, 30 µg)*	29.5 ± 0.5 mm (S)	- (R)
*Amoxycillin (AX, 10 µg)*	25 ± 1.00 mm (S)	- (R)
*Ampicillin (AMP, 25 µg)*	- (R)	- (R)
*Azithromycin (AZM, 30 µg)*	- (R)	- (R)
*Cefepime (ZX, 30 µg)*	- (R)	- (R)
*Cefixime (SF, 5 µg)*	11.4 ± 2.52 mm (S)	- (R)
*Cefotaxime (CF, 30 µg)*	9.4 ± 1.5 mm (S)	- (R)
*Ceftriaxone (RP, 30 µg)*	20.7 ± 1.5 mm (S)	- (R)
*Cefuroxime (CB, 30 µg)*	- (R)	- (R)
*Cephalexin (PR, 30 µg)*	25.5 ± 0.5 mm (S)	- (R)
*Ciprofloxacin (RC, 5 µg)*	24.4 ± 0.58 mm (S)	- (R)
*Colistin (CS, 10 µg)*	10 ± 1.00 mm (S)	- (R)
*Co-Trimoxazole (BA, 25 µg)*	30.4 ± 0.58 mm (S)	- (R)
*Doxycycline (DX, 30 µg)*	22.4 ± 0.58 mm (S)	- (R)
*Erythromycin (ER, 15 µg)*	24.4 ± 2.51 mm (S)	- (R)
*Gentamycin (GM, 10 µg)*	22 ± 1.00 mm (S)	- (R)
*Imipenem (IM, 10 µg)*	- (R)	23.34 ± 1.528 mm (S)
*Levofloxacin (QB, 5 µg)*	30 ± 2.00 mm (S)	- (R)
*Methicillin (MET, 5 µg)*	- (R)	- (R)
*Nalidixic Acid (NA, 30 µg)*	31.7 ± 1.53 mm (S)	- (R)
*Neomycin (N, 30 µg)*	20 ± 1.00 mm (S)	- (R)
*Norfloxacin (NX, 10 µg)*	23.4 ± 1.53 mm (S)	- (R)
*Streptomycin (SM, 10 µg)*	24.4 ± 0.58 mm (S)	- (R)
*Tetracycline (TE, 30 µg)*	21.4 ± 2.08 mm (S)	- (R)
*Vancomycin (VA, 30 µg)*	10.7 ± 2.09 mm (S)	- (R)

*R= Resistant, S= Susceptible.

**Fig 8:**
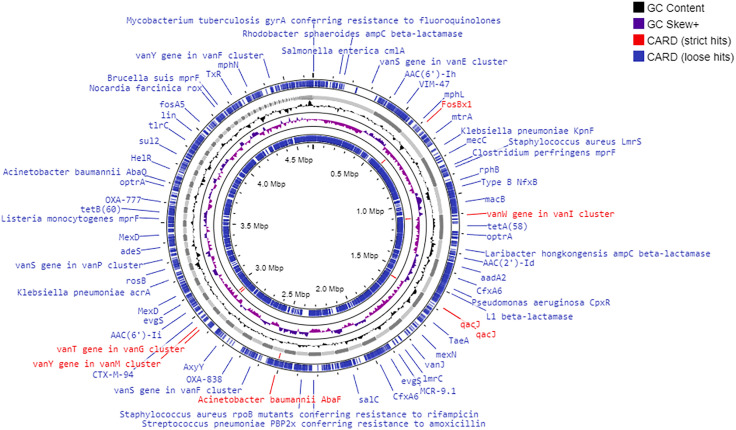
AMR gene’s position in the genome of *L. boronitolerans* MSR1. Red ones are strict hits, and blue ones are loose hits from CARD database.

*In silico* analysis using the Comprehensive Antibiotic Resistance Database (CARD) further corroborated the findings. Seven strict antibiotic resistance genes (AMR genes) were identified, alongside 312 loose hits, indicating the presence of potential resistance mechanisms encoded in the genome (Fig 8) (S4 File). The identified AMR genes, such as *qacJ*, *vanW*, *vanT*, and *FosBx1*, confer resistance to disinfectants, glycopeptides (like vancomycin), and fosfomycin through efflux pumps and target alteration mechanisms (Table 7). The identification of these genes suggests that *L. boronitolerans* MSR1 harbors both intrinsic and acquired resistance mechanisms, possibly through horizontal gene transfer (HGT). Table 8 lists several HGT events, where AMR genes have likely been incorporated into the genome through genetic exchanges with other species.

**Table 7:** Major AMR genes.

*Contigs*	*Start*	*Stop*	*ARO Term*	*AMR Gene Family*	*Drug Class*	*Antibiotic*	*Resistance Mechanism*
*NODE_10*	10974	11342	qacJ	Small multidrug resistance (SMR) antibiotic efflux pump	Disinfecting agents and antiseptics	Benzalkonium chloride	Antibiotic efflux
*NODE_10*	11356	11679	qacJ	Small multidrug resistance (SMR) antibiotic efflux pump	Disinfecting agents and antiseptics	Benzalkonium chloride	Antibiotic efflux
*NODE_6*	10053	10994	vanW gene in vanI cluster	vanW, glycopeptide resistance gene cluster	Glycopeptide antibiotic	Vancomycin; teicoplanin	Antibiotic target alteration
*NODE_28*	16064	17371	vanT gene in vanG cluster	Glycopeptide resistance gene cluster, vanT	Glycopeptide antibiotic	Vancomycin	Antibiotic target alteration
*NODE_28*	8203	9324	vanY gene in vanM cluster	vanY, glycopeptide resistance gene cluster	Glycopeptide antibiotic	Vancomycin; teicoplanin	Antibiotic target alteration
*NODE_21*	25741	26520	Acinetobacter baumannii AbaF	Major facilitator superfamily (MFS) antibiotic efflux pump	Phosphonic acid antibiotic	Fosfomycin	Antibiotic efflux
*NODE_3*	53106	53531	FosBx1	Fosfomycin thiol transferase	Phosphonic acid antibiotic	Fosfomycin	Antibiotic inactivation

**Table 8:** AMR genes from HGT event.

Contig	Start	End	HGT event detected	ORF_ID of HGT gene
Node_1	177445	178446	Same position	CPEAGCLM_00186
Node_1	184353	185015	Same position	CPEAGCLM_00193
Node_3	22650	22026	Same position	CPEAGCLM_00570
Node_3	147519	148427	Same position	CPEAGCLM_00708
Node_7	46819	47574	Same position	CPEAGCLM_01306
Node_2	43097	43888	Same position	CPEAGCLM_03069
Node_2	49556	50251	Same position	CPEAGCLM_03076
Node_3	5564	6433	Same position	CPEAGCLM_03237
Node_3	1912	2388	Same position	CPEAGCLM_03413
Node_3	38057	39796	Same position	CPEAGCLM_03494
Node_4	14128	14889	Same position	CPEAGCLM_03730
Node_5	20804	22201	Same position	CPEAGCLM_03882
Node_5	22278	23207	Same position	CPEAGCLM_03883
Node_6	1029	1781	Same position	CPEAGCLM_04198
Node_2	16064	17371	Previous gene	CPEAGCLM_02573
Node_2	53062	54060	Previous gene	CPEAGCLM_02605
Node_3	11184	11804	Next gene	CPEAGCLM_03244
Node_3	29701	30699	Next gene	CPEAGCLM_03264
Node_4	4624	5319	Next gene	CPEAGCLM_03581
Node_4	11376	14546	Next gene	CPEAGCLM_03755
Node_5	26776	27783	Next gene	CPEAGCLM_03923
Node_1	7450	8130	Next gene	CPEAGCLM_00006
Node_1	43841	44731	Next gene	CPEAGCLM_00046
Node_1	182983	184251	Next gene	CPEAGCLM_00192
Node_1	227273	228115	Previous gene	CPEAGCLM_00234
Node_2	42527	43777	Next gene	CPEAGCLM_00404
Node_4	68552	69772	Previous gene	CPEAGCLM_00817
Node_5	116609	117196	Previous gene	CPEAGCLM_01057
Node_8	58945	59592	Previous gene	CPEAGCLM_01454
Node_8	100429	101055	Next gene	CPEAGCLM_01510
Node_9	6401	7591	Next gene	CPEAGCLM_01526
Node_1	62456	63043	Previous gene	CPEAGCLM_01697
Node_1	87305	88318	Previous gene	CPEAGCLM_01722
Node_1	28309	28872	Previous gene	CPEAGCLM_02140

This extensive antibiotic resistance profile, combined with the presence of AMR genes, positions *L. boronitolerans* MSR1 as a potential multi-drug-resistant organism. While the bacterium is susceptible to imipenem, the presence of efflux pumps and glycopeptide resistance genes may contribute to future resistance development.

### Plasmid profiles and virulence attributes

Using the Plasmid MLST tool, two plasmid types were predicted within the *L. boronitolerans* MSR1 genome, A053 plasmid (Located on contig NODE_17, spanning from position 53,880–54,129, with a small difference of 173 compared to the reference database) and HCM1_043 plasmid (Found on contig NODE_2, between positions 44,815 and 45,165, with a difference of 245 from the database) (Table 9) (S5 File). These results suggest that *L. boronitolerans* MSR1 may harbor two distinct plasmids. Plasmids often carry genes related to antibiotic resistance, virulence, or environmental adaptability. However, it is notable that when using the PlasmidFinder web server, no hits for plasmids were detected, indicating a discrepancy between different prediction tools. This could be due to the specific plasmid content of *L. boronitolerans* MSR1 not matching known plasmid sequences in the PlasmidFinder database, or it might indicate that these plasmid-like elements are under-characterized.

**Table 9:** Predicted plasmid from *L. boronitolerans* MSR1 genome.

*Contig ID*	*Type of Plasmid*	*No. of Difference with database Sequence*	*Start*	*End*
*NODE_17_* *length_77106_cov_95.323735*	A053	173	53880	54129
*NODE_2_* *length_195124_cov_63.228140*	HCM1_043	245	44815	45165

The pathogenicity prediction results suggest that although the strain might have some characteristics associated with pathogenicity, there is no strong evidence to confirm its virulent nature. Notably, proteome coverage was 0.79%, meaning only a small portion of the proteome was used for this prediction. No matched pathogenic families were found which further weakens the case for pathogenicity. 30 families were found non-pathogenic, which correlates with the non-pathogenic nature of *L. boronitolerans* MSR1 (Table 10) (S6 File).

**Table 10:** Pathogenicity prediction of *L. boronitolerans* MSR1.

Probability of being a human pathogen	0.77
Input proteome coverage (%)	0.79
Matched Pathogenic Families	0
Matched Not Pathogenic Families	30

Thus, while there is a moderate probability that this strain could have pathogenic potential, the absence of matched pathogenic families strongly suggests that *L. boronitolerans* MSR1 is likely a non-pathogenic strain. Additionally, no similarity to virulence genes was predicted, reinforcing the idea that this strain does not pose a significant pathogenic threat.

### Pangenome composition and diversity

**Fig 9:**
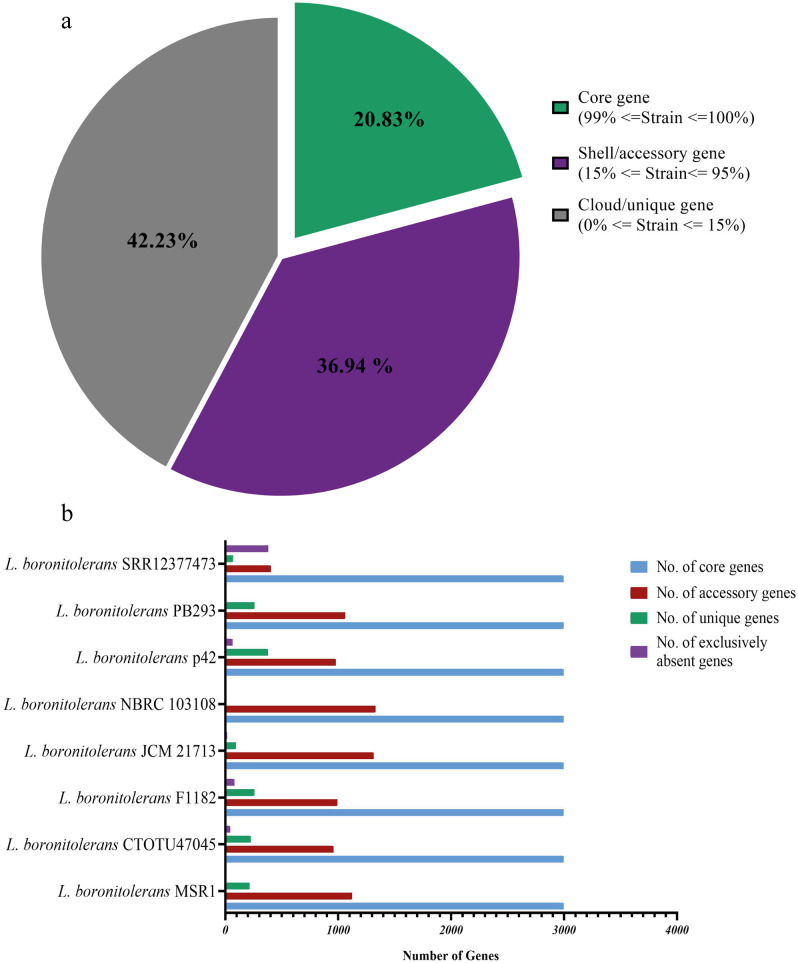
Number of genes. (a) Roary prediction of the overall core, shell and cloud gene analysis. (b) Number of core, accessory and unique genes present in individual strains.

Fig 9 presents the distribution of core, shell, and cloud genes in various strains of *L. boronitolerans*. Fig 9a shows a pie chart summarizing the results of the Roary analysis, classifying the genes into three categories. Core genes (20.83%) have a similarity of 99–100% among strains, representing the essential and conserved functions within the species. Shell genes (36.94%) similarity ranges from 15% to 95%, reflecting more strain-specific but somewhat conserved functions across the species. Cloud genes (42.23%) are highly variable and less similar than 15%, indicating genes responsible for niche adaptation, environmental response, or strain-specific traits (Table 11). Fig 9b presents a bar graph showing the distribution of core, accessory (shell), unique, and absent genes across different *L. boronitolerans* strains (Table 12). It highlights that the number of core genes is consistent across strains, representing the highly conserved portion of the genome. There is significant variation in accessory genes across strains, which contributes to functional diversity. Each strain has a small but notable set of unique genes that may give it specific traits or capabilities not shared by others. Fig 9 was generated using GraphPad Prism v8.0.2.

**Table 11:** Percentage of core, shell and cloud genes *L boronitolerans* strains.

*Type of gene*	*No. of Gene*	*Percentage*
*Core gene*	2172	20.83%
*Shell/accessory gene*	3852	36.94%
*Cloud/unique gene*	4402	42.23%

**Table 12:** Number of different genes in each *L boronitolerans* strain.

*Organism name*	*Core genes*	*Accessory genes*	*Unique genes*	*Exclusively absent genes*
*L.boronitolerans MSR1*	2996	1123	214	7
*L.boronitolerans CTOTU47045*	2996	958	226	43
*L.boronitolerans F1182*	2996	994	258	79
*L.boronitolerans JCM_21713*	2996	1314	94	14
*L.boronitolerans NBRC_103108*	2996	1331	11	0
*L.boronitolerans p42_Compleate*	2996	979	377	63
*L.boronitolerans PB293*	2996	1063	258	2
*L.boronitolerans SRR12377473*	2996	402	67	380

**Fig 10:**
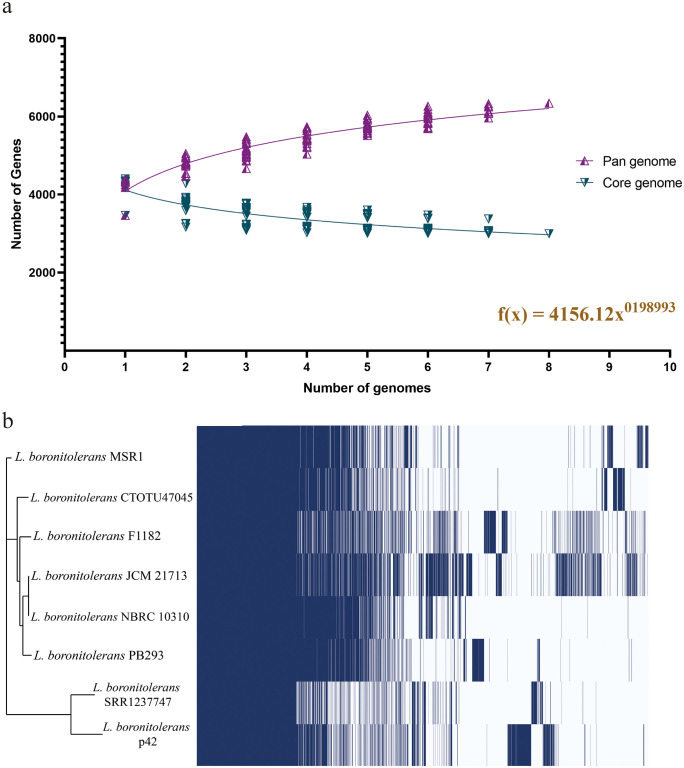
Pan genome analysis of *L. boronitolerans.* (a) Pan-plot of *L. boronitolerans* core and pan-genome. (b) Gene absence/presence matrix of *L. boronitolerans* genomes.

Fig 10a illustrates the pan-genome plot for the core and pan-genome of *L. boronitolerans* across several genomes. The purple curve represents the pan-genome (total gene repertoire across all strains), while the blue curve represents the core genome (genes shared by all strains). The regression equation shown f(x) = 4156.12x ^0.198993^ is derived from a power-law model commonly applied in pan-genome studies (S8 File). The pan-genome line is still increasing with the addition of more genomes, indicating that the genome remains “open,” meaning new genes will continue to be identified as more genomes are sequenced. However, the trend suggests that the genome is nearing saturation, meaning it could become “closed” with the inclusion of more genomes.

On the other hand, the core genome is steadily declining as more genomes are included, which reflects the fact that the shared gene pool decreases as the number of genomes increases. This indicates that while all strains of *L. boronitolerans* share a set of core genes, their genetic diversity is highlighted by the addition of accessory genes from other strains.

**Fig 11:**
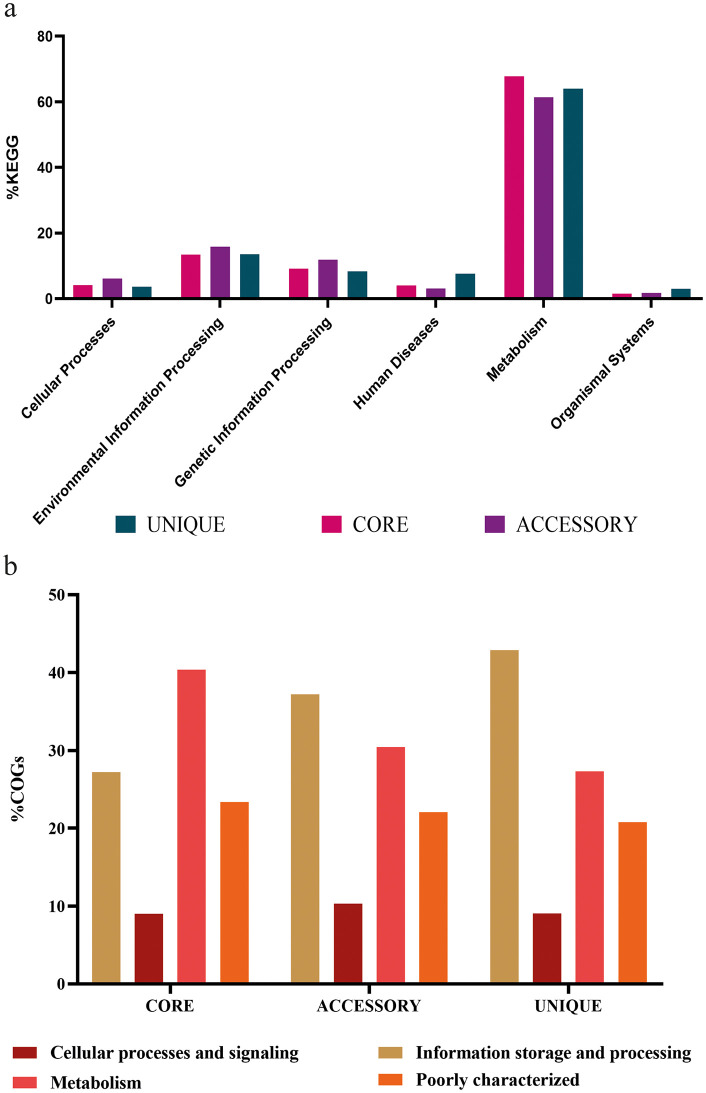
KEGG and COG distribution. (a) Major KEGG pathways of core, accessory, and unique genes. (b) Major COG distribution of core, accessory, and unique genes.

Fig 11 presents the distribution of genes across major pathways in the KEGG (Kyoto Encyclopedia of Genes and Genomes) and COG (Clusters of Orthologous Groups) databases for the core, accessory, and unique genes of *L. boronitolerans*. Core genes are predominantly associated with metabolic pathways, particularly energy production, conversion, and carbohydrate metabolism (S3 and S4 Fig). This reflects the essential nature of these genes for the basic survival and functioning of *L. boronitolerans*. Core genes also show significant involvement in other key pathways such as amino acid, lipid, and nucleotide metabolism, indicating their crucial roles in maintaining cellular processes. Accessory genes are associated with more specialized functions such as environmental information processing, signal transduction mechanisms, and cell motility (S3 and S4 Fig). This suggests that accessory genes may provide *L. boronitolerans* with the ability to adapt to different environmental conditions or stress factors by adjusting their signal processing and motility.

Unique genes are linked to more specific functions, often involving defense mechanisms, signal transduction, and genetic recombination and repair, suggesting their potential roles in strain-specific responses to environmental changes or interactions with other organisms. Notably, unique genes show minimal association with human diseases or organismal systems, which implies their role is more ecological than pathogenic (S7 File).

Fig 11a displays the COG distribution of core, accessory, and unique genes. Core genome is enriched in categories related to cellular processes and signaling, metabolism, and information storage and processing. These categories reflect fundamental cellular functions such as energy generation, biosynthesis, and transcription/translation, which are critical for the survival of all strains. The unique genes show a relatively high representation in poorly characterized functions, which might suggest novel or strain-specific functions. Unique genes are also involved in transcription, recombination, and repair, which might allow certain strains to possess enhanced genetic flexibility or environmental adaptability.

**Fig 12:**
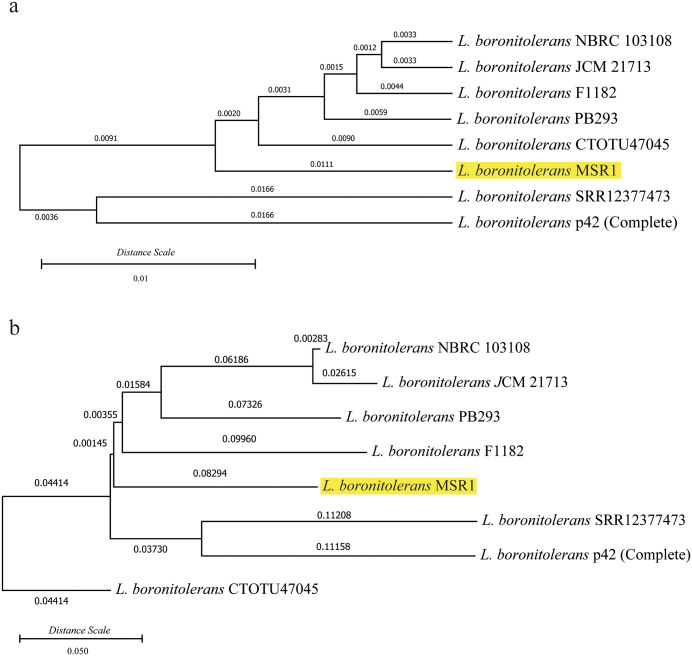
Phylogenetic relation among *L. boronitolerans* strains based on pan-genome analysis. (a) Core Phylogenetic tree of *L. boronitolerans* strains based on core gene; (b) Pan phylogenic tree based on pan-genome of *L. boronitolerans.* The digits present the branch lengths.

A phylogenetic relationship was prepared among the strains of *L. boronitolerans* based on the core genes (Fig 12) (S7 File). Fig 12 illustrates both the core and pan-genome phylogenetic relationships among the selected *L. boronitolerans* strains used in the pangenome analysis, highlighting evolutionary divergence based on shared core genes (Fig 12a) and overall gene content variability (Fig 12b), consistent with the species-level comparison following initial taxonomic identification.

## Discussion

*L. boronitolerans* MSR1 was isolated from a yogurt sample. The strain was identified through both morphological and molecular methods, including 16S rRNA sequencing, and its genomic features were further analyzed through whole-genome sequencing. The colony morphology of *L. boronitolerans* MSR1 displayed an umbonate elevation, which reflects its growth characteristics in solid media. Such morphological traits, including colony elevation and surface color, are crucial for the identification and differentiation of bacterial strains in microbiological studies [64].

The biochemical profile of *L. boronitolerans* MSR1 reveals several distinctive characteristics. Notably, the strain exhibits positive results for gram staining, catalase activity, oxidase activity, and motility, aligning with typical features of the *Lysinibacillus* genus [13]. However, MSR1 also demonstrates unique traits, such as positive reactions for the Methyl-Red and Voges-Proskauer tests, as well as indole production, which are not commonly observed in other *Lysinibacillus* species [23,65,66]. These findings suggest that MSR1 possesses a distinct metabolic profile, highlighting the phenotypic diversity within the *Lysinibacillus* genus [13,14,23,65,66].

Growth dynamics further underscore the strain’s adaptability. The growth of MSR1 was optimal at pH 8, with rapid initiation and a prolonged stationary phase, whereas lower pH levels delayed and diminished growth. This observation is supported by previous reports showing that members of the *Lysinibacillus* genus tend to thrive in environments with pH levels around 7–8, while growth is significantly reduced under more acidic conditions [13,14]. Due to the favorable growth conditions, the bacteria rapidly reached the peak of the exponential phase. These data suggest that a basic pH is critical for optimal metabolic activity and survival in these organisms. Such findings are consistent with previous studies on related *Bacillus* species, which have demonstrated enhanced growth and metabolic efficiency under alkaline conditions [10,67,68]. The decline in growth suggested that the bacteria might be entering the death phase. However, since absorbance (OD) measures total biomass, it cannot accurately confirm the death phase. Therefore, viable cell counts (CFU) would be necessary to accurately determine the death phase.

Investigating the effect of pH on growth provides insights into the strain’s adaptability and survival in acidic environments such as yogurt. Since yogurt has a low pH, understanding whether *L. boronitolerans* MSR1 can also thrive at neutral or acidic pH levels helps elucidate its ecological flexibility and potential applications. The growth curve also reveals the survival of MSR1 under acidic conditions at pH 5 and 6. Further molecular investigation is required to understand the specific mechanism or the involvement of any particular enzyme or pathway. This will provide insights into how *L. boronitolerans MSR1* thrives at higher pH levels.

Compared to other research on phylogenetic relationships within the *Lysinibacillus* genus, these findings are consistent with previous studies, which also demonstrate that whole-genome comparisons provide greater precision in resolving species boundaries and identifying genetic variations among strains [69,70]. The results of the whole-genome analysis show that *L. boronitolerans* MSR1 belongs to a genetically distinct clade within the *L. boronitolerans* species, but still shares core genomic features with the other strains.

The 16S-based phylogeny, though widely used for bacterial identification, can sometimes lead to misclassifications or insufficient resolution among closely related species due to the conserved nature of the 16S gene [71]. However, in this case, it effectively supported the classification of *L. boronitolerans* MSR1 within the *L. boronitolerans* species group, providing consistent results with the whole-genome phylogeny.

Overall, the results of this whole-genome phylogenetic analysis align with previous phylogenetic trees consistently placing *L. boronitolerans* MSR1 as closely related to *L. boronitolerans* NBRC103108. However, the branch lengths reveal that other species within the *Lysinibacillus* genus have varying degrees of genetic similarity to MSR1, with some more distantly related than others. This comprehensive analysis strengthens the identification of MSR1 as a strain of *L. boronitolerans*.

The findings of this study highlight *L. boronitolerans* MSR1’s broad resistance to antibiotics, a pattern consistent with other studies on *Lysinibacillus* species. Similar studies on *Lysinibacillus fusiformis* and *Lysinibacillus sphaericus* have shown resistance to various antibiotics, particularly to glycopeptides, β-lactams, and aminoglycosides, which suggests that members of the *Lysinibacillus* genus possess intrinsic or acquired resistance mechanisms [72,73]. The reliability of the susceptibility testing was ensured by the inclusion of *S. aureus* as a reference strain. Further studies are needed to confirm the antibiotic susceptibility results using more control strains with known resistance profiles.

The resistance observed in *L. boronitolerans* MSR1 is particularly concerning due to the presence of vancomycin resistance genes (*vanW* and *vanT*), which have been commonly reported in clinically relevant, multi-drug-resistant organisms [74]. Vancomycin-resistant strains pose significant challenges in clinical treatment, as they are often associated with hospital-acquired infections and biofilm formation [75].

However, the susceptibility to imipenem is a positive finding, suggesting that carbapenems may be an effective therapeutic option for infections caused by this strain. In comparison, carbapenem resistance has been increasingly reported in other Gram-positive bacteria, but in this case, *L. boronitolerans* MSR1 remains vulnerable to imipenem [76]. Although data on carbapenem susceptibility in the *Lysinibacillus* genus is limited, studies show that species like *L. sphaericus* are susceptible to carbapenems [18]. This matches our findings for *L. boronitolerans* MSR1, suggesting a similar pattern across the genus.

The identification of genes likely acquired through HGT (Table 8) aligns with previous research indicating that horizontal gene transfer is a significant driver of antibiotic resistance in bacteria [77]. The integration of AMR genes into the genome suggests that *L. boronitolerans* MSR1 has the potential to adapt to diverse environments, including those with high antibiotic pressure. This has implications for understanding the evolutionary trajectory of resistance in environmental and clinically significant strains [78].

The efflux pump genes of small multidrug resistance (SMR) (qacJ) are of special interest, as they confer resistance to disinfectants and antiseptics, which makes us worried about the possible occurrence of survival of this strain in hospital or industrial environments [79]. Efflux pumps expel toxic compounds, including antibiotics and disinfectants, from bacterial cells, contributing to resistance [80]. Their impact varies among bacterial species and environmental conditions. For example, in *Acinetobacter baumannii*, the AmvA efflux pump enhances tolerance to long-chain polyamines, which may have evolved as a defense against disinfectants [81]. Similarly, the AdeABC efflux pump further strengthens resistance by reducing intracellular antibiotic accumulation [81,82]. These pumps are significantly involved in multidrug resistance which enables the bacteria to endure unfavorable environments which in this case include hospitals where disinfectants and antibiotics are much more in use [80–82]. A more in-depth study is required to determine the exact role of efflux pumps in *L. boronitolerans* MSR1’s persistence in different environments.

In comparison with other studies, such as those on *Bacillus subtilis* and *Bacillus cereus*, which also exhibit high levels of resistance to various antibiotics, *L. boronitolerans* MSR1’s resistance profile appears to be broader [83]. The presence of vancomycin resistance genes and efflux pumps in *L. boronitolerans* MSR1 suggests that this strain may share similar resistance mechanisms with clinically relevant *Enterococcus* and *Staphylococcus* species, which have long been studied for their ability to withstand [84–86].

Additionally, the detection of HGT events reinforces the idea that gene flow among bacterial species plays a significant role in the dissemination of resistance traits across different bacterial populations [78]. Further *in-vitro* analyses are required to understand the precise mechanism of resistance gene acquisition. Some AMR genes were found to be associated with HGT, which may serve as a key pathway for acquiring resistance genes [53,87–89].

The high resistance of *L. boronitolerans* MSR1 to antibiotics brings up the issue of reservoirs of AMR genes in the environment since the strain could serve as a channel through which resistance is transferred to microbial communities. Hence, it is important to monitor and control the use of antibiotics in agriculture and industry so that it can reduce the selection pressure that promotes the development of the resistant strains. Strict control of antibiotics, improved genetic surveillance, alternative antimicrobials, better hygiene, waste water management system, and educating people is necessary to contain the threat of AMR in food-associated bacteria, such as *L. boronitolerans* MSR1 [90–93]. These strategies help reduce bacterial contamination and limit the spread of resistance genes in the food supply chain.

Both genome-wide and 16S rRNA-mediated phylogenetic studies offer an overarching map of the evolutionary position of the *L. boronitolerans* MSR1 in *Lysinibacillus* genus. The whole-genome phylogeny provides a more comprehensive and accurate perspective on the relationships among strains because it uses the entire genetic material of the organism in comparison to the phylogeny based on 16S rRNA, which only uses a small part of the genome [71]. This is reflected in the stronger resolution of closely related strains, such as the grouping of *L. boronitolerans* MSR1 with *L. boronitolerans* NBRC103108. Phylogenetic tree analysis aligns well with the previous ANI comparison, which confirmed that *L. boronitolerans* MSR1 is closely related to *L. boronitolerans* NBRC103108 with high genomic similarity.

The identification of MNPs, which involve the substitution of two or more adjacent nucleotides, as well as the detection of indels (insertions and deletions), indicates that a range of mutational mechanisms is shaping the genetic landscape of *L. boronitolerans* MSR1 [94]. The presence of these genetic variations could have important implications for the functional genomics of the strain, potentially affecting gene expression, protein function, and overall phenotype. These mutations may also play a role in strain-specific adaptations, possibly influencing the strain’s metabolic capacities, stress tolerance, or ecological niche [95].

The diversity and types of BGCs of *L. boronitolerans* MSR1 are similar to other researches in which Bacillus and Lysinibacillus species are known to produce a great variety of secondary metabolites, such as antibiotics, toxins, and enzymes [96,97]. However, the lower similarity percentages in some clusters, particularly the NRPS-like and lanthipeptide regions, indicate that *L. boronitolerans* MSR1 may possess unique biosynthetic capabilities. These unique BGCs could be explored further for their potential in biotechnology and drug discovery applications.

In comparison with other bacterial genomes, the number of HGTs identified in *L. boronitolerans* MSR1 is significant, as it suggests a high degree of gene acquisition from diverse species. Most of the HGT events are within the same phylum (*Bacillota*), indicating that gene transfer events may occur frequently between closely related species, likely due to shared ecological niches [98]. The detection of genes from the phyla *Pseudomonadota* and unknown sources also highlights the possibility of more distant or novel horizontal gene transfer events, suggesting potential adaptation strategies of *L. boronitolerans* in various environmental conditions [99].

The predominance of *Bacillales*-related HGT genes reflects the close evolutionary relationship between these species and their potential for genetic exchange. Other studies on related *Bacillus* and *Lysinibacillus* species have also reported similar trends, where gene transfer within the same phylum or class is more common due to shared evolutionary histories [98]. However, the presence of genes from distant taxa such as *Pseudomonadota* may reflect unique environmental pressures or the presence of these organisms in diverse ecological settings [78]. The high number of unknown HGT sources in *L. boronitolerans* MSR1 indicates the need for further genomic exploration to uncover potentially novel interactions or undiscovered microbial species contributing to its genome.

When comparing these findings to other *Lysinibacillus* species, many strains are known for their environmental robustness and diverse applications rather than pathogenicity [14–17,70,96,100]. Plasmids in *Lysinibacillus* species often contribute to ecological adaptability, such as heavy metal resistance, rather than virulence [70]. Similar results are observed in *Bacillus* species, where environmental plasmids are more common than those carrying pathogenic genes [101–103].

Moreover, other studies on related species often report mixed results regarding plasmid detection depending on the tool used [104]. The lack of hits from the PlasmidFinder database contrasts with the detection from the MLST tool, which has been observed in other bacterial genome analyses [105]. Both PlasmidFinder and MLST employ different algorithms and databases, which may lead to variations in the results [59]. While PlasmidFinder identifies plasmids based on known replicon sequences, MLST classifies bacterial strains based on sequence types, which may not always align with plasmid data [43,59]. This discrepancy highlights the need for comprehensive plasmid identification pipelines to capture well-characterized and novel plasmid elements.

In terms of pathogenic potential, non-pathogenic species may possess moderate predictions of pathogenicity without strong evidence of actual virulence. The results here are consistent with such findings, suggesting that while the strain has some potential features, it is more likely to be benign or beneficial in non-host environments.

The BGC profile of MSR1 appears to be unique in terms of the presence of both RiPP-like and betalactone BGCs. Other *Lysinibacillus* strains have been predominantly studied for their potential in bioremediation, but the discovery of antimicrobial BGCs in MSR1 opens new avenues for exploring its role in natural microbial competition and secondary metabolite production [14].

*L. boronitolerans* MSR1 also harbors terpene biosynthetic genes. Terpenes are known for their roles in flavor and aroma production in various fermented foods. For instance, terpenes like limonene and myrcene contribute to citrus and herbal notes, respectively, in foods and beverages [106]. In the context of yogurt production, terpenes could potentially influence flavor profiles. Studies have shown that certain bacterial strains involved in yogurt fermentation, such as *Lactobacillus* species, can enhance yogurt flavor through the production of volatile compounds, including terpenes and esters [107]. While *L. boronitolerans* MSR1 is not typically associated with yogurt production, the terpene BGC in this strain may indicate a similar potential for flavor contribution, although further studies are required to validate this hypothesis. This would necessitate functional studies to determine whether these terpene products significantly contribute to yogurt flavor profiles compared to strains traditionally used in fermentation. Further studies would be necessary to determine the precise mechanism and role in flavor enhancement.

The identification of RiPP-like and lanthipeptide BGCs in *L. boronitolerans* MSR1 suggests it could potentially be explored for probiotic applications or natural preservation in the food industry [108]. RiPPs play a crucial role in medicine, agriculture, and food preservation [109]. They are also investigated as new medicines, biopesticides, and natural preservatives, which can help to make agriculture and food safer. RiPP-based drugs are in clinical trials or FDA-approved with many drugs demonstrating pharmaceutical potential [109–111]. Lanthipeptides exhibit diverse bioactivities, including antimicrobial, antiviral and immunomodulatory properties, making them promising candidates for novel therapeutics [112]. Their potential in combating antibiotic resistance and treating diseases like cystic fibrosis highlights their significance in modern drug discovery [112–115]. T3PKS-type polyketides and NRPS-type NAD4 indicate the possibility of the strain to generate bioactive molecules to be used in pharmaceutical purposes, including antibiotics or anticancer molecules [116]. Additionally, these biosynthetic pathways could have agricultural applications, including the development of natural herbicides or pesticides [116,117]. The strain’s ability to produce these metabolites highlights its value for biotechnology and the discovery of novel, sustainable solutions for medicine and agriculture.

Compared to other pan-genome analyses, e.g., those carried out in *Bacillus subtilis* and other *Lysinibacillus* species, the proportion of core genes, shell genes, and cloud genes in *L. boronitolerans* MSR1 is in line with the proportion of these genes in many bacterial species, especially those that live in the environment or on soil. It is found that such species contain more cloud genes because they are capable of adapting to extremely changing environments [27,118,119].

This has been demonstrated by similar studies of Bacillus species, which have a similar proportion of core versus accessory genes, with core genes being essential metabolic and housekeeping functions and the accessory or cloud genes being associated with ecological versatility, antibiotic resistance or association with host organisms [118]. The high percentage of cloud genes (42.23%) in *L. boronitolerans* suggests that this species might be highly adaptable to diverse environments, possibly enabling it to survive in various niches or under changing environmental conditions [120].

The presence of a large proportion of shell and cloud genes reflects a high level of genetic diversity within *L. boronitolerans* strains. This is typical for bacteria inhabiting varied environments, where selective pressure may drive horizontal gene transfer, gene loss, or other genetic mechanisms that introduce variability [121]. The core genome, which consists of 2172 genes, likely represents the essential functionalities needed for cellular processes like metabolism, replication, and basic survival [120].

The variation in accessory and unique genes across strains suggests that while all strains share a core set of genetic tools, the accessory genome enables certain strains to specialize. These unique or accessory genes might confer advantages like the ability to metabolize different substrates, withstand specific environmental stresses, or interact with other microorganisms or hosts [122].

Interestingly, BPGA prediction of 2996 core genes has a higher estimation as compared to Roary which is 2172. The difference can be attributed to the differences in the algorithmic methodologies of the two tools and the fact that defining core genomes is complicated and that the threshold of gene prediction determines how core genomes are identified..

BPGA utilizes USEARCH with a 50% identity cutoff, allowing for more lenient clustering of homologous genes [56]. In contrast, Roary applies a stricter default BLASTP percentage identity threshold of 95% for gene clustering, resulting in a more conservative estimation of core genes [57]. This variation underscores the impact of clustering parameters on pangenome analyses and highlights the importance of considering algorithmic differences when interpreting results. This comprehensive approach ensured a robust and accurate pangenome characterization, reinforcing the genetic diversity within the *Lysinibacillus* genus.

The finding of an “open” pan-genome for *L. boronitolerans* is consistent with other bacterial species, particularly those inhabiting diverse and dynamic environments [121]. For instance, similar trends have been reported in soil-dwelling species like *Bacillus subtilis*, which also exhibits an open pan-genome, reflecting its ability to acquire genes via horizontal gene transfer to adapt to changing environmental conditions [118].

In contrast, obligate pathogens or host-associated bacteria tend to have closed pan-genomes due to their specialized niches and reduced gene acquisition [123]. The fact that *L. boronitolerans* shows a trend towards closing pan-genome suggests that while it continues to accumulate new genes, its gene repertoire may soon stabilize. This trend is often seen in bacteria that, while versatile, may inhabit relatively defined ecological niches, thereby limiting the extent of gene acquisition over time [124]. Additionally, the high diversity in accessory genes, as highlighted by the presence/absence matrix, indicates substantial functional diversity among *L. boronitolerans* strains, which could be related to differences in habitat, resource utilization, or environmental stress adaptation [121,125].

Further research should focus on expanding the genomic dataset by sequencing additional strains from diverse environments. Comparative genomic studies could provide deeper insights into strain-specific adaptations, gene acquisitions, and evolutionary trends [122]. Additionally, functional validation of newly acquired genes, particularly those associated with antimicrobial resistance (AMR) and stress tolerance, is essential to understanding their role in the bacterium’s adaptability [121,122,126,127]

The KEGG and COG functional distributions of *L. boronitolerans* give important clues to the genomic structure of the bacterium and its ecological functions that the bacteria may have [128]. The core, accessory and unique gene functional analysis indicates that although *L. boronitolerans* has a set of vital genes needed to underline the normal cellular functioning, it becomes more adaptive and versatile due to the changeable availability of accessory and unique genes that enable environmental reaction and extinction. This is common to bacterial species that inhabit different ecological habitats and indicates the capacity of the organism to survive in variable environments [121,129,130].

The assembly level of the genome of *L. boronitolerans* MSR1 is a draft assembly rather than a complete genome. As with any draft assembly, limitations include potential gaps in the genome, fragmented contigs, and unresolved repetitive regions, which may impact the completeness of certain genomic analyses [131]. As the isolate is newly identified, default parameters were used in multiple software tools to ensure broad and unbiased results, allowing comprehensive analysis without imposing restrictive criteria.

The isolation of a non-pathogenic but multi-drug-resistant bacterium from yogurt (*L. boronitolerans* MSR1), highlights a complex issue in food microbiology. While the bacterium itself does not pose a direct threat to human health due to its non-pathogenic nature, its multi-drug resistance profile presents concerns regarding the potential dissemination of AMR genes through the food chain [92]. These bacteria may serve as reservoirs for antimicrobial resistance (AMR) genes, which can be transferred to pathogenic bacteria via horizontal gene transfer (HGT), potentially leading to drug-resistant infections [88]. Further research is needed to understand the implications of this resistance in food-related environments. To mitigate this risk, strict surveillance, monitoring, and regulations on antibiotic use in food production are essential. As MSR1 was predicted to be non-pathogenic based on in silico analysis, future research could verify this through experimental approaches such as in vitro cytotoxicity assays, in vivo infection models, or evaluation of virulence-associated phenotypes to confirm its safety for potential applications.

In addition to its unique presence in yogurt, *L. boronitolerans* MSR1 exhibits distinct physiological and genomic traits that differentiate it from other strains of the species. Genomic analysis revealed several potential biosynthetic gene clusters, which could offer insight into its adaptive mechanisms and potential applications in biotechnology and food safety.

## Conclusion

The isolation and characterization of *Lysinibacillus boronitolerans* MSR1 from yogurt provide valuable insights into the genomic, phenotypic, and functional attributes of this bacterium. While MSR1 is non-pathogenic, its multi-drug resistance profile, particularly against glycopeptides, aminoglycosides and fosfomycin raises concerns about the potential spread of antimicrobial resistance (AMR) genes in food-related environments. The presence of resistance genes such as *qacJ, vanW,* and *FosBx1* points to the bacterium’s ecological adaptation and potential for horizontal gene transfer. Additionally, genomic analyses revealed unique regions and a diverse array of biosynthetic gene clusters (BGCs), including lanthipeptides, terpenes, and RiPPs, which may contribute to the bacterium’s survival in its niche and could be explored for their potential roles in yogurt flavor enhancement. These findings underscore the importance of continued research into the safety, functional capabilities, and ecological roles of non-pathogenic, multi-drug-resistant bacteria in food systems. Ultimately, a deeper understanding of *L. boronitolerans* MSR1 could advance its use in industrial applications while ensuring it does not pose a risk to public health through the spread of AMR.

## Supporting information

S1 Fig Morphological and biochemical characteristics of *L. boronitolerans* MSR1. (PNG)

S2 Fig Antibiotic susceptibility test of *L. boronitolerans* MSR1. (PNG)

S3 Fig KEGG bar plot from BPGA analysis of *L. boronitolerans* MSR1. (PNG)

S4 Fig COG bar plot from BPGA analysis of *L. boronitolerans* MSR1. (PNG)

S5 Fig 16s gene PCR amplification. Gel documentation image of *L. boronitolerans* MSR1.
(PNG)

S6 Fig Antibiotic susceptibility test of *Staphylococcus aureus.* (PNG)

S1 File Growth curve data and retrieved genomes list from NCBI. S_Table 1: Absorbance of *L. boronitolerans* MSR1 growth in different pH. S_Table 2: Genomes of *L. boronitolerans* retrieved from NCBI Genome Browser.
(DOCX)

S2 File SNP analysis of *L. boronitolerans* MSR1. (CSV)

S3 File HGT analysis result of *L. boronitolerans* MSR1. (XLSX)

S4 File Predicted AMR genes in *L. boronitolerans* MSR1. (XLSX)

S5 File Predicted plasmid sequences of *L. boronitolerans* MSR1. (DOCX)

S6 File Virulence property of *L. boronitolerans* MSR1. (PDF)

S7 File COG and KEGG distribution from BPGA of *L. boronitolerans.* (ZIP)

S8 File Pag-genome results from BPGA of *L. boronitolerans.* (ZIP)

S9 File Pan-genome results from Roarry of *L. boronitolerans.* (ZIP)

S10 File ANI matrix of *L. boronitolerans.* (XLSX)

## References

[1] Fernandez MA, Fisberg M, Marette A. Role of yogurt in the nutrition and health of children and adolescents. Yogurt Health Disease Prevent. Elsevier. 2017. p. 491–505.

[2] Sumi K, et al. Nutritional value of yogurt as a protein source: digestibility/absorbability and effects on skeletal muscle. Nutrients. 2023;15(20). 10.3390/nu15204366 PMC10609537 37892442

[3] Moore JB, Horti A, Fielding BA. Evaluation of the nutrient content of yogurts: a comprehensive survey of yogurt products in the major UK supermarkets. BMJ Open. 2018;8(8):e021387. doi: 10.1136/bmjopen-2017-021387 30228100 PMC6144340

[4] Sudheer A, Dastidar DG, Ghosh G, Taj Z, Nidhin IK, Chattopadhyay I. Comprehensive genomics, probiotic, and antibiofilm potential analysis of Streptococcus thermophilus strains isolated from homemade and commercial dahi. Sci Rep. 2025;15(1):7089. doi: 10.1038/s41598-025-90999-w 40016393 PMC11868508

[5] Sfakianakis P, Tzia C. Conventional and innovative processing of milk for yogurt manufacture; development of texture and flavor: a review. Foods. 2014;3(1):176–93. doi: 10.3390/foods3010176 28234312 PMC5302305

[6] Jakaria Al-Mujahidy SM, Kryukov K, Ikeo K, Saito K, Uddin ME, Ibn Sina AA. Functional genomic analysis of the isolated potential probiotic Lactobacillus delbrueckii subsp. indicus TY-11 and its comparison with other Lactobacillus delbrueckii strains. Microbiol Spectr. 2024;12(7):e0347023. doi: 10.1128/spectrum.03470-23 38771133 PMC11218508

[7] Zheng X, Liang Q, Zhao B, Song X, Zhang Y. Whole genome sequencing and analysis of probiotic characteristics for Lactiplantibacillus plantarum EL2 isolated from yak yogurt. LWT. 2024;198:116039. doi: 10.1016/j.lwt.2024.116039

[8] El-Sayed AS, Ibrahim H, Farag MA. Detection of Potential Microbial Contaminants and Their Toxins in Fermented Dairy Products: a Comprehensive Review. Food Anal Methods. 2022;15(7):1880–98. doi: 10.1007/s12161-022-02253-y

[9] Aziz G, Zaidi A, Bakht U, Parveen N, Ahmed I, Haider Z, et al. Microbial safety and probiotic potential of packaged yogurt products in Pakistan. Journal of Food Safety. 2019;40(1). doi: 10.1111/jfs.12741

[10] Tirloni E, et al. Bacillus cereus in dairy products and production plants. Foods. 2022;11(17). 10.3390/foods11172572 PMC9455733 36076758

[11] Haydushka IA, Markova N, Kirina V, Atanassova M. Recurrent sepsis due to bacillus licheniformis. J Glob Infect Dis. 2012;4(1):82–3. doi: 10.4103/0974-777X.93768 22529634 PMC3326966

[12] Nam Y-D, Seo M-J, Lim S-I, Lee S-Y. Genome sequence of Lysinibacillus boronitolerans F1182, isolated from a traditional Korean fermented soybean product. J Bacteriol. 2012;194(21):5988. doi: 10.1128/JB.01485-12 23045499 PMC3486081

[13] Ahmed I, Yokota A, Yamazoe A, Fujiwara T. Proposal of Lysinibacillus boronitolerans gen. nov. sp. nov., and transfer of Bacillus fusiformis to Lysinibacillus fusiformis comb. nov. and Bacillus sphaericus to Lysinibacillus sphaericus comb. nov. Int J Syst Evol Microbiol. 2007;57(Pt 5):1117–25. doi: 10.1099/ijs.0.63867-0 17473269

[14] Jamal QMS, Ahmad L. Lysinibacilli: A biological factories intended for bio-insecticidal, bio-control, and bioremediation activities. J Fungi (Basel). 2022;8(12). 10.3390/jof8121288 PMC9783698 36547621

[15] Xu J-M, Lu C, Wang W-J, Du Z-Y, Pan J-J, Cheng F, et al. Strain Screening and Particle Formation: a Lysinibacillus boronitolerans for Self-Healing Concrete. Appl Environ Microbiol. 2022;88(18):e0080422. doi: 10.1128/aem.00804-22 36036598 PMC9499011

[16] Bhatia D, Mittal A, Malik DK. Antimicrobial potential and in vitro cytotoxicity study of polyvinyl pyrollidone-stabilised silver nanoparticles synthesised from Lysinibacillus boronitolerans. IET Nanobiotechnol. 2021;15(4):427–40. doi: 10.1049/nbt2.12054 34694715 PMC8675779

[17] Tang Y, Lei J, Ma X, Li J, Li H, Liu Z. Identification and characterization of a novel bacteriocin gene cluster in Lysinibacillus boronitolerans. Biotechnol Appl Biochem. 2023;70(6):1860–9. doi: 10.1002/bab.2488 37431158

[18] Torres JL, Faulhaber JR. Triple-valve endocarditis due to Lysinibacillus sphaericus infection. IDCases. 2023;33:e01856. doi: 10.1016/j.idcr.2023.e01856 37577048 PMC10415700

[19] Malki EL, Belaouni M, Lahmadi K, Souly K, Zouhdi M. A rare case of corneal abscess caused by Lysinibacillus sphaericus. Saudi Journal of Pathology and Microbiology. 2024;9(5):104–5.

[20] Meng Z, Duan R, Lv D, Bu G, Gao Y, Zhang P, et al. Rare case of bacteremia due to Lysinibacillus sphaericus in a person living with HIV. Int J Infect Dis. 2023;135:91–4. doi: 10.1016/j.ijid.2023.08.013 37595679

[21] Wenzler E, Kamboj K, Balada-Llasat J-M. Severe Sepsis Secondary to Persistent Lysinibacillus sphaericus, Lysinibacillus fusiformis and Paenibacillus amylolyticus Bacteremia. Int J Infect Dis. 2015;35:93–5. doi: 10.1016/j.ijid.2015.04.016 25931198

[22] Pantoja-Guerra M, Burkett-Cadena M, Cadena J, Dunlap CA, Ramírez CA. Lysinibacillus spp.: an IAA-producing endospore forming-bacteria that promotes plant growth. Antonie Van Leeuwenhoek. 2023;116(7):615–30. doi: 10.1007/s10482-023-01828-x 37138159 PMC10257616

[23] Akintayo SO, et al. Lysinibacillus irui sp. nov., isolated from Iru, fermented African locust beans. Int J Syst Evol Microbiol. 2023;73(11). 10.1099/ijsem.0.006167 37943169

[24] Rahman MS, Shimul MEK, Parvez MAK. Comprehensive analysis of genomic variation, pan-genome and biosynthetic potential of Corynebacterium glutamicum strains. PLoS One. 2024;19(5):e0299588. doi: 10.1371/journal.pone.0299588 38718091 PMC11078359

[25] Tran PN, Yen M-R, Chiang C-Y, Lin H-C, Chen P-Y. Detecting and prioritizing biosynthetic gene clusters for bioactive compounds in bacteria and fungi. Appl Microbiol Biotechnol. 2019;103(8):3277–87. doi: 10.1007/s00253-019-09708-z 30859257 PMC6449301

[26] Alam K, Islam MM, Gong K, Abbasi MN, Li R, Zhang Y, et al. In silico genome mining of potential novel biosynthetic gene clusters for drug discovery from Burkholderia bacteria. Comput Biol Med. 2022;140:105046. doi: 10.1016/j.compbiomed.2021.105046 34864585

[27] Wu X, et al. Whole genome sequencing and comparative genomic analyses of Lysinibacillus pakistanensis LZH-9, a halotolerant strain with excellent COD removal capability. Microorganisms. 2020;8(5). 10.3390/microorganisms8050716 PMC7284689 32408484

[28] Gu X, Li X, Zhang R, Zheng R, Li M, Huang R, et al. Isolation and characterization of a novel highly efficient bacterium Lysinibacillus boronitolerans QD4 for quantum dot biosynthesis. Front Microbiol. 2025;16:1521632. doi: 10.3389/fmicb.2025.1521632 39944647 PMC11814218

[29] Rahman MdS, Emon DD, Nupur AH, Mazumder MdAR, Iqbal A, Alim MdA. Isolation and characterization of probiotic lactic acid bacteria from local yogurt and development of inulin-based synbiotic yogurt with the isolated bacteria. Appl Food Res. 2024;4(2):100457. doi: 10.1016/j.afres.2024.100457

[30] Fratesi SE, Lynch FL, Kirkland BL, Brown LR. Effects of SEM Preparation Techniques on the Appearance of Bacteria and Biofilms in the Carter Sandstone. J Sedimentary Res. 2004;74(6):858–67. doi: 10.1306/042604740858

[31] Lyne PM, Grange JM. Collins and Lyne’s microbiological methods. 1995: Butterworth-Heinemann.

[32] Rahman MS, Mohammad Abu Hena Mostofa Jamal, Biswas PK, Rahman SM, Sharma SP, Saha SK, et al. Arsenic remediation in Bangladeshi rice varieties with enhance plant growth by unique arsenic-resistant bacterial isolates. Geomicrobiol J. 2019;37(2):130–42.

[33] Jerin I. Diesel degradation efficiency of Enterobacter sp., Acinetobacter sp., and Cedecea sp. isolated from petroleum waste dumping site: a bioremediation view point. Archiv Microbiol. 2021;203(8):5075–84. 10.1007/s00203-021-02469-2 34302508

[34] Ekram MA-E, Sarker I, Rahi MS, Rahman MA, Saha AK, Reza MA. Efficacy of soil-borne Enterobacter sp. for carbofuran degradation: HPLC quantitation of degradation rate. J Basic Microbiol. 2020;60(5):390–9. doi: 10.1002/jobm.201900570 32115726

[35] Lonsway DR. Preparation of Routine Media and Reagents Used in Antimicrobial Susceptibility Testing. 5th ed. American Society of Microbiology. 2023.

[36] Institute C a LS. Performance standards for antimicrobial susceptibility testing. 30 ed. Wayne, PA: Clinical and Laboratory Standards Institute. 2020.

[37] Frank JA, Reich CI, Sharma S, Weisbaum JS, Wilson BA, Olsen GJ. Critical evaluation of two primers commonly used for amplification of bacterial 16S rRNA genes. Appl Environ Microbiol. 2008;74(8):2461–70. doi: 10.1128/AEM.02272-07 18296538 PMC2293150

[38] Versmessen N, Van Simaey L, Negash AA, Vandekerckhove M, Hulpiau P, Vaneechoutte M, et al. Comparison of DeNovix, NanoDrop and Qubit for DNA quantification and impurity detection of bacterial DNA extracts. PLoS One. 2024;19(6):e0305650. doi: 10.1371/journal.pone.0305650 38885212 PMC11182499

[39] Andrews S. FastQC: a quality control tool for high throughput sequence data. Babraham Bioinformatics. 2010.

[40] Joshi NA, F J. Sickle: A sliding-window, adaptive, quality-based trimming tool for FastQ files. 2011.

[41] Prjibelski A, et al. Using SPAdes de novo assembler. Curr Protoc Bioinformatics. 2020;70(1):e102. 10.1002/cpbi.102 32559359

[42] Walker BJ, Abeel T, Shea T, Priest M, Abouelliel A, Sakthikumar S, et al. Pilon: an integrated tool for comprehensive microbial variant detection and genome assembly improvement. PLoS One. 2014;9(11):e112963. doi: 10.1371/journal.pone.0112963 25409509 PMC4237348

[43] Jolley KA, Bray JE, Maiden MCJ. Open-access bacterial population genomics: BIGSdb software, the PubMLST.org website and their applications. Wellcome Open Res. 2018;3:124. doi: 10.12688/wellcomeopenres.14826.1 30345391 PMC6192448

[44] Meier-Kolthoff JP, Göker M. TYGS is an automated high-throughput platform for state-of-the-art genome-based taxonomy. Nat Commun. 2019;10(1):2182. doi: 10.1038/s41467-019-10210-3 31097708 PMC6522516

[45] Seemann T. Prokka: rapid prokaryotic genome annotation. Bioinformatics. 2014;30(14):2068–9. doi: 10.1093/bioinformatics/btu153 24642063

[46] Grant JR, Enns E, Marinier E, Mandal A, Herman EK, Chen C-Y, et al. Proksee: in-depth characterization and visualization of bacterial genomes. Nucleic Acids Res. 2023;51(W1):W484–92. doi: 10.1093/nar/gkad326 37140037 PMC10320063

[47] Alikhan N-F, Petty NK, Ben Zakour NL, Beatson SA. BLAST ring image generator (BRIG): simple prokaryote genome comparisons. BMC Genomics. 2011;12:402. doi: 10.1186/1471-2164-12-402 21824423 PMC3163573

[48] Lee I, Ouk Kim Y, Park S-C, Chun J. OrthoANI: An improved algorithm and software for calculating average nucleotide identity. Int J Syst Evol Microbiol. 2016;66(2):1100–3. doi: 10.1099/ijsem.0.000760 26585518

[49] Chen C, Wu Y, Li J, Wang X, Zeng Z, Xu J, et al. TBtools-II: A “one for all, all for one” bioinformatics platform for biological big-data mining. Mol Plant. 2023;16(11):1733–42. doi: 10.1016/j.molp.2023.09.010 37740491

[50] TS. Snippy: fast bacterial variant calling from NGS reads. 2015.

[51] Blin K, et al. antiSMASH 7.0: new and improved predictions for detection, regulation, chemical structures and visualisation. Nucleic Acids Res. 2023;51(W1): p. W46–50. 10.1093/nar/gkad344 PMC10320115 37140036

[52] Zhu Q, Kosoy M, Dittmar K. HGTector: an automated method facilitating genome-wide discovery of putative horizontal gene transfers. BMC Genomics. 2014;15(1):717. doi: 10.1186/1471-2164-15-717 25159222 PMC4155097

[53] Liu G, Thomsen LE, Olsen JE. Antimicrobial-induced horizontal transfer of antimicrobial resistance genes in bacteria: a mini-review. J Antimicrob Chemother. 2022;77(3):556–67. doi: 10.1093/jac/dkab450 34894259

[54] Alcock BP, et al. CARD 2023: expanded curation, support for machine learning, and resistome prediction at the Comprehensive Antibiotic Resistance Database. Nucleic Acids Res. 2023;51(D1): p. D690–9. 10.1093/nar/gkac920 PMC9825576 36263822

[55] Rajput A, Chauhan SM, Mohite OS, Hyun JC, Ardalani O, Jahn LJ, et al. Pangenome analysis reveals the genetic basis for taxonomic classification of the Lactobacillaceae family. Food Microbiol. 2023;115:104334. doi: 10.1016/j.fm.2023.104334 37567624

[56] Chaudhari NM, Gupta VK, Dutta C. BPGA- an ultra-fast pan-genome analysis pipeline. Sci Rep. 2016;6:24373. doi: 10.1038/srep24373 27071527 PMC4829868

[57] Page AJ, Cummins CA, Hunt M, Wong VK, Reuter S, Holden MTG, et al. Roary: rapid large-scale prokaryote pan genome analysis. Bioinformatics. 2015;31(22):3691–3. doi: 10.1093/bioinformatics/btv421 26198102 PMC4817141

[58] Edgar RC. Search and clustering orders of magnitude faster than BLAST. Bioinformatics. 2010;26(19):2460–1. doi: 10.1093/bioinformatics/btq461 20709691

[59] Carattoli A, Zankari E, García-Fernández A, Voldby Larsen M, Lund O, Villa L, et al. In silico detection and typing of plasmids using PlasmidFinder and plasmid multilocus sequence typing. Antimicrob Agents Chemother. 2014;58(7):3895–903. doi: 10.1128/AAC.02412-14 24777092 PMC4068535

[60] Camacho C, Coulouris G, Avagyan V, Ma N, Papadopoulos J, Bealer K, et al. BLAST+: architecture and applications. BMC Bioinformatics. 2009;10:421. doi: 10.1186/1471-2105-10-421 20003500 PMC2803857

[61] Malberg Tetzschner AM, Johnson JR, Johnston BD, Lund O, Scheutz F. In silico genotyping of escherichia coli isolates for extraintestinal virulence genes by use of whole-genome sequencing data. J Clin Microbiol. 2020;58(10):e01269-20. doi: 10.1128/JCM.01269-20 32669379 PMC7512150

[62] Cosentino S, Voldby Larsen M, Møller Aarestrup F, Lund O. PathogenFinder--distinguishing friend from foe using bacterial whole genome sequence data. PLoS One. 2013;8(10):e77302. doi: 10.1371/journal.pone.0077302 24204795 PMC3810466

[63] Joensen KG, Scheutz F, Lund O, Hasman H, Kaas RS, Nielsen EM, et al. Real-time whole-genome sequencing for routine typing, surveillance, and outbreak detection of verotoxigenic *Escherichia col*i. J Clin Microbiol. 2014;52(5):1501–10. doi: 10.1128/JCM.03617-13 24574290 PMC3993690

[64] Sousa AM, Machado I, Nicolau A, Pereira MO. Improvements on colony morphology identification towards bacterial profiling. J Microbiol Methods. 2013;95(3):327–35. doi: 10.1016/j.mimet.2013.09.020 24121049

[65] Miwa H, Ahmed I, Yokota A, Fujiwara T. Lysinibacillus parviboronicapiens sp. nov., a low-boron-containing bacterium isolated from soil. Int J Syst Evol Microbiol. 2009;59(Pt 6):1427–32. doi: 10.1099/ijs.0.65455-0 19502328

[66] Burkett-Cadena M, Sastoque L, Cadena J, Dunlap CA. Lysinibacillus capsici sp. nov, isolated from the rhizosphere of a pepper plant. Antonie Van Leeuwenhoek. 2019;112(8):1161–7. doi: 10.1007/s10482-019-01248-w 30820713

[67] Tian H, Shi C, Ma Z, Zhou H, Fan W, Zhang W, et al. Physiological characteristics of Bacillus strains originated from dairy products and their impacts on rheological properties of pasteurised yoghurt. Int J of Dairy Tech. 2025;78(1). doi: 10.1111/1471-0307.13169

[68] Lindsay D, Brözel VS, Mostert JF, von Holy A. Physiology of dairy-associated Bacillus spp. over a wide pH range. Int J Food Microbiol. 2000;54(1–2):49–62. doi: 10.1016/s0168-1605(99)00178-6 10746574

[69] Xu K, Yuan Z, Rayner S, Hu X. Genome comparison provides molecular insights into the phylogeny of the reassigned new genus Lysinibacillus. BMC Genomics. 2015;16(1):140. doi: 10.1186/s12864-015-1359-x 25888315 PMC4363355

[70] Peña-Montenegro TD, Lozano L, Dussán J. Genome sequence and description of the mosquitocidal and heavy metal tolerant strain Lysinibacillus sphaericus CBAM5. Stand Genomic Sci. 2015;10:2. doi: 10.1186/1944-3277-10-2 25685257 PMC4317669

[71] Khachatryan L, de Leeuw RH, Kraakman MEM, Pappas N, Te Raa M, Mei H, et al. Taxonomic classification and abundance estimation using 16S and WGS-A comparison using controlled reference samples. Forensic Sci Int Genet. 2020;46:102257. doi: 10.1016/j.fsigen.2020.102257 32058299

[72] Tallur PN, Sajjan DB, Mulla SI, Talwar MP, Pragasam A, Nayak VM, et al. Characterization of antibiotic resistant and enzyme producing bacterial strains isolated from the Arabian Sea. 3 Biotech. 2016;6(1):28. doi: 10.1007/s13205-015-0332-3 28330094 PMC4711286

[73] Khadka S, Adhikari S, Thapa A, Panday R, Adhikari M, Sapkota S, et al. Screening and Optimization of Newly Isolated Thermotolerant Lysinibacillus fusiformis Strain SK for Protease and Antifungal Activity. Curr Microbiol. 2020;77(8):1558–68. doi: 10.1007/s00284-020-01976-7 32248284

[74] Shekarabi M, Hajikhani B, Salimi Chirani A, Fazeli M, Goudarzi M. Molecular characterization of vancomycin-resistant Staphylococcus aureus strains isolated from clinical samples: a three year study in Tehran, Iran. PLoS One. 2017;12(8):e0183607. doi: 10.1371/journal.pone.0183607 28854219 PMC5576738

[75] Ahmed MO, Baptiste KE. Vancomycin-Resistant Enterococci: A Review of Antimicrobial Resistance Mechanisms and Perspectives of Human and Animal Health. Microb Drug Resist. 2018;24(5):590–606. doi: 10.1089/mdr.2017.0147 29058560

[76] Meletis G. Carbapenem resistance: overview of the problem and future perspectives. Ther Adv Infect Dis. 2016;3(1):15–21. doi: 10.1177/2049936115621709 26862399 PMC4735501

[77] von Wintersdorff CJ, et al. Dissemination of antimicrobial resistance in microbial ecosystems through horizontal gene transfer. Front Microbiol. 2016;7:173. 26925045 10.3389/fmicb.2016.00173 PMC4759269

[78] Dutta C, Pan A. Horizontal gene transfer and bacterial diversity. J Biosci. 2002;27(1 Suppl 1):27–33. doi: 10.1007/BF02703681 11927775

[79] Bjorland J, Steinum T, Sunde M, Waage S, Heir E. Novel plasmid-borne gene qacJ mediates resistance to quaternary ammonium compounds in equine Staphylococcus aureus, Staphylococcus simulans, and Staphylococcus intermedius. Antimicrob Agents Chemother. 2003;47(10):3046–52. doi: 10.1128/AAC.47.10.3046-3052.2003 14506007 PMC201118

[80] Gaurav A, Bakht P, Saini M, Pandey S, Pathania R. Role of bacterial efflux pumps in antibiotic resistance, virulence, and strategies to discover novel efflux pump inhibitors. Microbiology (Reading). 2023;169(5):001333. doi: 10.1099/mic.0.001333 37224055 PMC10268834

[81] Short FL, Liu Q, Shah B, Clift HE, Naidu V, Li L, et al. The Acinetobacter baumannii disinfectant resistance protein, AmvA, is a spermidine and spermine efflux pump. Commun Biol. 2021;4(1):1114. doi: 10.1038/s42003-021-02629-6 34552198 PMC8458285

[82] Nguyen THT, et al. Efflux pump inhibitors in controlling antibiotic resistance: outlook under a heavy metal contamination context. Molecules. 2023;28(7). 10.3390/molecules28072912 PMC10095785 37049674

[83] Adamski P, et al. Prevalence and antibiotic resistance of *Bacillus sp.* isolated from raw milk. Microorganisms. 2023;11(4). 10.3390/microorganisms11041065 PMC10143217 37110488

[84] Kresken M, Klare I, Wichelhaus TA, Wohlfarth E, Layer-Nicolaou F, Neumann B, et al. Glycopeptide resistance in Enterococcus spp. and coagulase-negative staphylococci from hospitalised patients in Germany: occurrence, characteristics and dalbavancin susceptibility. J Glob Antimicrob Resist. 2022;28:102–7. doi: 10.1016/j.jgar.2021.12.016 34958996

[85] Li G, Walker MJ, De Oliveira DMP. Vancomycin Resistance in Enterococcus and Staphylococcus aureus. Microorganisms. 2022;11(1):24. doi: 10.3390/microorganisms11010024 36677316 PMC9866002

[86] Agarwal M, et al. Multiple lines of evidences reveal mechanisms underpinning mercury resistance and volatilization by Stenotrophomonas sp. MA5 isolated from the Savannah River Site (SRS), USA. Cells. 2019;8(4). 10.3390/cells8040309 PMC6523443 30987227

[87] Vinayamohan PG, Pellissery AJ, Venkitanarayanan K. Role of horizontal gene transfer in the dissemination of antimicrobial resistance in food animal production. Current Opinion in Food Sci. 2022;47:100882. doi: 10.1016/j.cofs.2022.100882

[88] Woods LC, Gorrell RJ, Taylor F, Connallon T, Kwok T, McDonald MJ. Horizontal gene transfer potentiates adaptation by reducing selective constraints on the spread of genetic variation. Proc Natl Acad Sci U S A. 2020;117(43):26868–75. doi: 10.1073/pnas.2005331117 33055207 PMC7604491

[89] Michaelis C, Grohmann E. Horizontal gene transfer of antibiotic resistance genes in biofilms. Antibiotics (Basel). 2023;12(2):328. doi: 10.3390/antibiotics12020328 36830238 PMC9952180

[90] Verraes C, Van Boxstael S, Van Meervenne E, Van Coillie E, Butaye P, Catry B, et al. Antimicrobial resistance in the food chain: a review. Int J Environ Res Public Health. 2013;10(7):2643–69. doi: 10.3390/ijerph10072643 23812024 PMC3734448

[91] Xu C, Kong L, Gao H, Cheng X, Wang X. A review of current bacterial resistance to antibiotics in food animals. Front Microbiol. 2022;13:822689. doi: 10.3389/fmicb.2022.822689 35633728 PMC9133924

[92] Okaiyeto SA, Sutar PP, Chen C, Ni J-B, Wang J, Mujumdar AS, et al. Antibiotic resistant bacteria in food systems: Current status, resistance mechanisms, and mitigation strategies. Agriculture Communications. 2024;2(1):100027. doi: 10.1016/j.agrcom.2024.100027

[93] Agarwal M, Rathore RS, Chauhan A. A rapid and high throughput MIC determination method to screen uranium resistant microorganisms. Methods Protoc. 2020;3(1). 10.3390/mps3010021 PMC7189662 32138252

[94] Del Duca S, et al. Microbial genetics and evolution. Microorganisms. 2022;10(7). 10.3390/microorganisms10071274 PMC9315481 35888993

[95] Arber W. Genetic variation: molecular mechanisms and impact on microbial evolution. FEMS Microbiol Rev. 2000;24(1):1–7. doi: 10.1111/j.1574-6976.2000.tb00529.x 10640595

[96] Hilário S, Gonçalves MFM, Matos I, Rangel LF, Sousa JA, Santos MJ, et al. Comparative genomics reveals insights into the potential of Lysinibacillus irui as a plant growth promoter. Appl Microbiol Biotechnol. 2024;108(1):370. doi: 10.1007/s00253-024-13210-6 38861018 PMC11166776

[97] Yin Q-J, Ying T-T, Zhou Z-Y, Hu G-A, Yang C-L, Hua Y, et al. Species-specificity of the secondary biosynthetic potential in Bacillus. Front Microbiol. 2023;14:1271418. doi: 10.3389/fmicb.2023.1271418 37937215 PMC10626522

[98] Wiedenbeck J, Cohan FM. Origins of bacterial diversity through horizontal genetic transfer and adaptation to new ecological niches. FEMS Microbiol Rev. 2011;35(5):957–76. doi: 10.1111/j.1574-6976.2011.00292.x 21711367

[99] Cordero OX, Hogeweg P. The impact of long-distance horizontal gene transfer on prokaryotic genome size. Proc Natl Acad Sci U S A. 2009;106(51):21748–53. doi: 10.1073/pnas.0907584106 20007373 PMC2799812

[100] Rey A, Silva-Quintero L, Dussan J. Complete genome sequence of the larvicidal bacterium Lysinibacillus sphaericus strain OT4b. Genome Announcements. 2016;4(3). 10.1128/genomeA.00257-16 PMC4859168 27151786

[101] Choi Y, Pham H, Nguyen MP, Tran LVH, Kim J, Kim S, et al. A native conjugative plasmid confers potential selective advantages to plant growth-promoting Bacillus velezensis strain GH1-13. Commun Biol. 2021;4(1):582. doi: 10.1038/s42003-021-02107-z 33990691 PMC8121941

[102] Roy T, Bandopadhyay A, Paul C, Majumdar S, Das N. Role of Plasmid in Pesticide Degradation and Metal Tolerance in Two Plant Growth-Promoting Rhizobacteria Bacillus cereus (NCIM 5557) and Bacillus safensis (NCIM 5558). Curr Microbiol. 2022;79(4):106. doi: 10.1007/s00284-022-02793-w 35157142

[103] Kim SY, Song H, Sang MK, Weon H-Y, Song J. The complete genome sequence of Bacillus velezensis strain GH1-13 reveals agriculturally beneficial properties and a unique plasmid. J Biotechnol. 2017;259:221–7. doi: 10.1016/j.jbiotec.2017.06.1206 28690133

[104] Tang X, Shang J, Ji Y, Sun Y. PLASMe: a tool to identify PLASMid contigs from short-read assemblies using transformer. Nucleic Acids Res. 2023;51(15):e83. doi: 10.1093/nar/gkad578 37427782 PMC10450166

[105] Laczny CC, Galata V, Plum A, Posch AE, Keller A. Assessing the heterogeneity of in silico plasmid predictions based on whole-genome-sequenced clinical isolates. Brief Bioinform. 2019;20(3):857–65. doi: 10.1093/bib/bbx162 29220507

[106] Helfrich EJN, Lin G-M, Voigt CA, Clardy J. Bacterial terpene biosynthesis: challenges and opportunities for pathway engineering. Beilstein J Org Chem. 2019;15:2889–906. doi: 10.3762/bjoc.15.283 31839835 PMC6902898

[107] Papaioannou G, et al. Profile of volatile compounds in dessert yogurts prepared from cow and goat milk, using different starter cultures and probiotics. Foods. 2021;10(12). 10.3390/foods10123153 PMC8701116 34945703

[108] Hudson GA, Mitchell DA. RiPP antibiotics: biosynthesis and engineering potential. Curr Opin Microbiol. 2018;45:61–9. doi: 10.1016/j.mib.2018.02.010 29533845 PMC6131089

[109] Pfeiffer IP-M, Schröder M-P, Mordhorst S. Opportunities and challenges of RiPP-based therapeutics. Nat Prod Rep. 2024;41(7):990–1019. doi: 10.1039/d3np00057e 38411278

[110] Han SW, Won HS. Advancements in the application of ribosomally synthesized and post-translationally modified peptides (RiPPs). Biomolecules. 2024;14(4). 10.3390/biom14040479 PMC11048544 38672495

[111] Fu Y, Jaarsma AH, Kuipers OP. Antiviral activities and applications of ribosomally synthesized and post-translationally modified peptides (RiPPs). Cell Mol Life Sci. 2021;78(8):3921–40. doi: 10.1007/s00018-021-03759-0 33532865 PMC7853169

[112] van Staden ADP, et al. Therapeutic application of lantibiotics and other lanthipeptides: old and new findings. Appl Environ Microbiol. 2021;87(14):e0018621. 10.1128/AEM.00186-21 PMC8231447 33962984

[113] Ramírez-Rendón D, Guzmán-Chávez F, García-Ausencio C, Rodríguez-Sanoja R, Sánchez S. The untapped potential of actinobacterial lanthipeptides as therapeutic agents. Mol Biol Rep. 2023;50(12):10605–16. doi: 10.1007/s11033-023-08880-w 37934370 PMC10676316

[114] Tang W, Dong S-H, Repka LM, He C, Nair SK, van der Donk WA. Applications of the class II lanthipeptide protease LicP for sequence-specific, traceless peptide bond cleavage. Chem Sci. 2015;6(11):6270–9. doi: 10.1039/c5sc02329g 30090246 PMC6054071

[115] Ullah M, Rizwan M, Raza A, Zhao X, Sun Y, Gul S, et al. Comparative Genomic and Functional Characterization of Lactobacillus casei Group (LCG) Probiotic Strains Isolated from Traditional Yogurts by Next-Generation Sequencing. PJZ. 2023;55(4). doi: 10.17582/journal.pjz/20210711190719

[116] Bruna P, Núñez-Montero K, Contreras MJ, Leal K, García M, Abanto M, et al. Biosynthetic gene clusters with biotechnological applications in novel Antarctic isolates from Actinomycetota. Appl Microbiol Biotechnol. 2024;108(1):325. doi: 10.1007/s00253-024-13154-x 38717668 PMC11078813

[117] Xia L, Miao Y, Cao A, Liu Y, Liu Z, Sun X, et al. Biosynthetic gene cluster profiling predicts the positive association between antagonism and phylogeny in Bacillus. Nat Commun. 2022;13(1):1023. doi: 10.1038/s41467-022-28668-z 35197480 PMC8866423

[118] Wu H, Wang D, Gao F. Toward a high-quality pan-genome landscape of *Bacillus subtilis* by removal of confounding strains. Brief Bioinform. 2021;22(2):1951–71. doi: 10.1093/bib/bbaa013 32065216

[119] Agarwal M, Sheikh MB. Isolation and functional characterization of endophytic bacteria from muscadine grape berries: a microbial treasure trove. Cells. 2025;14(5). 10.3390/cells14050369 PMC11899604 40072097

[120] Tettelin H, Riley D, Cattuto C, Medini D. Comparative genomics: the bacterial pan-genome. Curr Opin Microbiol. 2008;11(5):472–7. doi: 10.1016/j.mib.2008.09.006 19086349

[121] Tettelin H, Medini D. In The Pangenome: Diversity, Dynamics and Evolution of Genomes. CH: Cham. 2020. 32633908

[122] Golicz AA, Bayer PE, Bhalla PL, Batley J, Edwards D. Pangenomics Comes of Age: From Bacteria to Plant and Animal Applications. Trends Genet. 2020;36(2):132–45. doi: 10.1016/j.tig.2019.11.006 31882191

[123] Medini D, Donati C, Tettelin H, Masignani V, Rappuoli R. The microbial pan-genome. Curr Opin Genet Dev. 2005;15(6):589–94. doi: 10.1016/j.gde.2005.09.006 16185861

[124] Hurtado-Páez U, Álvarez Zuluaga N, Arango Isaza RE, Contreras-Moreira B, Rouzaud F, Robledo J. Pan-genome association study of Mycobacterium tuberculosis lineage-4 revealed specific genes related to the high and low prevalence of the disease in patients from the North-Eastern area of Medellín, Colombia. Front Microbiol. 2023;13:1076797. doi: 10.3389/fmicb.2022.1076797 36687645 PMC9846648

[125] Zhang X, et al. Distinct drivers of core and accessory components of soil microbial community functional diversity under environmental changes. mSystems. 2019;4(5). 10.1128/mSystems.00374-19 PMC6774018 31575666

[126] Hyun JC, Monk JM, Palsson BO. Comparative pangenomics: analysis of 12 microbial pathogen pangenomes reveals conserved global structures of genetic and functional diversity. BMC Genomics. 2022;23(1):7. doi: 10.1186/s12864-021-08223-8 34983386 PMC8725406

[127] Sharma R, Nimonkar Y, Sharma A, Rathore RS, Prakash O. Concept of microbial preservation: past, present and future. Soil Biology. Springer International Publishing. 2018. p. 35–54. doi: 10.1007/978-3-319-96971-8_2

[128] Rouli L, Merhej V, Fournier P-E, Raoult D. The bacterial pangenome as a new tool for analysing pathogenic bacteria. New Microbes New Infect. 2015;7:72–85. doi: 10.1016/j.nmni.2015.06.005 26442149 PMC4552756

[129] Francke C, Siezen RJ, Teusink B. Reconstructing the metabolic network of a bacterium from its genome. Trends Microbiol. 2005;13(11):550–8. doi: 10.1016/j.tim.2005.09.001 16169729

[130] Galperin MY, Kristensen DM, Makarova KS, Wolf YI, Koonin EV. Microbial genome analysis: the COG approach. Brief Bioinform. 2019;20(4):1063–70. doi: 10.1093/bib/bbx117 28968633 PMC6781585

[131] Ricker N, Qian H, Fulthorpe RR. The limitations of draft assemblies for understanding prokaryotic adaptation and evolution. Genomics. 2012;100(3):167–75. doi: 10.1016/j.ygeno.2012.06.009 22750556

