# Supplementary figures and images for: Whole-genome analysis of Lysinibacillus boronitolerans MSR1: A dairy-isolated multidrug-resistant and non-pathogenic strain
Source: PLoS One. 2025 Dec 12;20(12):e0333844. doi: 10.1371/journal.pone.0333844 (PMC12700380; doi:10.1371/journal.pone.0333844)

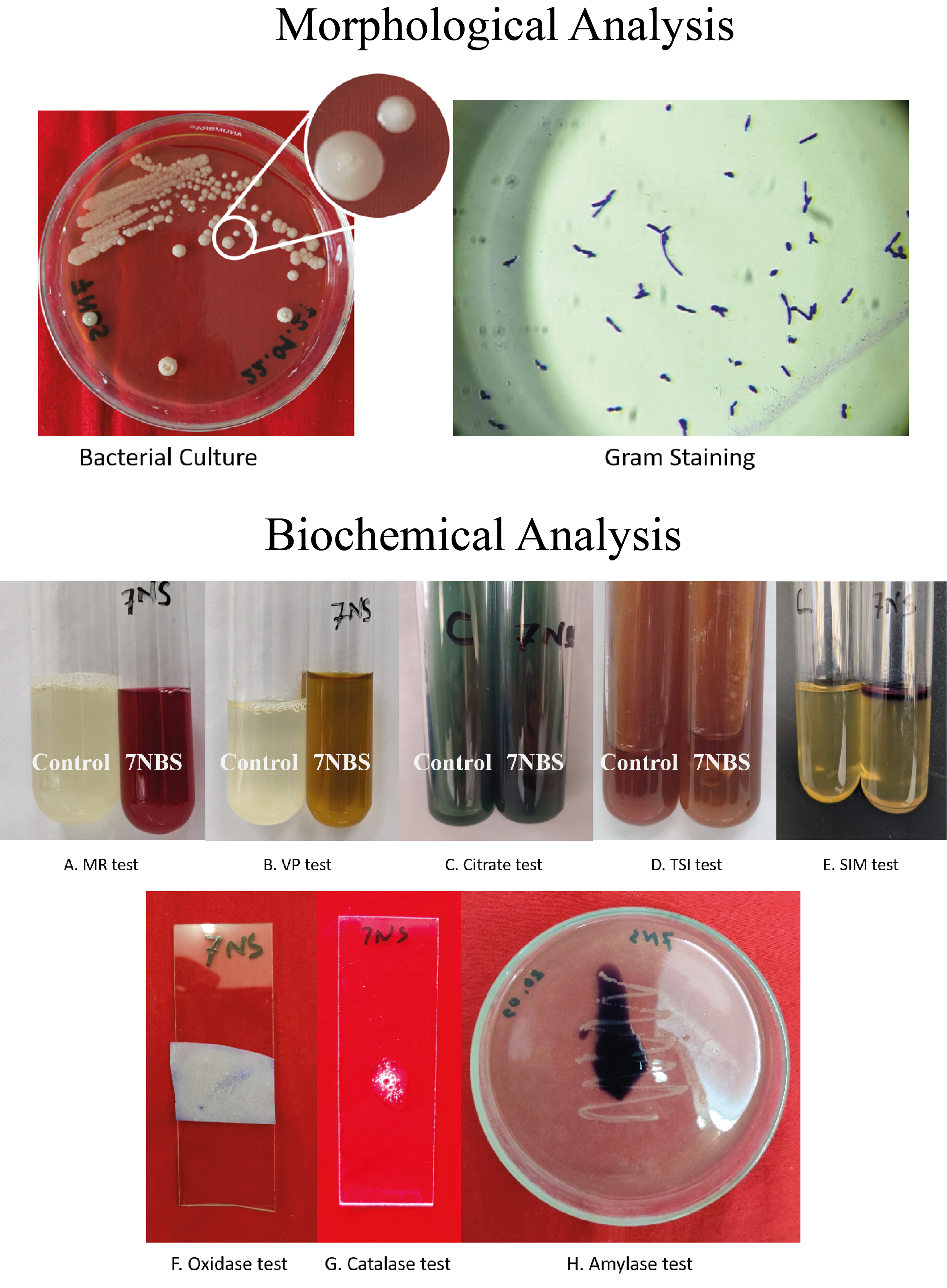

Supplement: S1 Fig — (PNG) [file pone.0333844.s001.png]

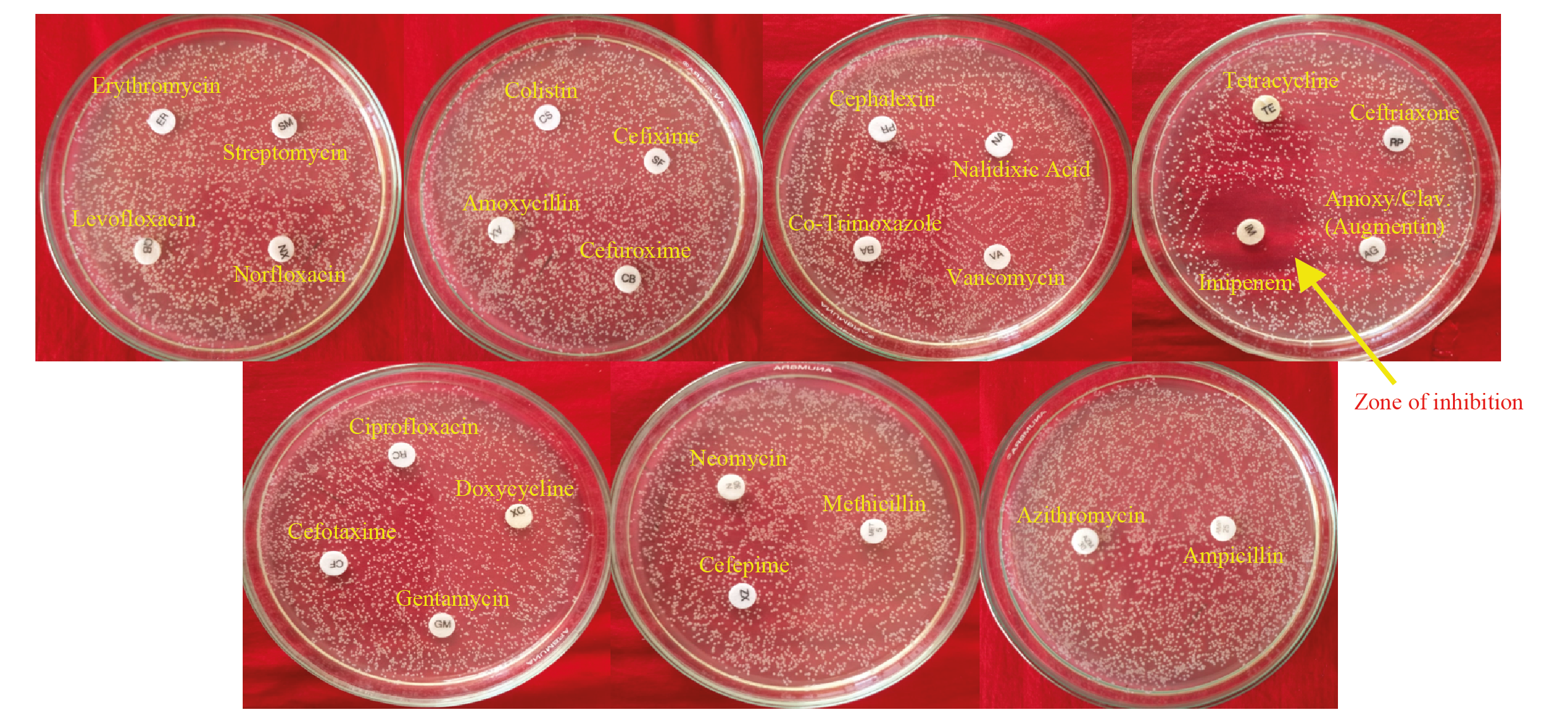

Supplement: S2 Fig — (PNG) [file pone.0333844.s002.png]

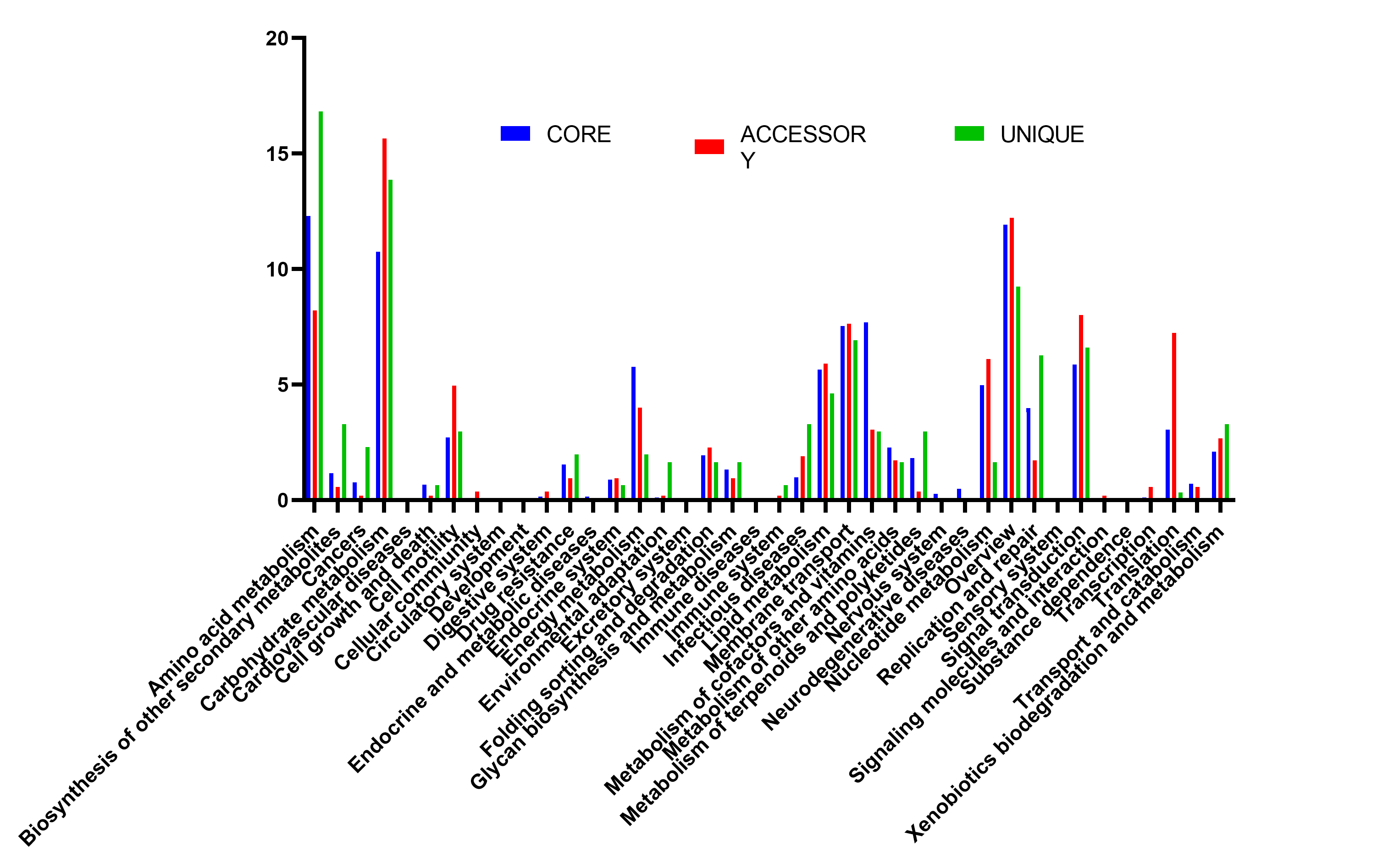

Supplement: S3 Fig — (PNG) [file pone.0333844.s003.png]

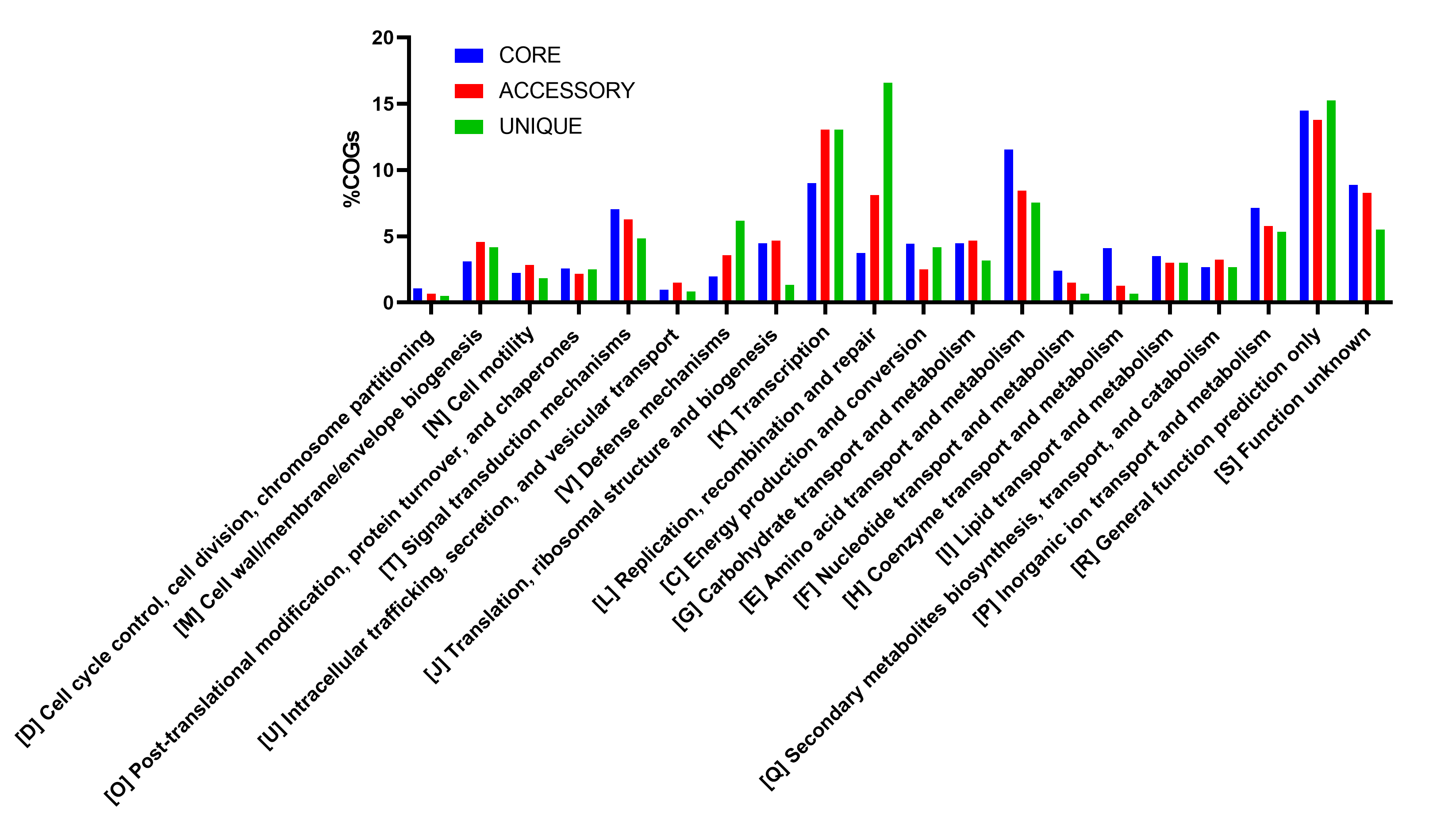

Supplement: S4 Fig — (PNG) [file pone.0333844.s004.png]

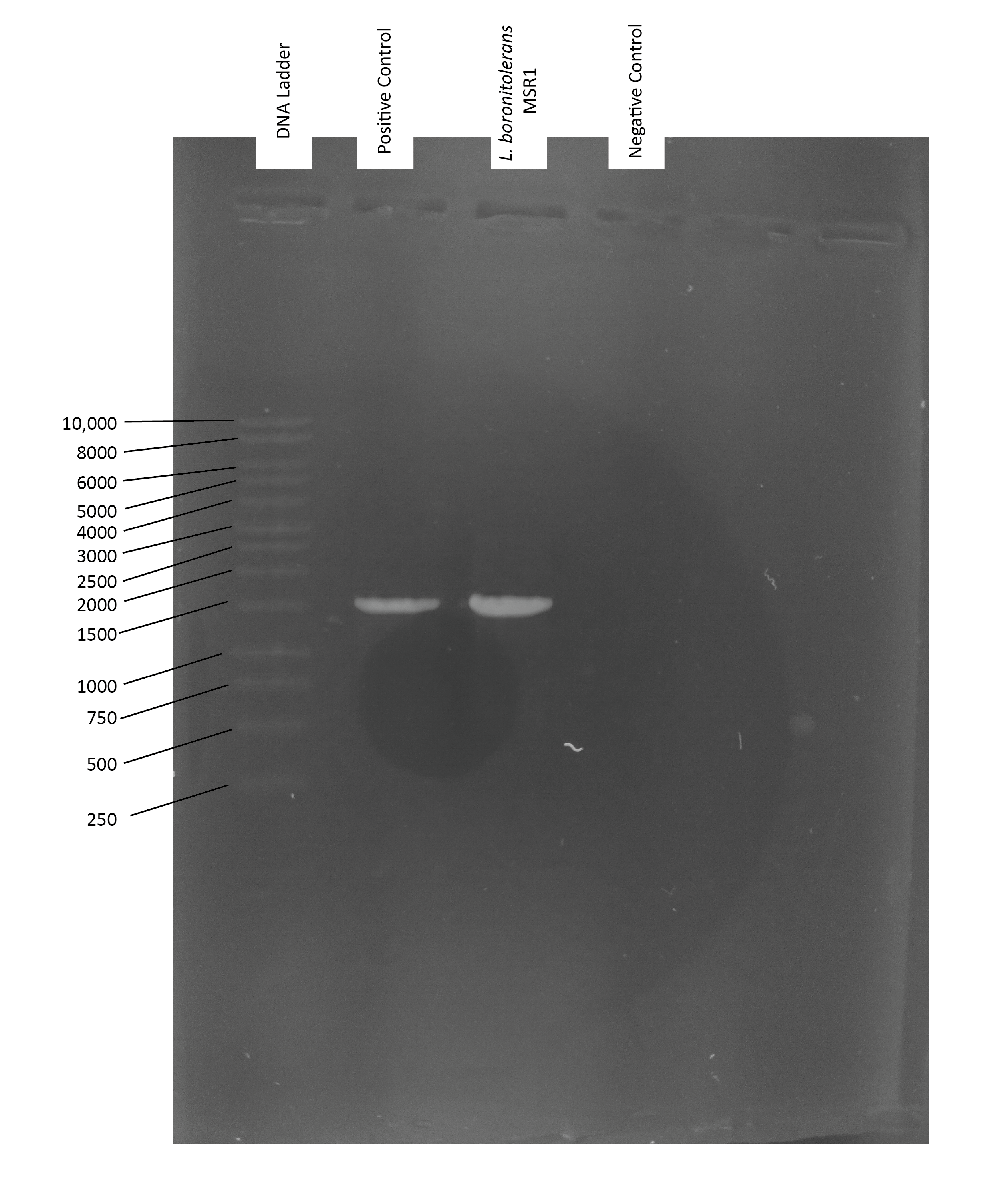

Supplement: S5 Fig — Gel documentation image of L. boronitolerans MSR1. (PNG) [file pone.0333844.s005.png]

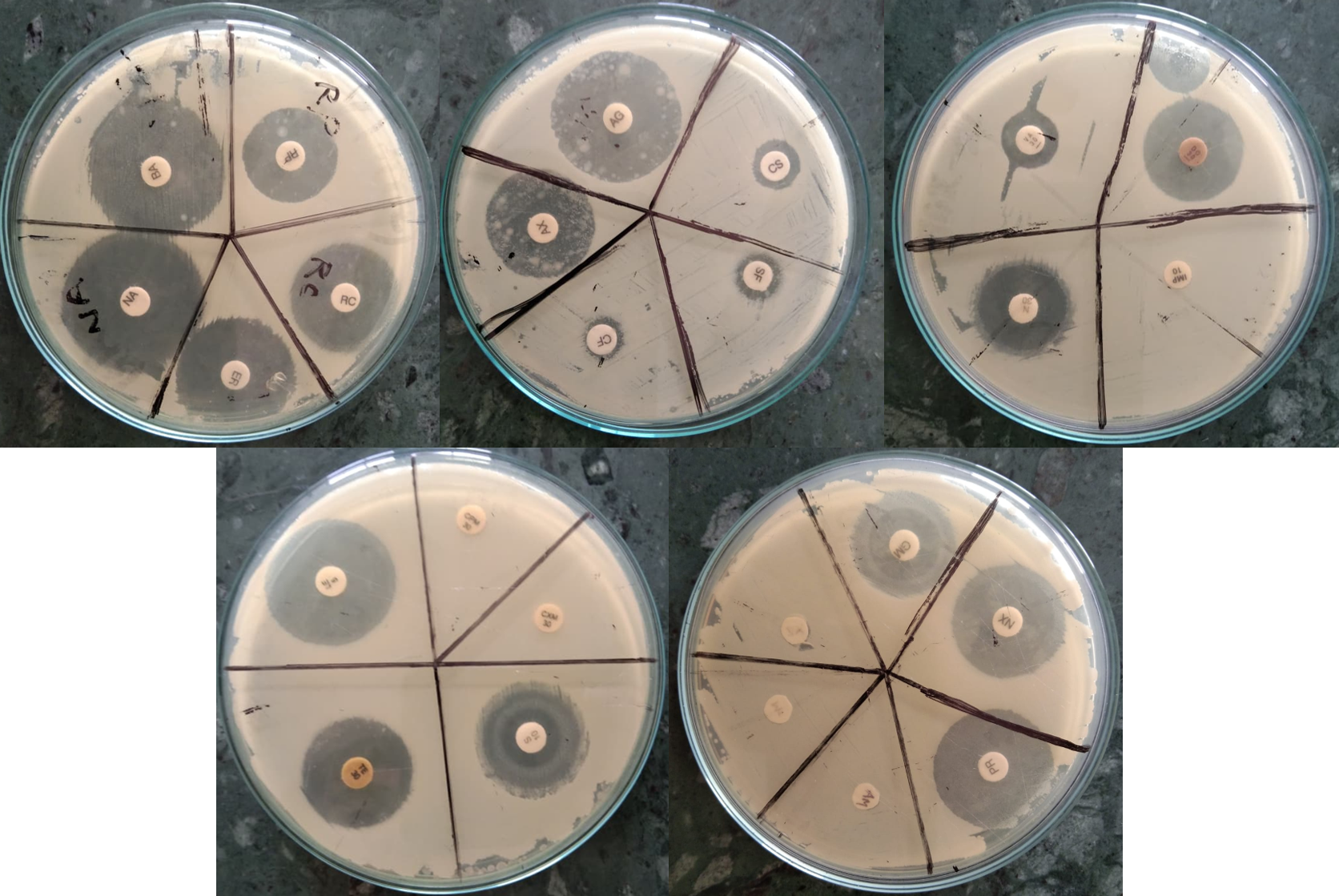

Supplement: S6 Fig — (PNG) [file pone.0333844.s006.png]

## COG Distribution

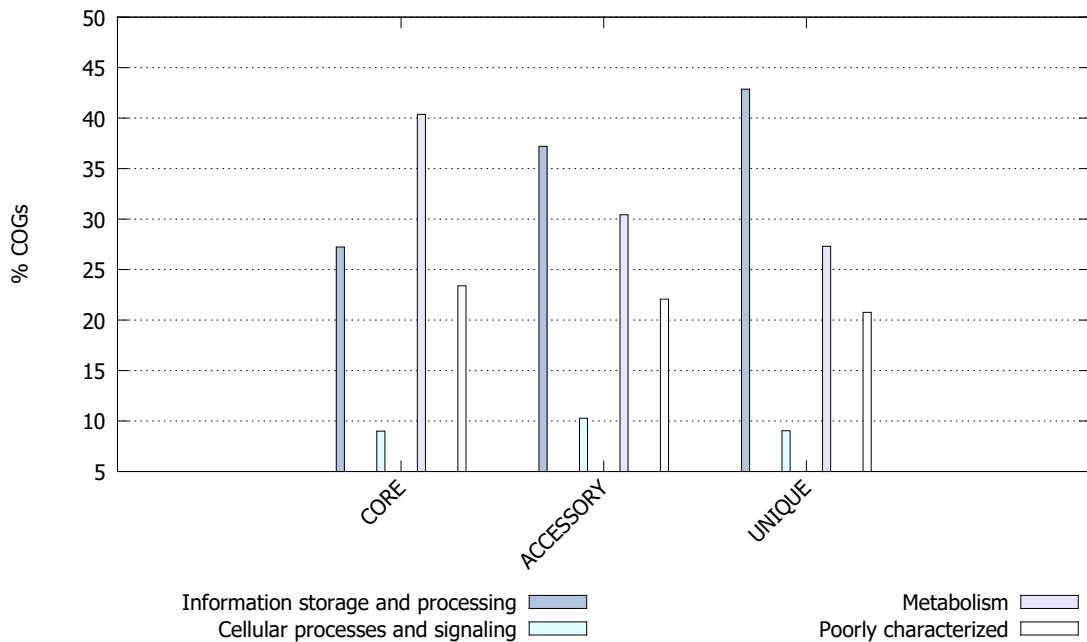

Supplement: S7 File — (ZIP) [file pone.0333844.s013.zip › COG/COG_DISTRIBUTION.pdf]

# COG Distribution

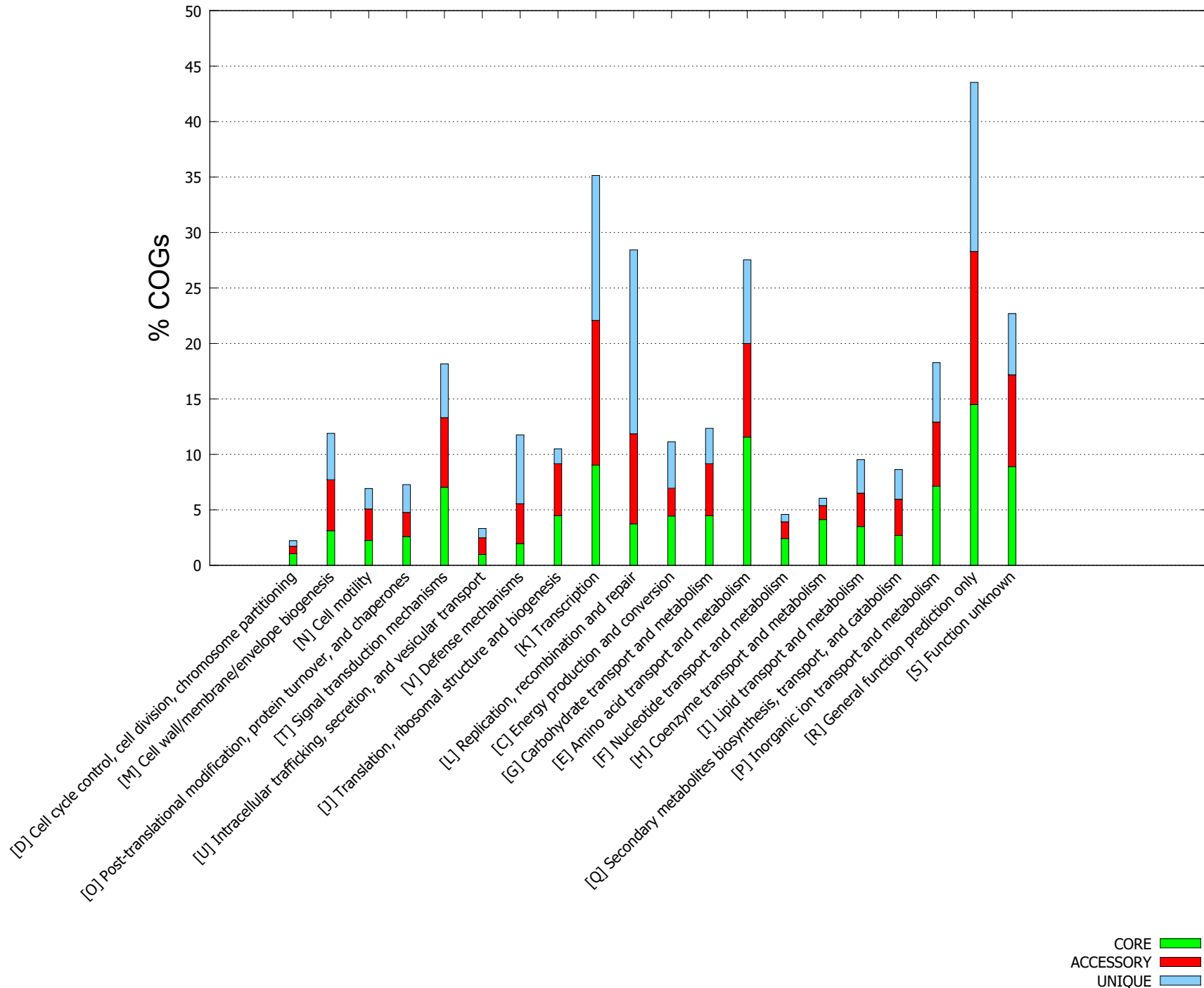

Supplement: S7 File — (ZIP) [file pone.0333844.s013.zip › COG/COG_DISTRIBUTION_DETAILS.pdf]

## KEGG Distribution

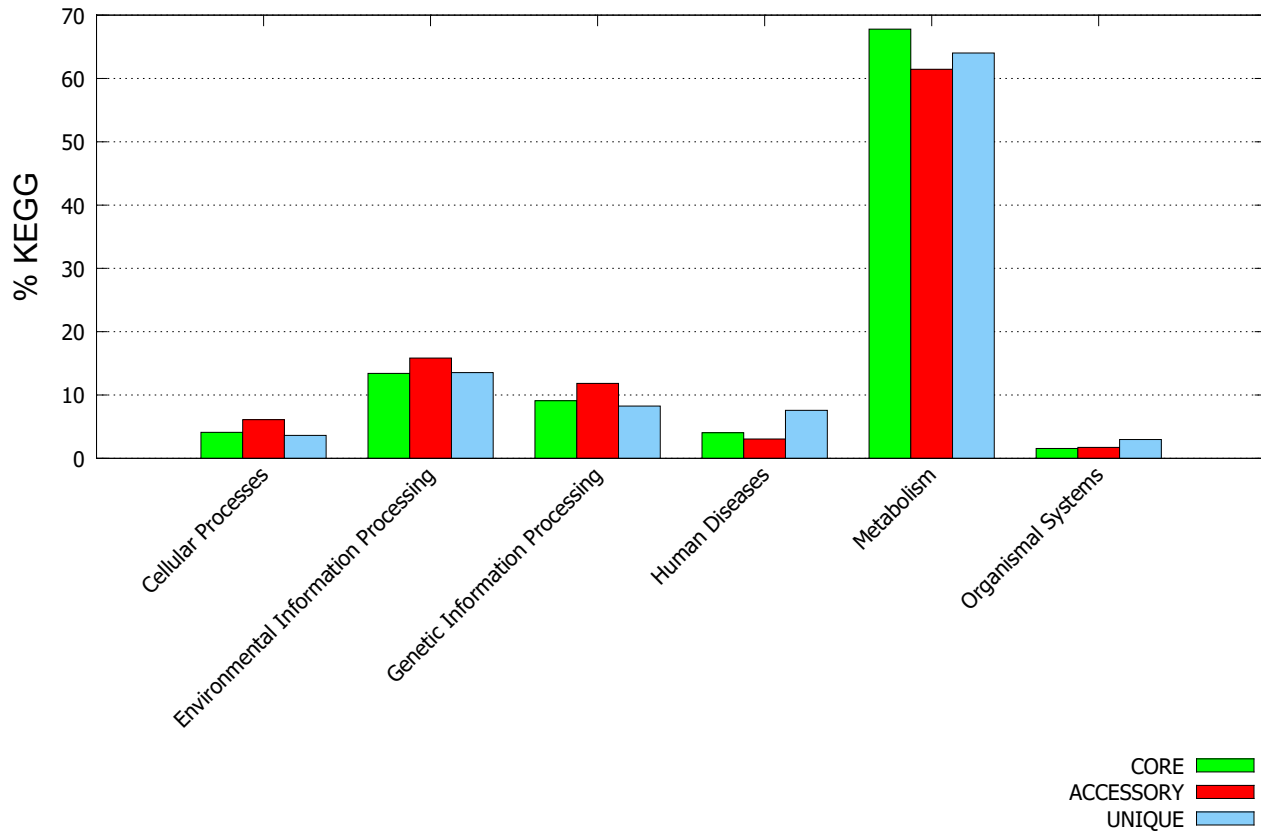

Supplement: S7 File — (ZIP) [file pone.0333844.s013.zip › KEGG/KEGG_DISTRIBUTION.pdf]

# KEGG Distribution

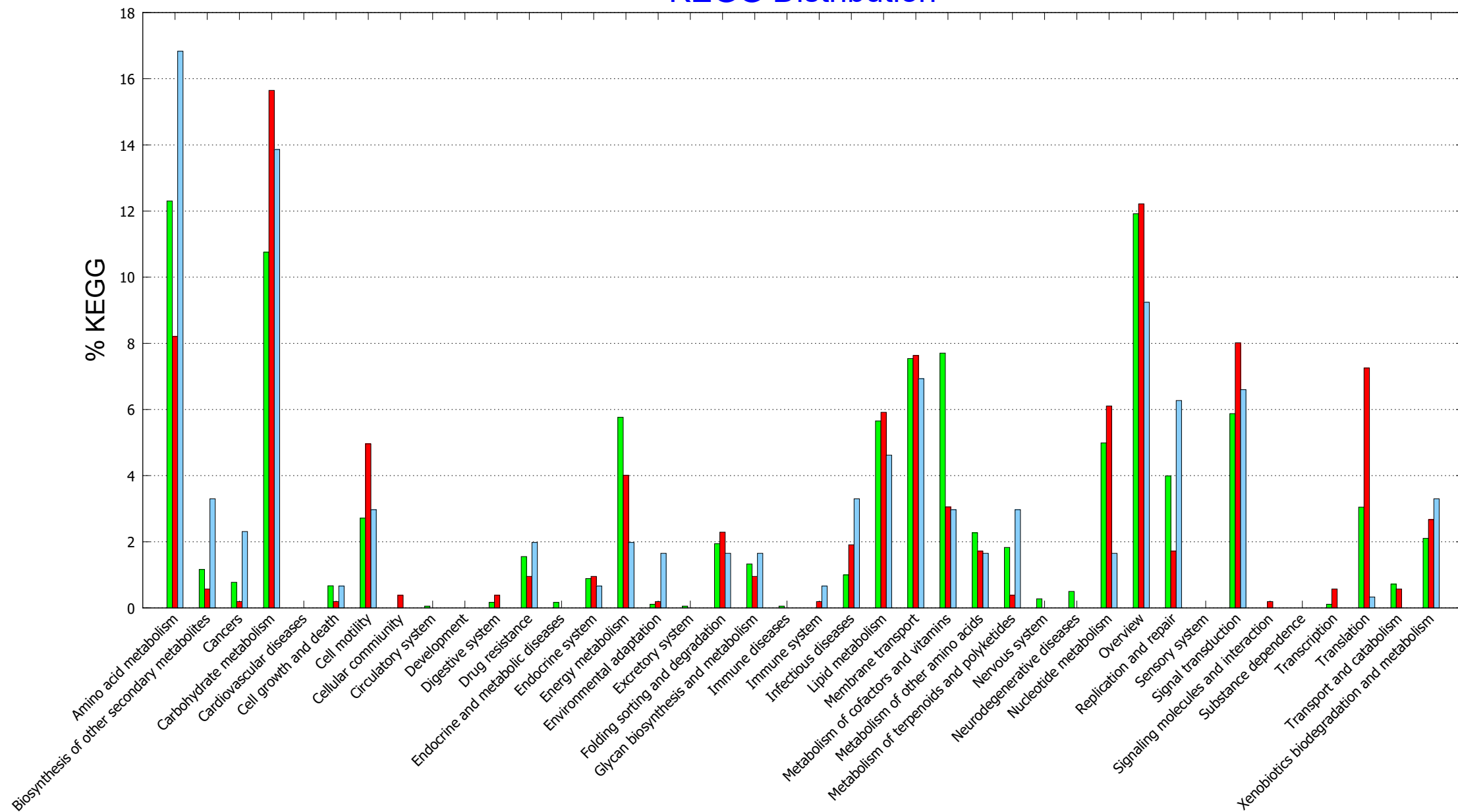

CORE  
ACCESSORY  
UNIQUE

Supplement: S7 File — (ZIP) [file pone.0333844.s013.zip › KEGG/KEGG_DISTRIBUTION_DETAILS.pdf]

Core and Pan Genome Plot

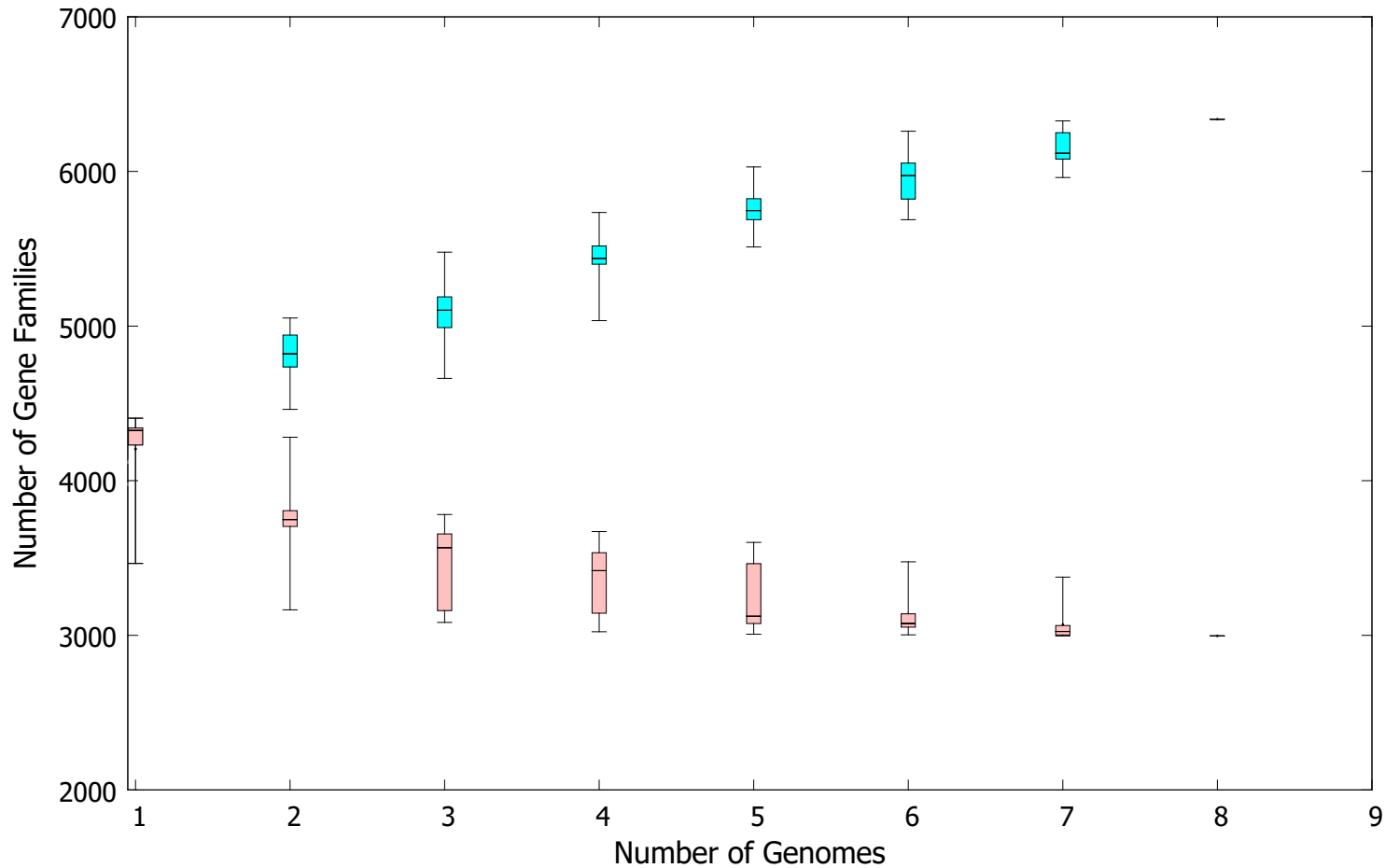

Pan Genome  
Core Genome  
Median Values

Supplement: S8 File — (ZIP) [file pone.0333844.s014.zip › PAN/Core_Pan_Plot.pdf]

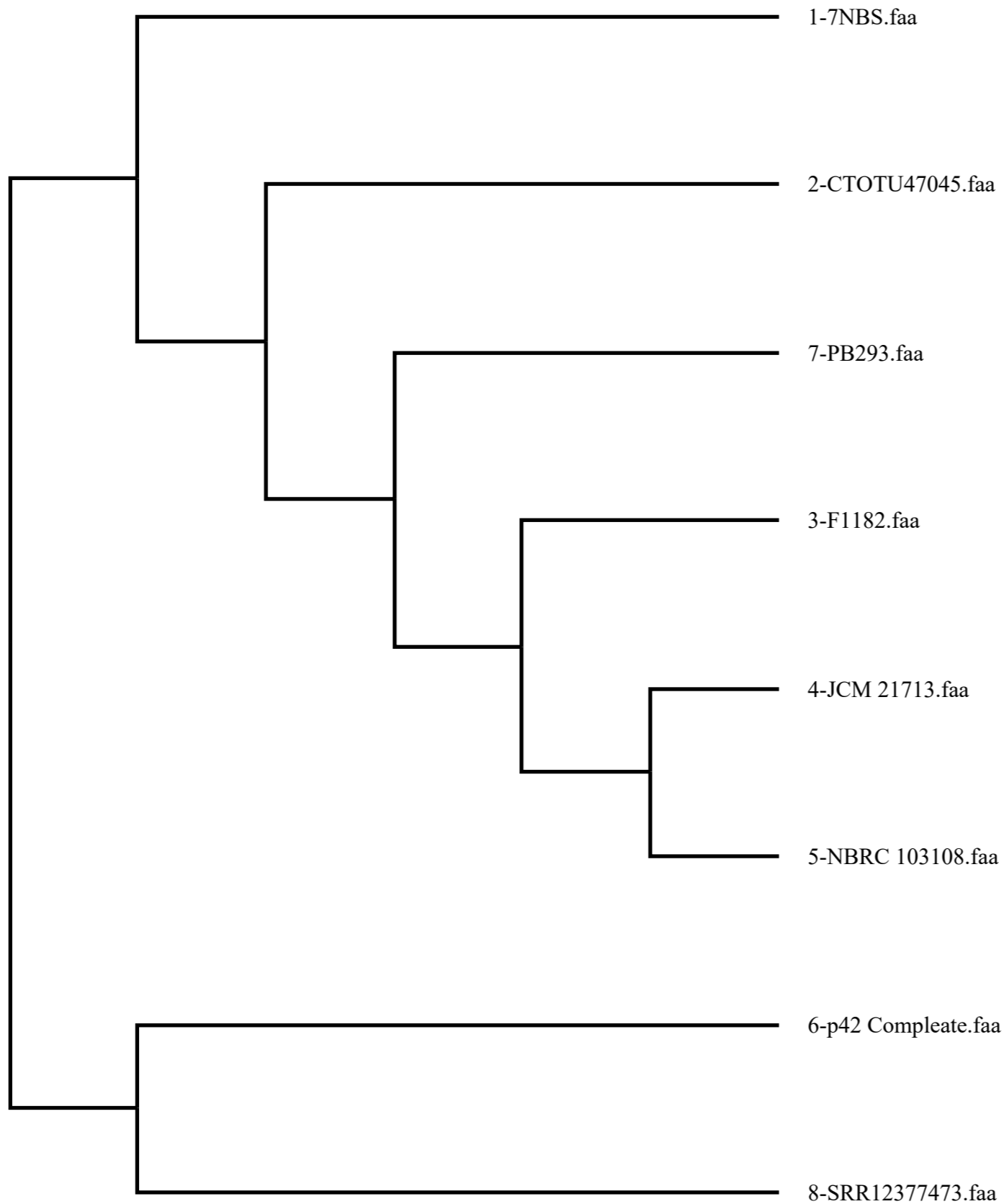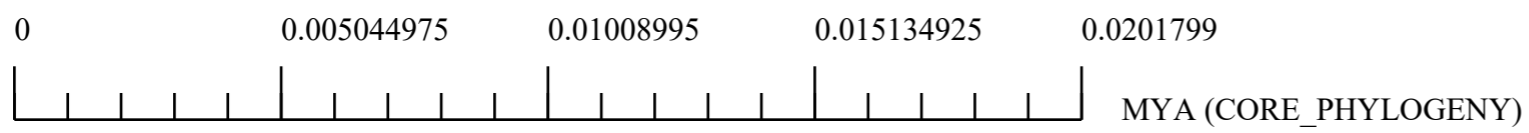

Supplement: S8 File — (ZIP) [file pone.0333844.s014.zip › PAN/Core_phylogeny.pdf]

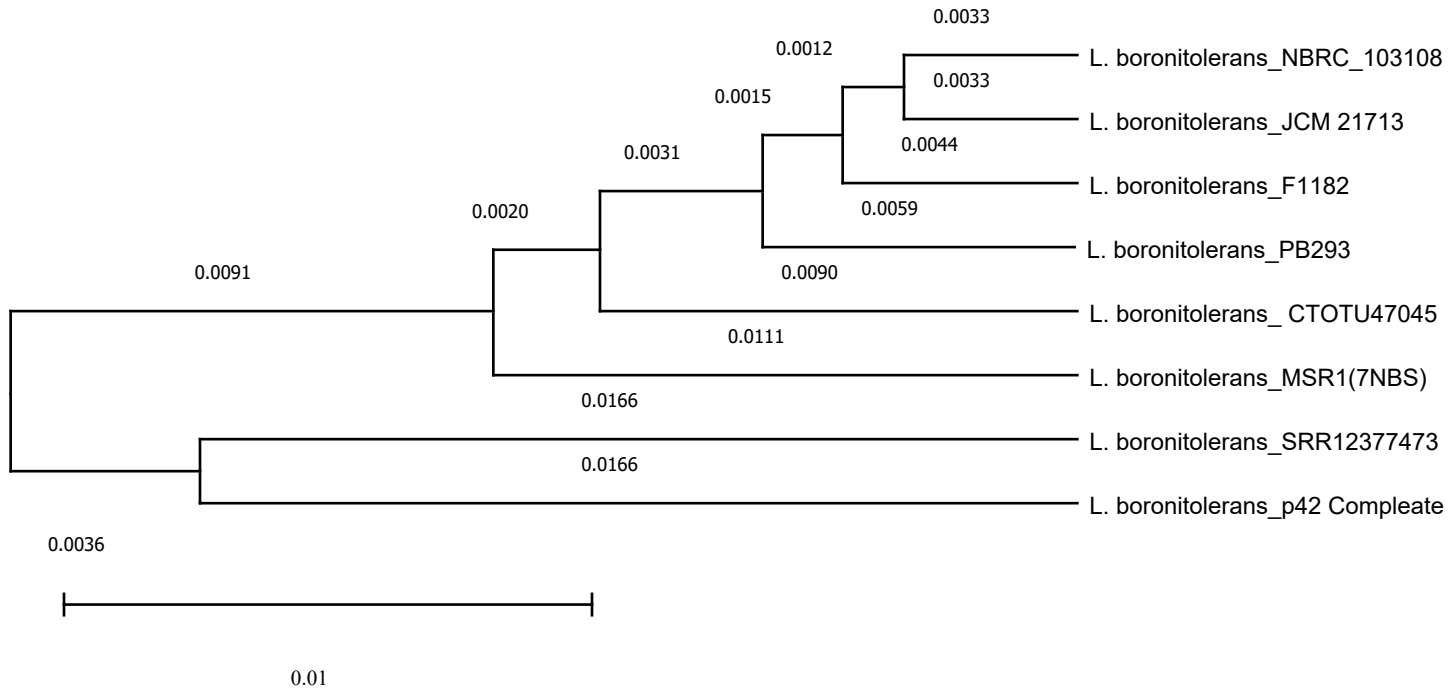

Supplement: S8 File — (ZIP) [file pone.0333844.s014.zip › PAN/Core_phylogeny_tree.pdf]

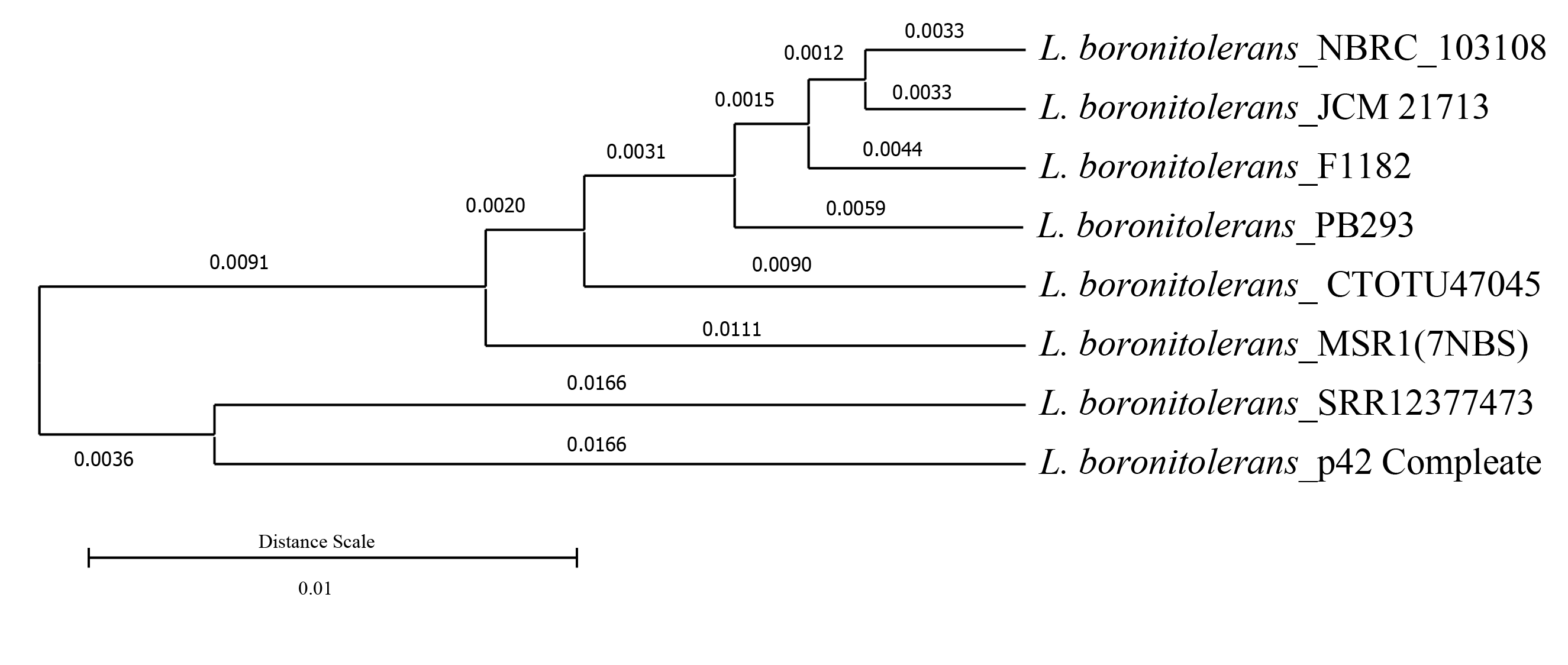

Supplement: S8 File — (ZIP) [file pone.0333844.s014.zip › PAN/Core_phylogeny_tree.png]

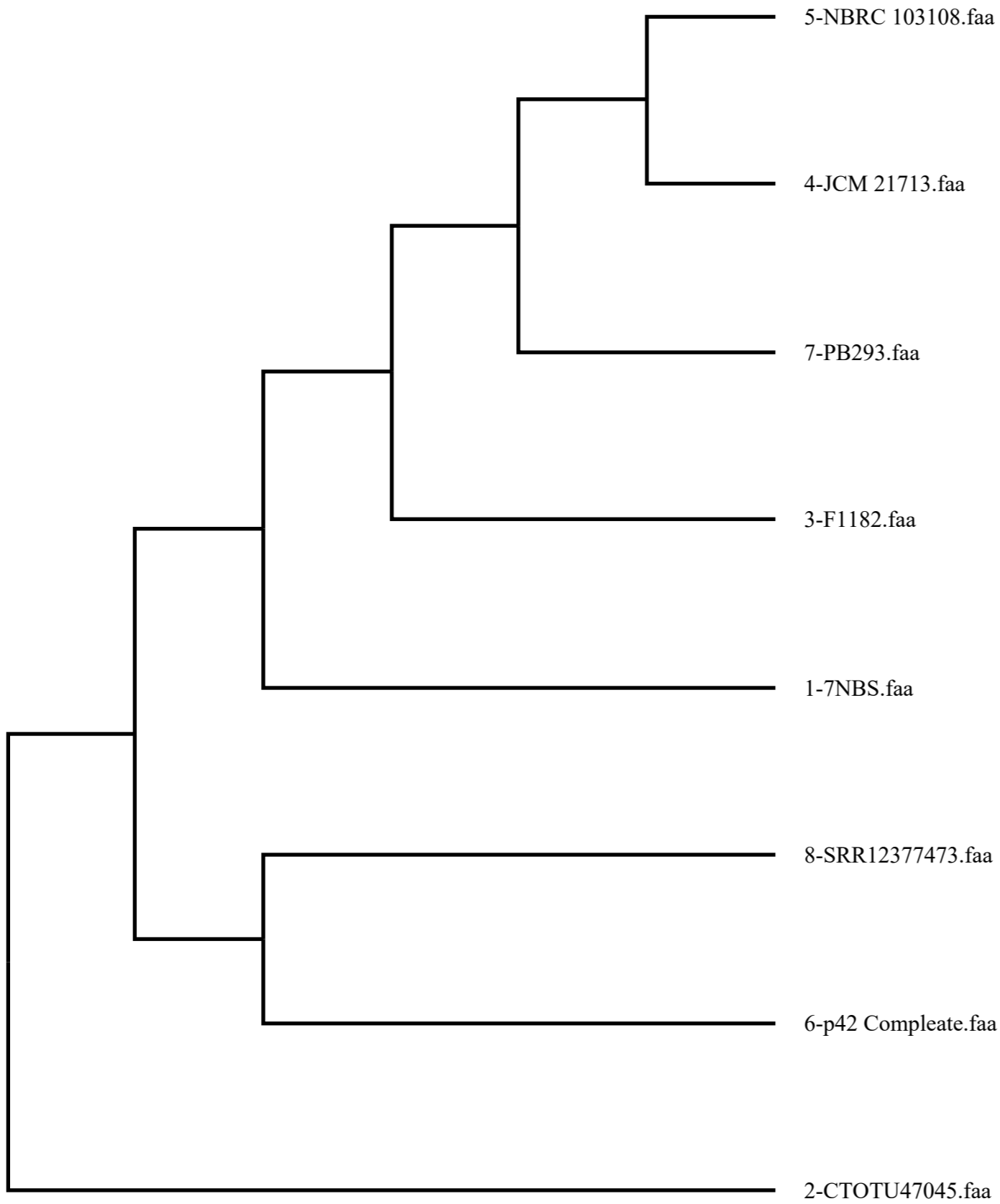

0 0.048378475 0.09675695 0.145135425 0.1935139

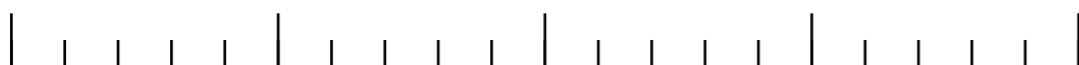

MYA (PAN\_PHYLOGENY)

Supplement: S8 File — (ZIP) [file pone.0333844.s014.zip › PAN/Pan_phylogeny.pdf]

Core-Pan Plot

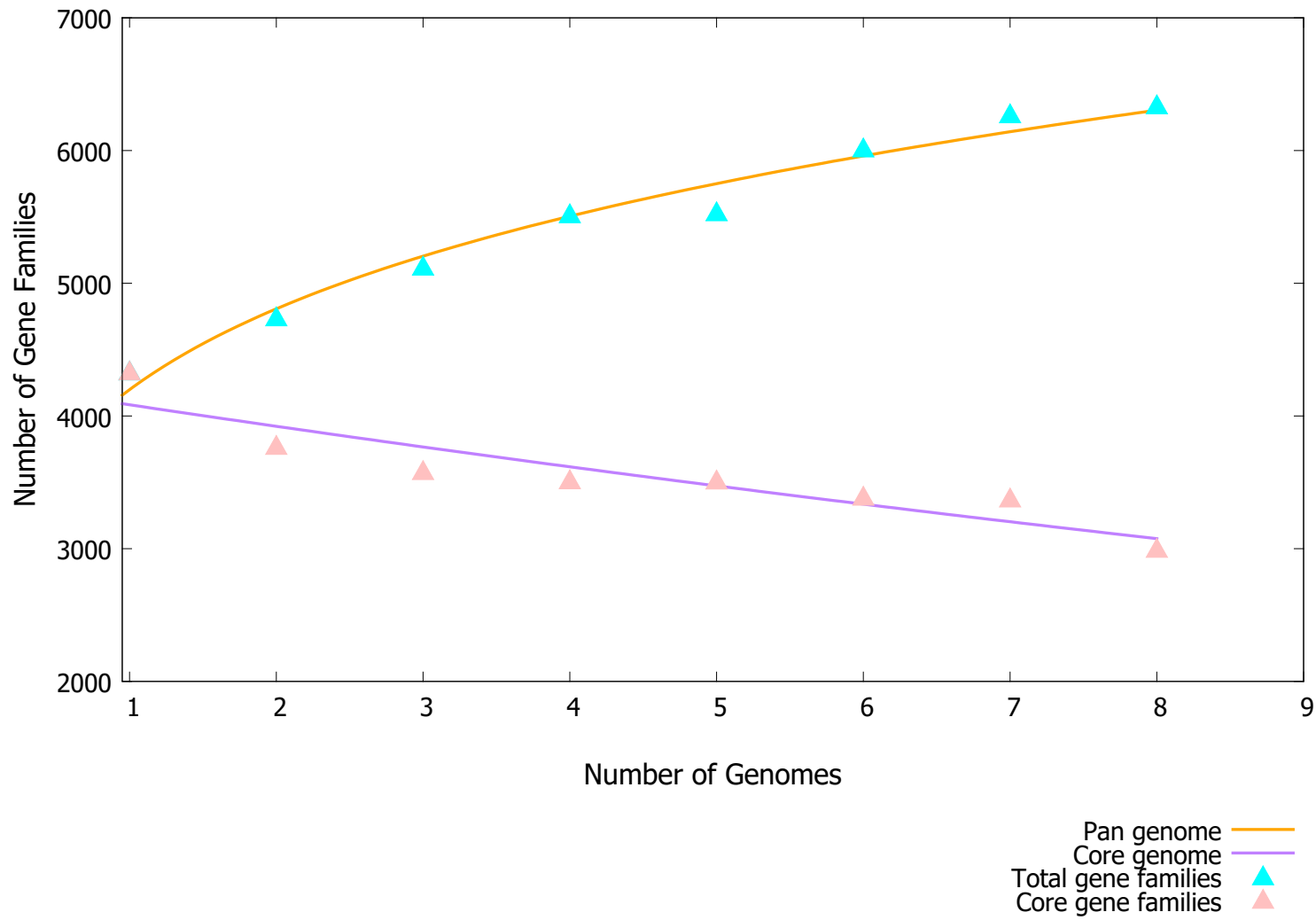

Supplement: S8 File — (ZIP) [file pone.0333844.s014.zip › Results/Default_Core_Pan_Plot.pdf]

Distribution of Gene Families

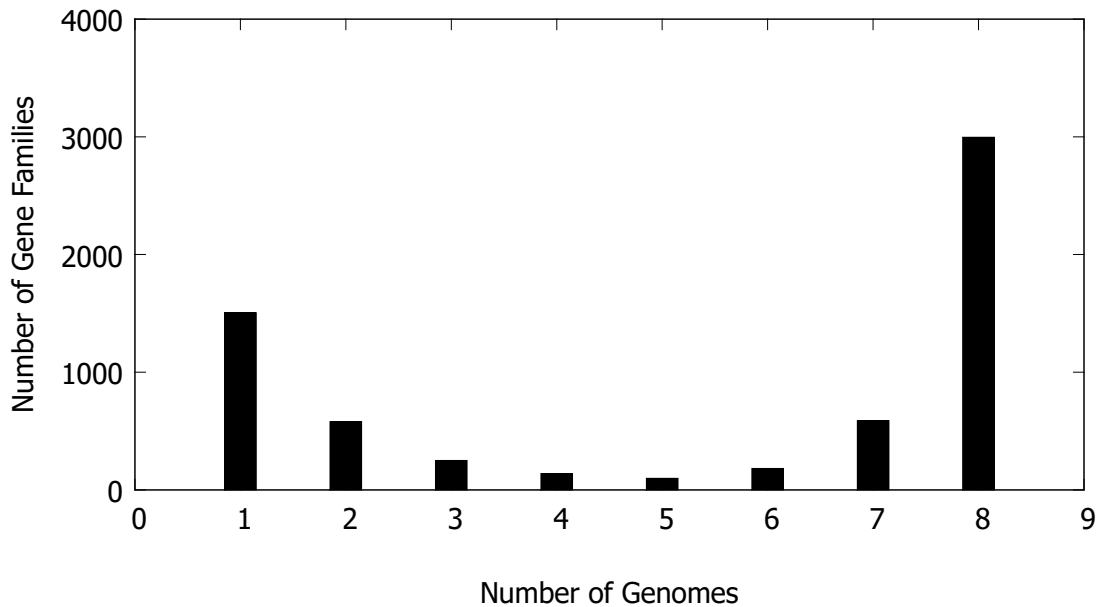

Supplement: S8 File — (ZIP) [file pone.0333844.s014.zip › Results/Histogram.pdf]

## Number of New Genes

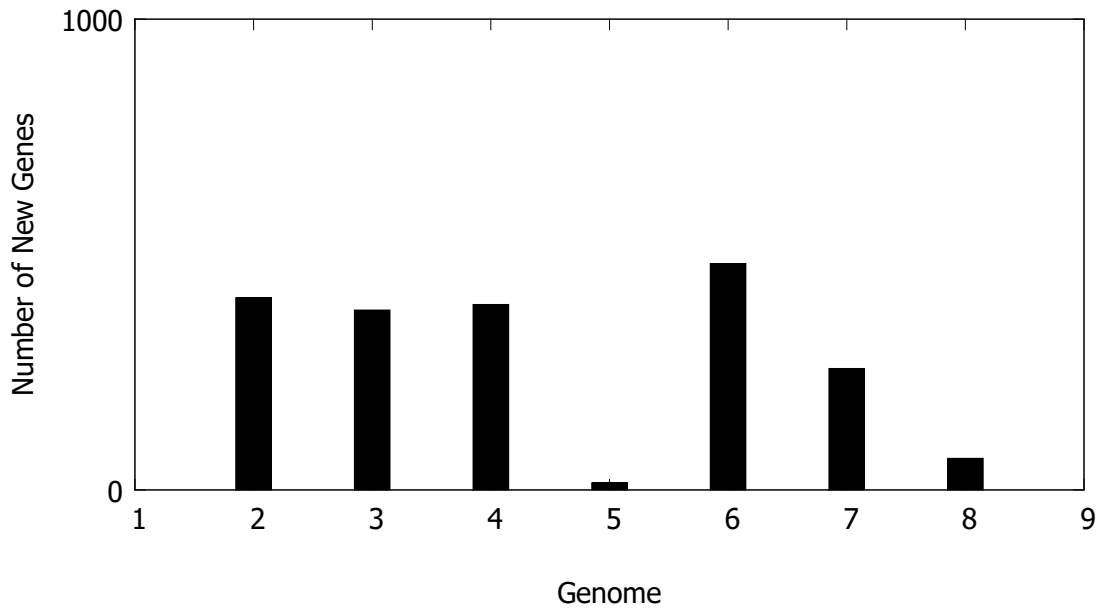

Supplement: S8 File — (ZIP) [file pone.0333844.s014.zip › Results/New_Genes_Plot.pdf]

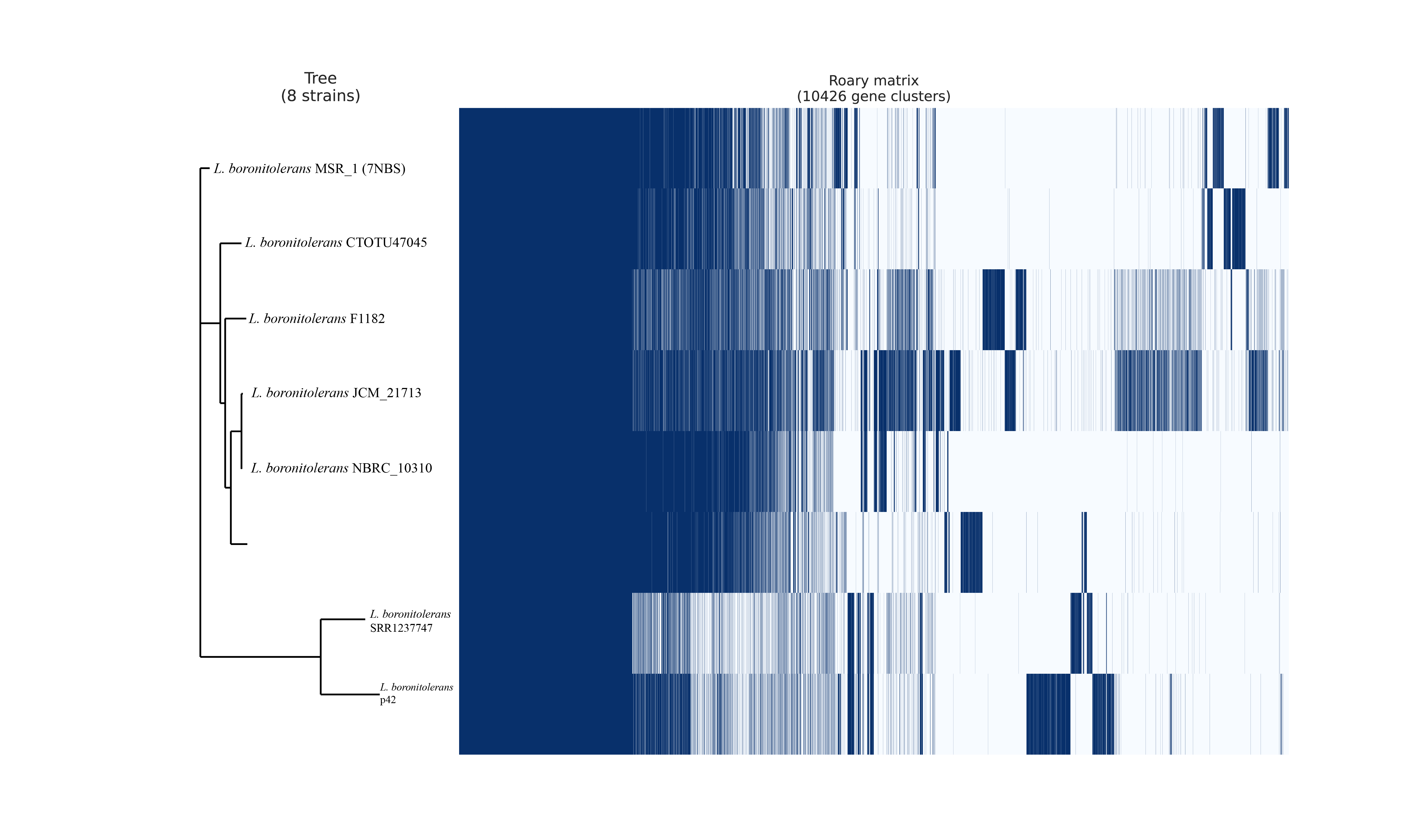

Supplement: S9 File — (ZIP) [file pone.0333844.s015.zip › roaryresult/Final_pangenome_matrix.png]

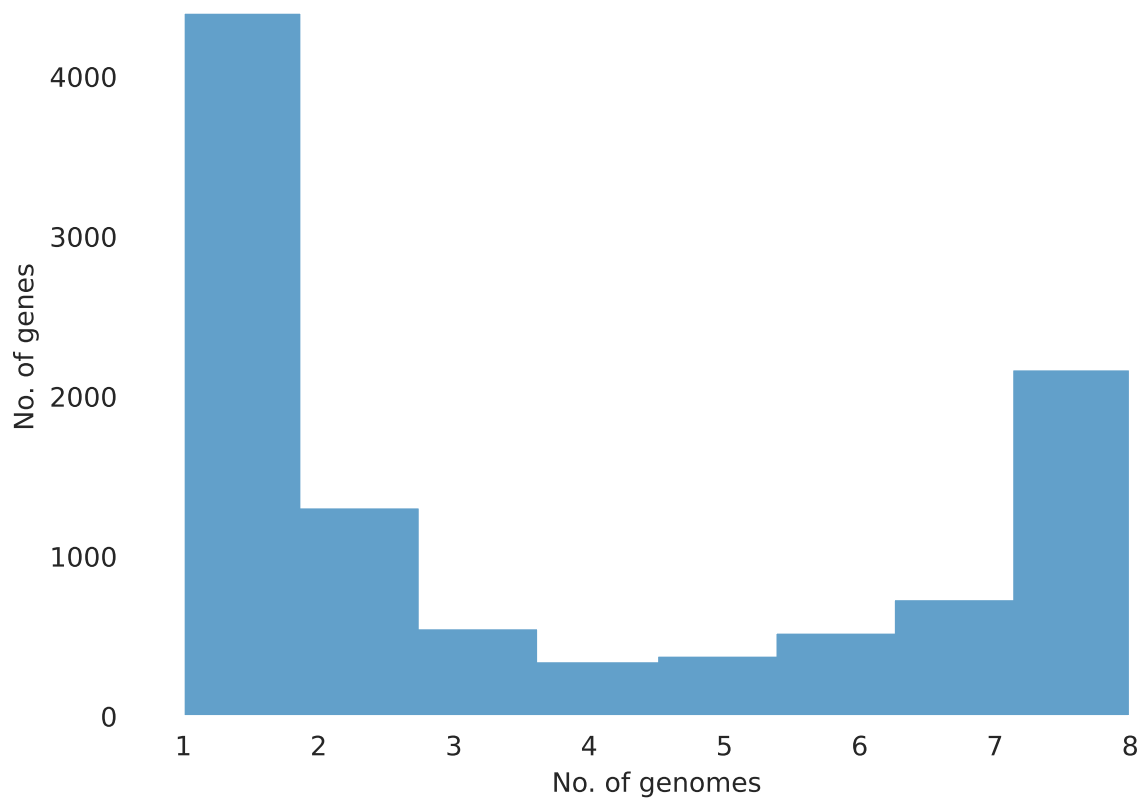

Supplement: S9 File — (ZIP) [file pone.0333844.s015.zip › roaryresult/pangenome_frequency.pdf]

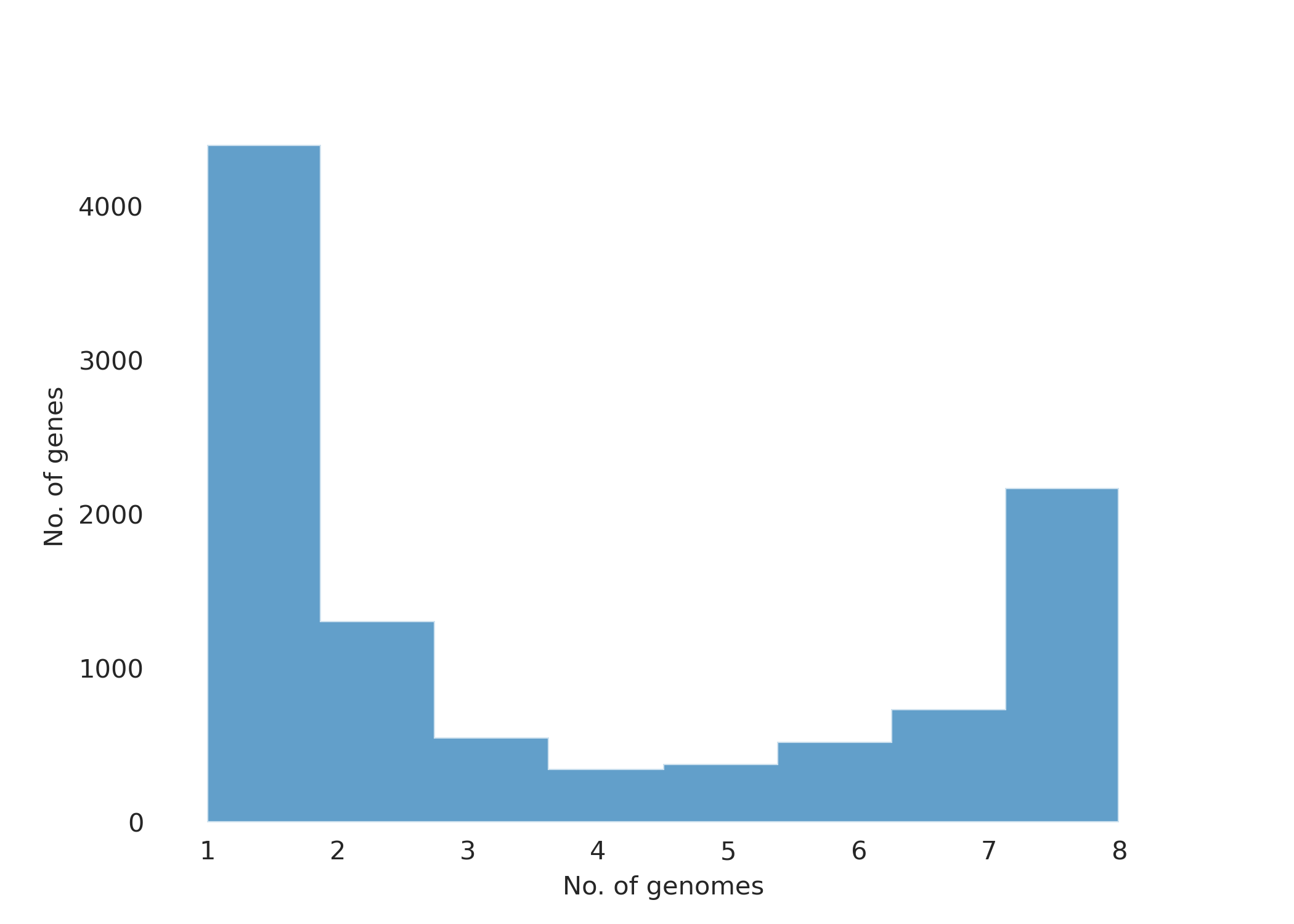

Supplement: S9 File — (ZIP) [file pone.0333844.s015.zip › roaryresult/pangenome_frequency.png]

Tree  
(8 strains)

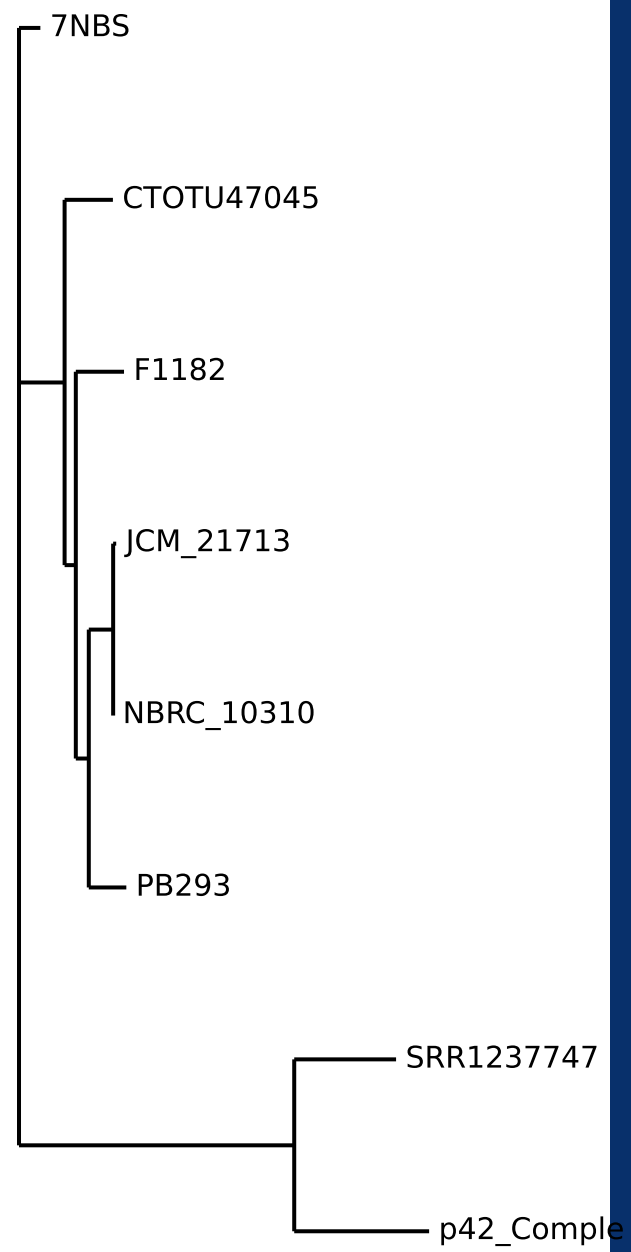

Roary matrix  
(10426 gene clusters)

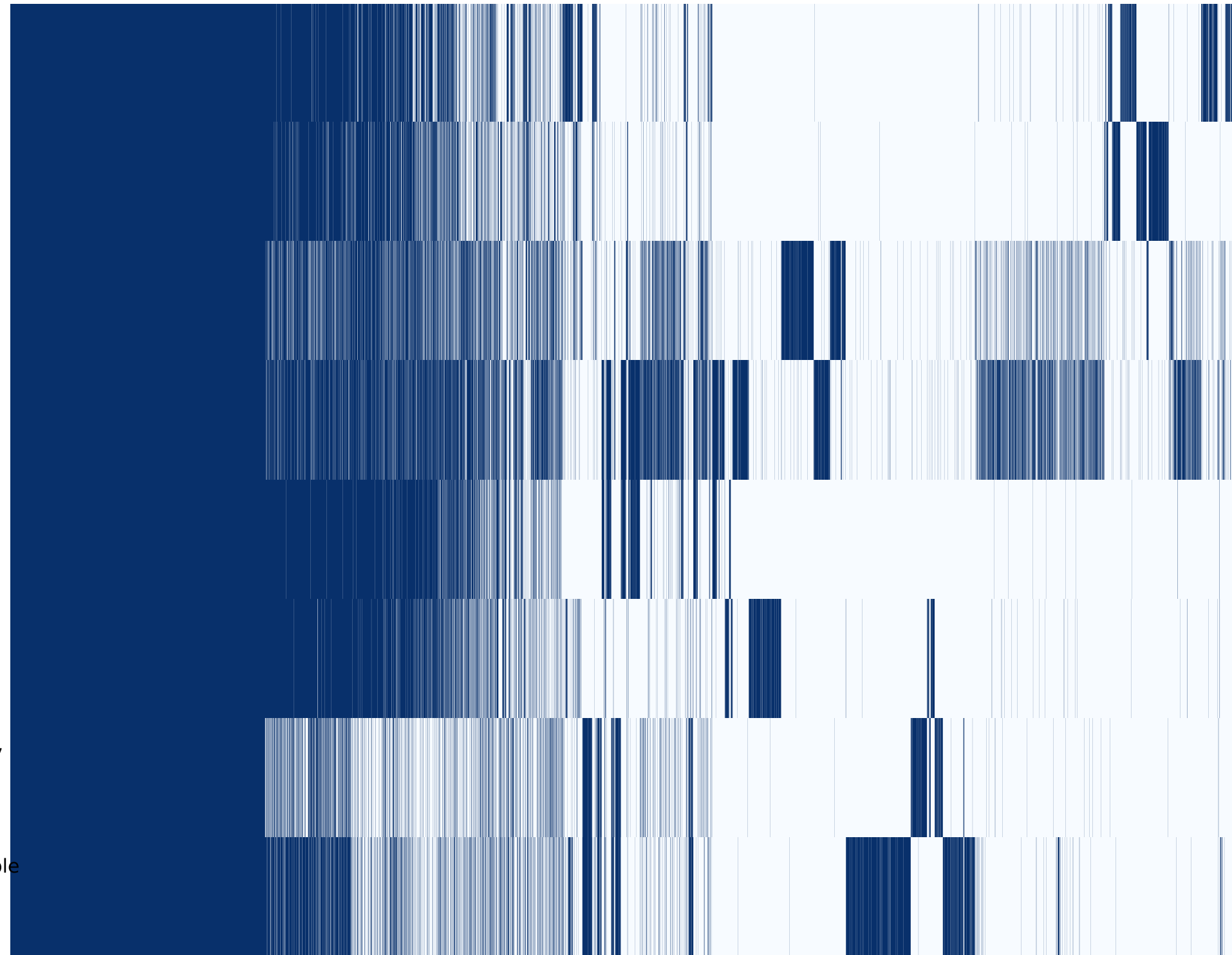

Supplement: S9 File — (ZIP) [file pone.0333844.s015.zip › roaryresult/pangenome_matrix.pdf]

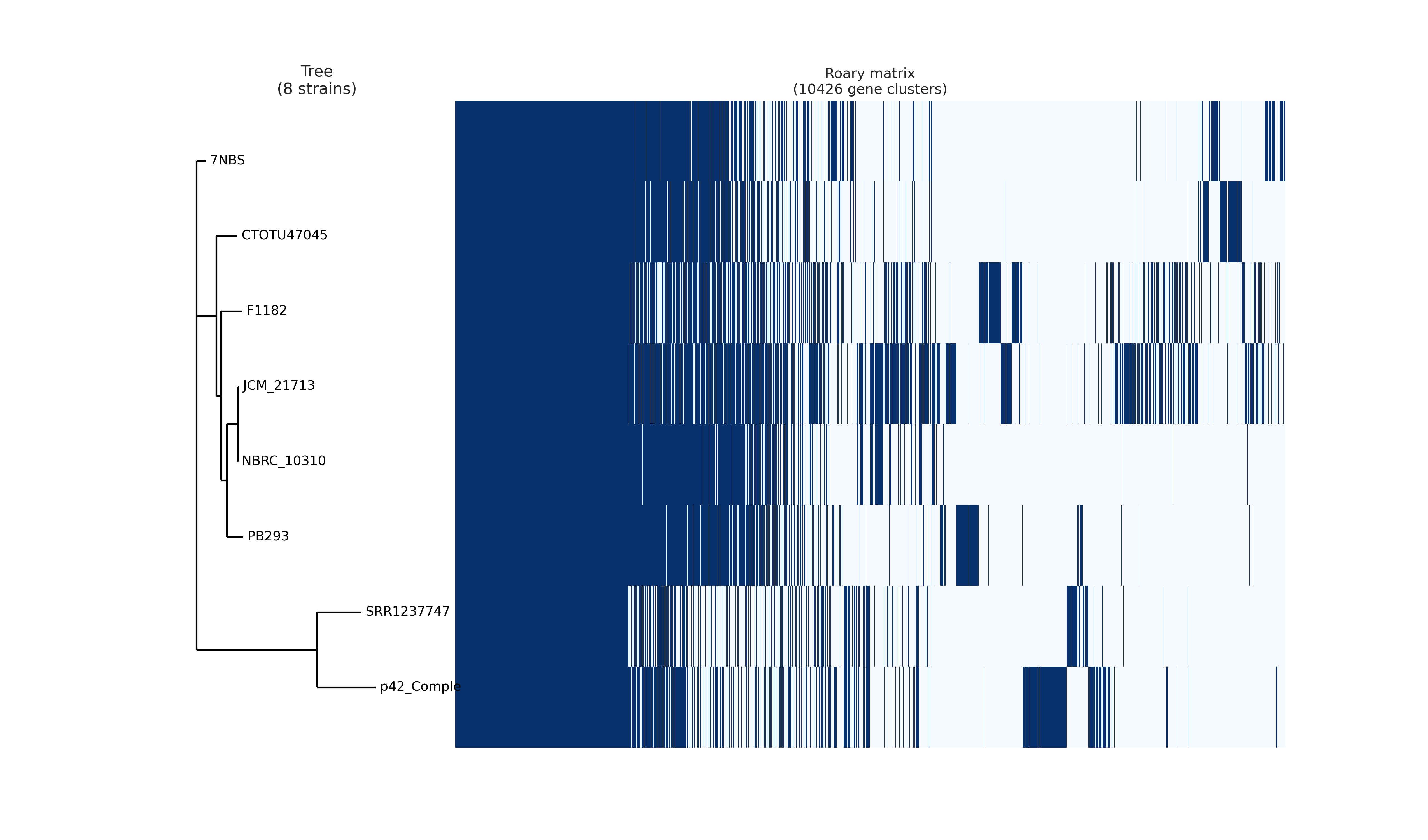

Supplement: S9 File — (ZIP) [file pone.0333844.s015.zip › roaryresult/pangenome_matrix.png]

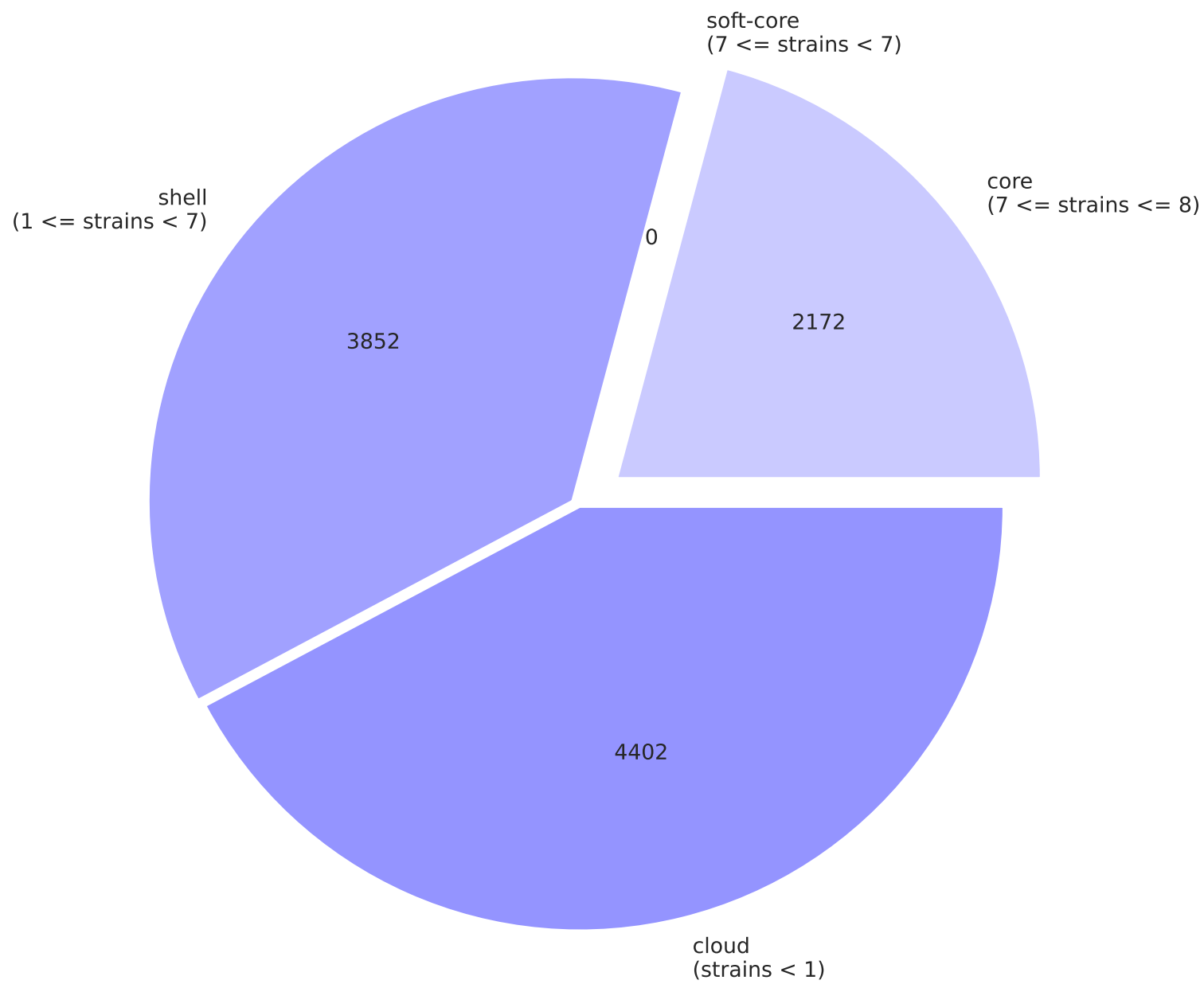

Supplement: S9 File — (ZIP) [file pone.0333844.s015.zip › roaryresult/pangenome_pie.pdf]

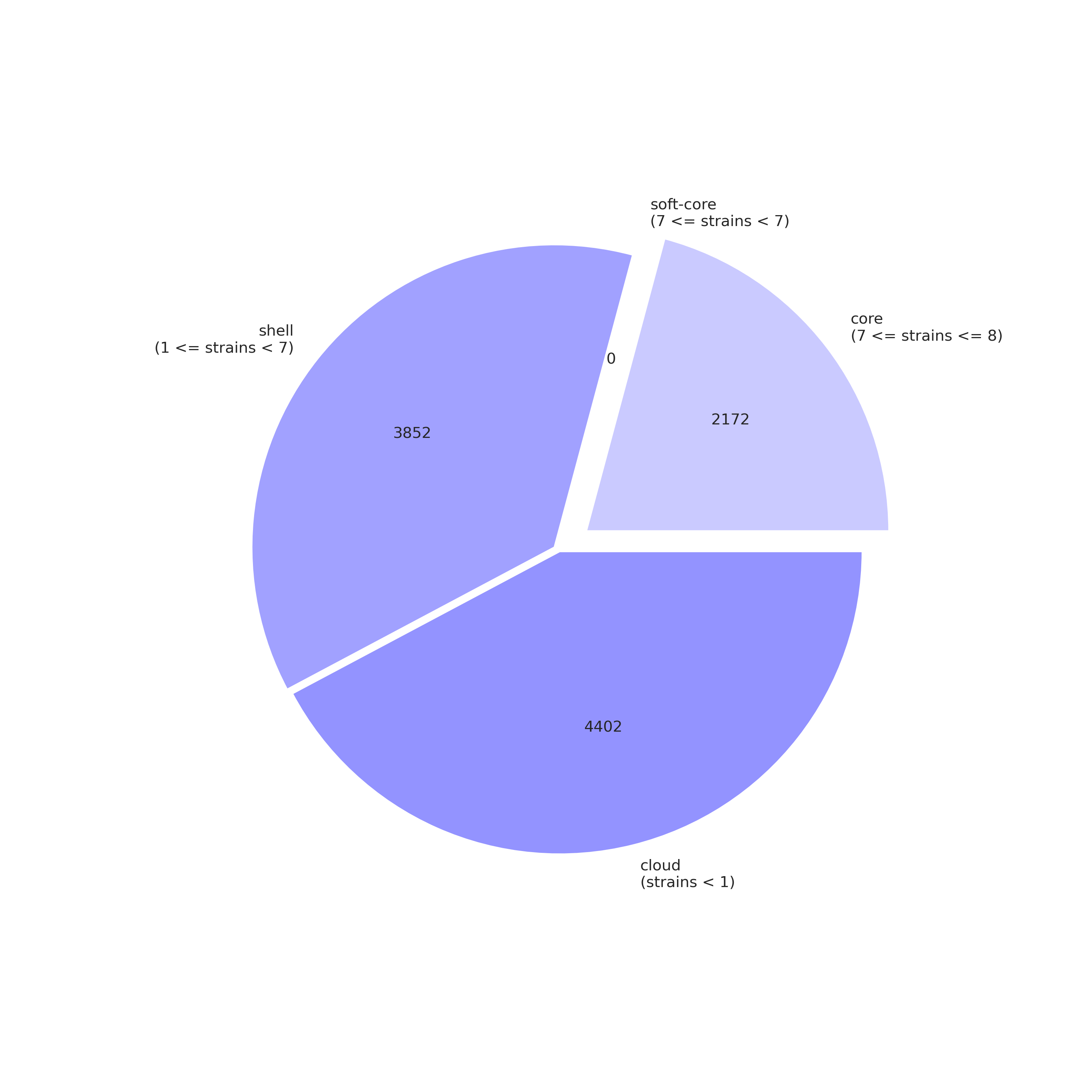

Supplement: S9 File — (ZIP) [file pone.0333844.s015.zip › roaryresult/pangenome_pie.png]
