# Supplementary material for: Whole-genome analysis of Lysinibacillus boronitolerans MSR1: A dairy-isolated multidrug-resistant and non-pathogenic strain
Source: PLoS One. 2025 Dec 12;20(12):e0333844. doi: 10.1371/journal.pone.0333844 (PMC12700380; doi:10.1371/journal.pone.0333844)
Supplement: S1 File — S_Table 1: Absorbance of L. boronitolerans MSR1 growth in different pH. S_Table 2: Genomes of L. boronitolerans retrieved from NCBI Genome Browser. (DOCX) [file pone.0333844.s007.docx]

| Hour | pH-5 | | | pH6 | | | pH-7 | | | pH-8 | | |
| --- | --- | --- | --- | --- | --- | --- | --- | --- | --- | --- | --- | --- |
| 0 | 0 | 0 | 0 | 0 | 0 | 0 | 0 | 0 | 0 | 0 | 0 | 0 |
| 2 | 0 | 0 | 0 | 0 | 0 | 0 | 0 | 0 | 0 | 0 | 0 | 0 |
| 4 | 0 | 0 | 0 | 0 | 0 | 0 | 0 | 0 | 0 | 0.02 | 0.02 | 0.021 |
| 6 | 0 | 0 | 0 | 0 | 0 | 0 | 0.226 | 0.226 | 0.224 | 0.231 | 0.232 | 0.232 |
| 8 | 0.117 | 0.107 | 0.104 | 0.304 | 0.309 | 0.313 | 0.834 | 0.836 | 0.837 | 0.906 | 0.905 | 0.905 |
| 10 | 0.503 | 0.514 | 0.522 | 0.946 | 0.922 | 0.929 | 1.358 | 1.357 | 1.358 | 1.602 | 1.601 | 1.599 |
| 12 | 1.14 | 1.144 | 1.131 | 1.296 | 1.296 | 1.292 | 1.489 | 1.485 | 1.481 | 1.553 | 1.554 | 1.554 |
| 14 | 1.114 | 1.11 | 1.109 | 1.262 | 1.267 | 1.264 | 1.491 | 1.49 | 1.49 | 1.592 | 1.593 | 1.598 |
| 16 | 1.089 | 1.087 | 1.083 | 1.23 | 1.228 | 1.232 | 1.498 | 1.499 | 1.5 | 1.582 | 1.587 | 1.596 |
| 18 | 1.051 | 1.042 | 1.044 | 1.196 | 1.206 | 1.212 | 1.503 | 1.504 | 1.508 | 1.619 | 1.622 | 1.617 |
| 24 | 1.035 | 1.004 | 1.011 | 1.193 | 1.215 | 1.189 | 1.536 | 1.542 | 1.54 | 1.597 | 1.589 | 1.592 |
| 26 | 0.947 | 0.964 | 0.961 | 1.14 | 1.126 | 1.123 | 1.477 | 1.475 | 1.47 | 1.501 | 1.502 | 1.504 |
| 28 | 0.854 | 0.852 | 0.85 | 1.03 | 1.038 | 1.047 | 1.379 | 1.376 | 1.36 | 1.355 | 1.356 | 1.357 |
| 30 | 0.786 | 0.778 | 0.781 | 0.991 | 0.978 | 0.985 | 1.326 | 1.32 | 1.324 | 1.321 | 1.311 | 1.312 |
| 34 | 0.871 | 0.883 | 0.881 | 1.038 | 1.034 | 1.036 | 1.331 | 1.326 | 1.325 | 1.333 | 1.334 | 1.333 |
| 36 | 0.782 | 0.787 | 0.784 | 0.98 | 0.984 | 0.989 | 1.224 | 1.228 | 1.222 | 1.282 | 1.236 | 1.29 |
| 38 | 0.762 | 0.764 | 0.764 | 0.977 | 0.979 | 0.976 | 1.196 | 1.298 | 1.199 | 1.276 | 1.274 | 1.277 |

**S_Table 1: Absorbance of *L. boronitolerans* MSR1 growth in different pH.**

S_Table 2: Genomes of *L. boronitolerans* retrieved from NCBI Genome Browser

| Name of Strain | GeneBank | RefSeq | Genome Size | Level of Assembly |  |
| --- | --- | --- | --- | --- | --- |
| NBRC 103108 | GCA_002200915.1 | GCF_002200915.1 | 4.564 | Contig |  |
| NBRC 103108 | GCA_000772935.1 | GCF_000772935.1 | 4.564 | Contig |  |
| PB293 | GCA_024756155.1 | GCF_024756155.1 | 4.571 | Scaffold |  |
| F1182 | GCA_000286375.1 | GCF_000286375.1 | 4.461 | Contig |  |
| p42 | GCA_030060475.1 |  | 4.682 | Complete |  |
| p42 | GCA_030059785.1 |  | 4.682 | Chromosome |  |
| CTOTU47045 | GCA_031984365.1 | GCF_031984365.1 | 4.382 | Scaffold |  |
| JCM 21713 | GCA_001312025.1 | GCF_001312025.1 | 4.564 | Contig |  |
| SRR12377473_  bin.7_metawrap  _v1.3_MAG | GCA_946403505.1 |  | 3.752 | Contig |  |
| MSR1 | GCA_045033115.1 | GCF_045033115.1 | 4.6 | Scaffold |  |
