# Supplementary material for: Whole-genome analysis of Lysinibacillus boronitolerans MSR1: A dairy-isolated multidrug-resistant and non-pathogenic strain
Source: PLoS One. 2025 Dec 12;20(12):e0333844. doi: 10.1371/journal.pone.0333844 (PMC12700380; doi:10.1371/journal.pone.0333844)
Supplement: S5 File — (DOCX) [file pone.0333844.s011.docx]

Plasmid type HCM1_043

>match start:44815; end:45165; including flanking sequence; direction:forward

GCAAATCGTGCAATATTGGGATAAAAAATTCCATGCAAAAGAAGCACCAATCGAAAAATATATTGATGAAATTCGTTCAACTGTTAAAGAATCTGTAGAGGCTCATCAAATCAGTGATGTAAAAGTTGGTTCATTCTTATCGGGTGGTATTGACTCTAGTTATATTACGTCCTTATTACGCCCAGATAAATCCTTCTCAGTCGGGTTTGCGGACTATGAAGATATGTTCAATGAGACAAATCTTGCAAAAGATTTATCGGATACGTTGAATATTCAAAATGAACGTAGATACATTACAGCAGATGAGTGCTTTGAAGCGCTACCTAAAATTCAATGGCATATGGATGAACCACAATCCAATCCATCTTCAGTGCCACTTTACTTCCTTTCGGAGCTTGCTTCAAAGGATGTAACGGTTGTCCTATCAGGTGAAGGTGCTGATGAAATTTTTGGTGGGTATGCTTGGTATCAAAACTCAGGAAGAATGCAAAAATACGAAAAATTACCATTAGGTCTTCGTAAAACATTACGTGGGATGGCAGAAGCTCTAC

Plasmid type A053

>match start:53880; end:54129; including flanking sequence; direction:forward

ATTAAATATTTTAGCGGACGTGATTATATGGCAGAAAACCTAGACAATGTAGAAATTATTATGAAAGAAATGCTAGAAATACAGCAGAAATCGAAAATGTTTGTTGATCTTTTATCTGAAGGAGAAGCGTTATCACAAAACCAATTAATATTGCTTCTTCAGCTAAAAATAAATAATGGGATGAAGGCAACAGAAATTGCTGAATTCTTTAGTGTAACACCCGGAGCTGTAACTTCCATGTGTGATAAACTGGAAAAACTAGGACTCATACAACGAATTAGAGAAAATAATGATCGACGTGTGGTCAAAATGGCTTTAACCAATACTGGTGACATGAAGGTCCAAGAAATCTTTTTAAAGTTTTCTCAGGATAAACTCATAGACATGGCTAACATCTTACGCGAAGTAAATCAATTAATGAATAAAATTTTTTAGGATTTCAATTTGCTG
