# Supplementary material for: Whole-genome analysis of Lysinibacillus boronitolerans MSR1: A dairy-isolated multidrug-resistant and non-pathogenic strain
Source: PLoS One. 2025 Dec 12;20(12):e0333844. doi: 10.1371/journal.pone.0333844 (PMC12700380; doi:10.1371/journal.pone.0333844)
Supplement: S6 File — (PDF) [file pone.0333844.s012.pdf]

Center for Genomic Epidemiology

Home Services Instructions Output

VirulenceFinder-2.0 Server - Results

Organism(s): *Listeria,Escherichia coli,Enterococcus faecium & Enterococcus lactis,S. aureus,Enterococcus*

Virulence genes for Listeria

Virulence factor	Identity	Query / Template length	Contig	Position in contig	Protein function	Accession number
No hit found						

Shiga-toxin genes

Virulence factor	Identity	Query / Template length	Contig	Position in contig	Protein function	Accession number
No hit found						

Virulence genes for Escherichia coli

Virulence factor	Identity	Query / Template length	Contig	Position in contig	Protein function	Accession number
No hit found						

Hostimm genes for S. aureus

Virulence factor	Identity	Query / Template length	Contig	Position in contig	Protein function	Accession number
No hit found						

Exoenzyme genes for S. aureus

Virulence factor	Identity	Query / Template length	Contig	Position in contig	Protein function	Accession number
No hit found						

Toxin genes for S. aureus

Virulence factor	Identity	Query / Template length	Contig	Position in contig	Protein function	Accession number
No hit found						

Virulence genes for Enterococcus faecium & Enterococcus lactis

Virulence factor	Identity	Query / Template length	Contig	Position in contig	Protein function	Accession number
No hit found						

Virulence genes for Enterococcus

Virulence factor	Identity	Query / Template length	Contig	Position in contig	Protein function	Accession number
No hit found						

extended output

Results as text | Results tsv | Hits in genome seqs | Virulence factor seqs

Input Files: *7NBS.fasta*

CITATIONS

For publication of results, please cite:

- Real-time whole-genome sequencing for routine typing, surveillance, and outbreak detection of verotoxigenic Escherichia coli.
  Joensen KG, Scheutz F, Lund O, Hasman H, Kaas RS, Nielsen EM, Aarestrup FM.
  J. Clin. Micobiol. 2014. 52(5): 1501-1510.
  View the abstract

Support Scientific problems Technical problems

Copyright DTU 2011 / All rights reserved
Center for Genomic Epidemiology, DTU, Kemitorvet, Building 204, 2800 Kgs. Lyngby, Denmark
Contact: Vibeke Dybdahl Hammer, Telephone: +45 3588 6420, E-mail: vdha@food.dtu.dk

Funded by: The Danish Council for Strategic Research
Last modified May 22, 2012 11:08:01 GMT
