# Supplementary material for: Whole-genome analysis of Lysinibacillus boronitolerans MSR1: A dairy-isolated multidrug-resistant and non-pathogenic strain
Source: PLoS One. 2025 Dec 12;20(12):e0333844. doi: 10.1371/journal.pone.0333844 (PMC12700380; doi:10.1371/journal.pone.0333844)
Supplement: S8 File — (ZIP) [file pone.0333844.s014.zip › PAN/Core_Pan_Dot_Plot.pdf]

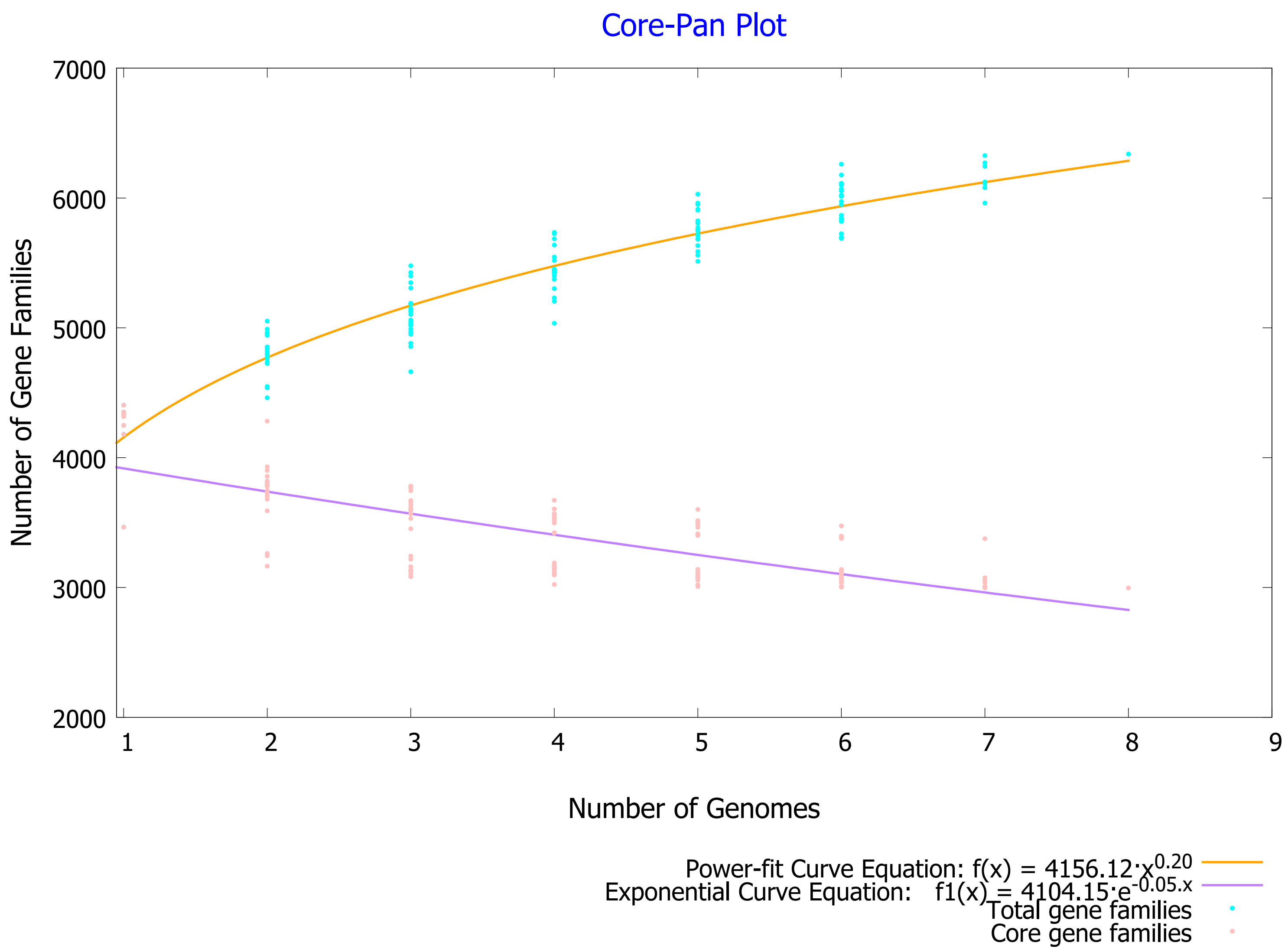

Core-Pan Plot
Number of Gene Families
7000
6000
5000
4000
3000
2000
1
2
3
4
5
6
7
8
9
Number of Genomes
Power-fit Curve Equation: f(x) = 4156.12·x^0.20
Exponential Curve Equation: f1(x) = 4104.15·e^-0.05.x
Total gene families
Core gene families
